# Vitamin A-activated PPARγ signaling enhances intramuscular fat accumulation by overriding AMPK-mediated inhibition in late-fattening beef cattle

**DOI:** 10.1186/s40104-025-01343-1

**Published:** 2026-02-12

**Authors:** Xinyue Yang, Chengxing Zhang, Jizhe Tan, Jinge Zhang, Junhao Cui, Yating Fan, Nan Wang, Yongcheng Jin, Dongqiao Peng

**Affiliations:** 1 College of Animal Sciences, Jilin University, Changchun, 130062 China; 2 Jilin Province Changchun Haoyue Islamic Meat Co., Ltd., Changchun, 130013 China

**Keywords:** AMPK pathway, Beef cattle, Intramuscular fat accumulation, PPARγ signaling, Vitamin A

## Abstract

**Background:**

Intramuscular fat (IMF) deposition determines beef marbling quality, with current industry practices relying on vitamin A (VA) restriction throughout fattening to enhance marbling development. This study challenges the conventional approach by investigating late-fattening vitamin A supplementation effects on marbling formation in Woking black cattle.

**Results:**

Initial in vitro experiments using bovine skeletal muscle cells (BSMCs) demonstrated that all-*trans*-retinoic acid (ATRA) treatment during late differentiation (0.1–1 μmol/L) enhanced lipid accumulation with upregulated PPARγ and FABP4 expression. In vivo trials with late-fattening VA supplementation (3,000 IU/kg DM) significantly improved marbling grades, achieving 75% high-grade marbling (A3 or above) with enhanced nutritionally beneficial fatty acids including EPA and DHA levels. Large-scale analysis using 336 genetically homogeneous cattle revealed that superior marbling development correlated with serum VA depletion after VA supplementation, indicating active utilization rather than restriction. A4-grade cattle showed significantly lower serum VA levels than A1-grade cattle, with coordinated upregulation of lipogenic proteins (FASN, SCD, ACACA, PPARγ, FABP4). Transcriptomic analysis unexpectedly revealed significant AMPK pathway activation alongside enhanced marbling development, contradicting conventional understanding of AMPK as an adipogenesis inhibitor. Functional validation using AMPK modulators in BSMCs confirmed that while AMPK inhibition (Compound C) dramatically enhanced VA-induced adipogenesis, AMPK activation (AICAR) suppressed lipogenesis, demonstrating AMPK functions as a negative feedback regulator during VA-mediated adipogenesis rather than preventing intramuscular fat accumulation.

**Conclusions:**

Strategic late-fattening VA supplementation enhances marbling development through PPARγ-mediated transcriptional networks, with AMPK serving as a metabolic sensor and negative feedback regulator rather than an absolute inhibitor. This stage-specific intervention achieved superior marbling quality and improved fatty acid composition in Woking black cattle, suggesting potential for optimization of premium beef production. Validation across diverse genetic backgrounds and production systems will be essential for broader industry implementation.

**Graphical Abstract:**

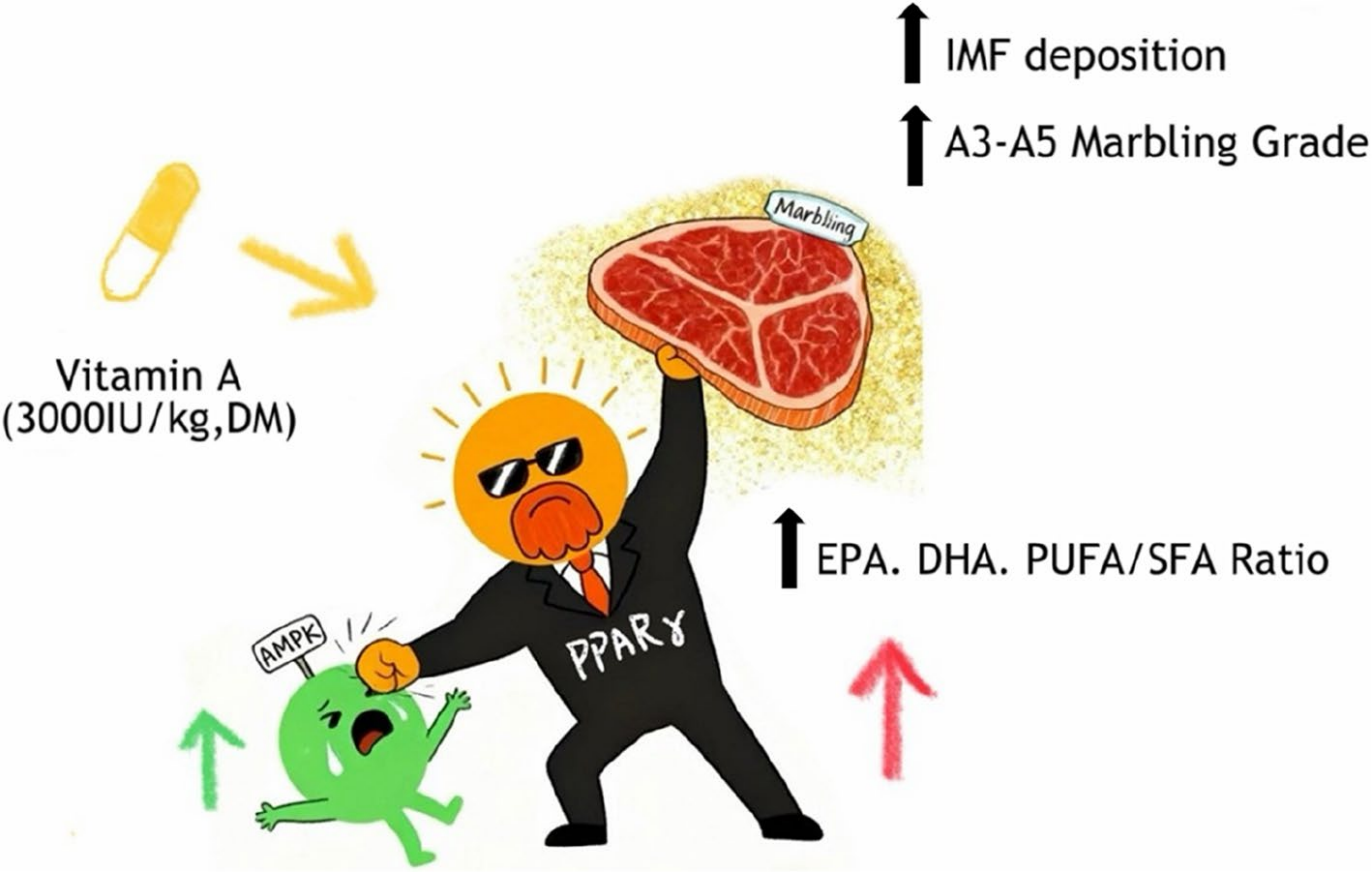

**Supplementary Information:**

The online version contains supplementary material available at 10.1186/s40104-025-01343-1.

## Introduction

With the continuous improvement of consumer living standards, the demand for high-quality meat has increased significantly, especially for premium beef with superior tenderness, flavor, and juiciness in East Asian markets such as China, Japan, and South Korea [1]. Among various quality traits, intramuscular fat (IMF) serves as a key determinant of beef grade and eating quality, and has become a central factor in the production of premium beef [2]. In recent years, nutritional strategies targeting IMF deposition have received increasing attention, with vitamin A (VA) administration standing out due to its regulatory role of adipogenesis and marbling enhancement in beef cattle. Vitamin A has the potential to regulate intramuscular adipose tissue and muscle development, so as to play a crucial role in promoting high-quality beef production [3]. The active metabolite of VA, retinoic acid (RA), can inhibit preadipocyte differentiation and lipid synthesis by activating nuclear receptor pathways involving RAR and RXR [4]. Decades ago, Japanese researchers developed a VA restriction strategy in Japanese black cattle to improve marbling scores based on the negative relationship between vitamin A and marbling score during the fattening period, further they also reported that long-term VA deficiency could lead to hepatic and retinal abnormalities [5]. Although that study raised concerns about the potential risks of VA restriction, the strategy has been widely adopted for years in beef cattle industry. In many cases, VA supplementation was limited throughout the entire fattening period, without accounting for breed- or stage-specific nutritional sensitivity.

However, the relationship between VA and marbling score may not be unconditionally negative. Oka et al. [5] demonstrated that VA supplementation affects carcass marbling only if given during the fattening period, with no significant effect when VA restriction began at 23 or 25 months of age. This stage-specific effect challenges the current industry practice of continuous VA restriction throughout the whole fattening period. Most commercial operations have adopted a uniform approach, restricting VA from early growth until slaughter for operational convenience, potentially overlooking the distinction between early and late fattening phases when the most critical IMF deposition occurs [6].

Subsequent studies have shown that the metabolic effects of vitamin A exhibit clear stage-specific characteristics, and its influence on adipocyte development is not simply inhibitory, but may display dose- and stage-dependent bidirectional regulation [4]. During the early growth stage in animals, moderate VA supplementation is beneficial for the proliferation and maintenance of undifferentiated preadipocytes, laying a cellular foundation for subsequent intramuscular fat (IMF) deposition [7]. Bonet et al. [7] reported that RA maintains adipogenic potential by regulating key transcription factors such as PPARγ and C/EBPα in adipogenic progenitor cells, and that under certain conditions, moderate RA stimulation may even promote lipid synthesis. In pig studies, VA has been shown to be closely related to the expression of stearoyl-CoA desaturase 1 (SCD1) and fatty acid synthase (FASN), influencing both fat deposition and fatty acid composition [8]. In vitro studies of preadipocytes provide more direct evidence. RA treatment significantly upregulated late-stage adipogenic markers such as FABP4, SCD, and FASN, and increased intracellular triglyceride accumulation [9]; whereas both excessive RA stimulation and complete RA signaling inhibition blocked adipogenesis, suggesting an optimal range for RA regulation. More importantly, similar mechanisms have also been observed in bovine-derived adipocytes, where the regulation of proteins such as PPARγ and RXRα by RA was particularly evident [10]. These findings suggest that during critical periods of intramuscular fat accumulation, such as the late fattening stage, moderate VA supplementation may not inhibit IMF deposition, but instead promote the formation of higher marbling grades by activating pathways such as PPARγ in coordination with fatty acid synthesis enzymes.

Our previous research on Woking black cattle suggested that their IMF development pattern may differ from conventional models. We found a significant positive correlation between serum VA levels and marbling grades in Woking black cattle with VA restriction throughout the whole fattening period, in which higher-grade individuals exhibiting higher serum VA concentrations [11]. Furthermore, supplementation with camellia oil during the late fattening period increased VA metabolism and promoted IMF deposition, further challenging the traditional view that lower VA necessarily leads to higher IMF (unpublished data). These absorbing observations, contradicting the established negative relationship between VA and marbling, led us to question whether the traditional paradigm truly applies universally, particularly during the critical late fattening stage when the most significant IMF accumulation occurs.

Based on these preliminary observations, we hypothesized that appropriate VA supplementation during the late fattening stage of Woking black cattle could upregulate the expression of lipogenic genes and enhance IMF deposition, thereby improving marbling grade, which is a concept that would fundamentally challenge current industry practices. This study aims to elucidate the underlying mechanisms and provide a scientific basis for applying precision VA nutrition strategies in high-quality beef production systems and to support the development of premium beef cattle industry.

## Materials and methods

### Isolation of bovine skeletal muscle-derived cells (BSMCs)

BSMC represent a heterogeneous population of primary muscle-derived cells containing multiple cell types including fibro-adipogenic progenitors, satellite cells, and mesenchymal cells, capable of both adipogenic and myogenic differentiation under appropriate culture conditions. The identity and differentiation capacity of BSMC used in this study were previously characterized and validated by our group [12], and the same isolation and culture protocols were applied here with minor modifications. Skeletal muscle sample from the longissimus dorsi muscle between the 12^th^ and 13^th^ ribs of Woking black cattle (30 months of age) was obtained from the slaughterhouse. The tissue was immediately immersed in ice-cold 1 × PBS containing 1 × Gentamicin/Amphotericin B (G/A 100 ×, Yuan Ye, Shanghai, China) and transported under sterile conditions. Under sterile conditions, the muscle tissue was minced into small pieces. Five grams of minced tissue was transferred to 20 mL of digestion solution containing DMEM (Cytiva, Marlborough, USA), supplemented with 0.20% collagenase type II (Yuan Ye, Shanghai, China) and 0.10% Dispase II (Yuan Ye, Shanghai, China). The tissue suspension was incubated at 37 °C with shaking at 90 rpm for 40 min. The enzymatic reaction was terminated by adding 15 mL of DMEM supplemented with 10% FBS (Meilun, Dalian, China) and 1% P/S. The cell suspension was filtered through a 100-μm cell strainer and centrifuged at 1,000 × *g* for 5 min at 20 °C. The pellet was resuspended in 10 mL of red blood cell lysis buffer and immediately centrifuged under the same conditions. After filtration through a 70-μm cell strainer (SORFA, Zhejiang, China), cells were resuspended in 10 mL of complete growth medium consisting of DMEM supplemented with 10% FBS and 1% P/S, and seeded onto 10-cm^2^ culture dishes pre-coated with 0.1% gelatin. Cells were incubated at 37 °C in 5% CO_2_ for 72 h, then the medium was replaced with fresh complete medium supplemented with dual antibiotics (G/A, 20 μL/mL) and changed every 48 h thereafter.

### Adipogenic differentiation and all-*trans*-retinoic acid treatment

When cell density reached 90% confluence, adipocyte differentiation was initiated. Cells were treated with adipogenic differentiation induction medium consisting of DMEM supplemented with 10% HS (horse serum, Meilun, Dalian, China), 1% P/S, 10 μg/mL insulin, 0.5 mmol/L IBMX, 1 μmol/L dexamethasone, and 1 μg/mL rosiglitazone from D0 to D3. From D3 to D11, cells were maintained in differentiation medium consisting of DMEM supplemented with 10% horse serum, 1% P/S, 10 μg/mL insulin, and 1 μg/mL rosiglitazone. Medium was changed every 48 h, and samples were collected on D11. To evaluate the effects of all-*trans*-retinoic acid (ATRA) on adipogenic differentiation during late adipogenic differentiation window from D7 to D11, cells were treated with different concentrations of ATRA. The control group received DMSO vehicle, while treatment groups T1, T2, and T3 received ATRA at final concentrations of 0.01, 0.1, and 1 μmol/L, respectively.

### Oil Red O staining and triglyceride (TG) quantification

On D11, cells were washed with 1 × PBS and fixed with 10% formalin solution for 1 h. After being washed twice with distilled water, the samples were treated with 60% isopropanol for 5 min and air-dried for 10 min. Subsequently, cells were stained with Oil Red O working solution for 30 min at room temperature. The working solution was prepared by dissolving Oil Red O powder in isopropanol at a concentration of 0.005 g/mL, filtering the solution, and then mixing it with ultrapure water in a 3:2 ratio. The final mixture was filtered again through a 0.22-μmol/L membrane. Cellular TG content was quantified using a commercial assay kit (Nanjing Jiancheng Bioengineering Institute, Nanjing, China) according to the manufacturer's instructions.

### Animal and management

All procedures involving animal sample collection and experimental operations in this study were conducted in accordance with the requirements of the Institutional Animal Care and Use Committee (IACUC) of Jilin University (Approval No. SY202302018). All experimental Woking black cattle (F2 hybrids of Australian Wagyu and Yanbian yellow cattle) were provided and maintained by the HAOYUE Group farm (Haoyue Islamic Meat Co., Ltd., HAOYUE Group, Changchun, China). In study 1, 85 Woking black cattle with similar body weight and age (averagely 24 months of age) were selected. Animal feeding and management strictly followed the technical specification for beef cattle feeding and NRC guidelines for beef cattle [13]. To enhance meat quality with marbling, vitamin A restriction was implemented during the fattening period as previously described [14]. The animals were randomly allocated into control group (*n* = 30) and treatment (VA supplementation, *n* = 55) group. During the late fattening period from 24 to 30 months of age, the treatment group received vitamin A supplementation (3,000 IU/kg DM), while the control group remained without additional vitamin A supplementation. Throughout the experiment, cattle had ad libitum access to water and formulated basal diet (as shown in Table 1), then all cattle were slaughtered at 30 months of age. In study 2, a larger-scale experiment (*n* = 502) was conducted to validate the effect of vitamin A supplementation during the late fattening period in Woking black cattle. All cattle underwent vitamin A restriction during the fattening period, followed by vitamin A supplementation during the late fattening period, and were subsequently slaughtered at 30 months of age. All the operations were consistent with our previous study, and all the cattle received same vitamin A supplementation (3,000 IU/kg DM). Carcass traits of Woking black cattle were evaluated according to the Technical Specification for the Measurement of Beef Cattle Production Performance (GB/T 43838–2024, China) in both studies [15]. Marbling grade assessment was conducted by using the standards for Woking black Cattle (Q/HYB0303ND-2017, Changchun, China) [16], classified in ascending order as: A1, A2, A3, A4, and A5. For statistical analyses, these grades were assigned numerical values of 100, 200, 300, 400, and 500, respectively.

**Table 1:** Chemical composition of the experimental diets

Nutrient contents	Concentrate^a^	Forage^b^
Dry matter (DM), %	87.79	88.67
Crude protein, % DM	14.95	3.35
Ether extraction, % DM	2.57	6.44
Crude fiber, % DM	6.25	32.31
Crude ash, % DM	5.94	11.18
Acid detergent fiber, % DM	9.17	44.28
Neutral detergent fiber, % DM	53.05	73.20
Calcium, % DM	1.97	0.78
Phosphorus, % DM	0.67	0.14
Vitamin A, IU/kg	1,879.09	898.40

^a^Commercial concentrate product

^b^Kneaded rice straw

### Collection of longissimus dorsi muscle sample and blood sample

Longissimus dorsi muscle samples were collected immediately after slaughter from between the 12^th^ and 13^th^ ribs of each carcass. The muscle samples were rinsed with DEPC-treated distilled water and rapidly frozen in liquid nitrogen. Samples were transported to the laboratory on dry ice, ground into powder under liquid nitrogen conditions, and stored in 5-mL microtubes at −80 °C for subsequent analysis. Blood samples were collected from the cardiac valve using serum tubes (KONSFI, Hebei, China) containing clotting factors, which were pre-wrapped in aluminum foil to protect from light degradation. After transport to the laboratory, the serum tubes were centrifuged at 2,700 × *g* for 15 min at 4 °C. The supernatant was transferred to pre-wrapped 1.5-mL microtubes and stored at −80 °C for subsequent analysis of serum vitamin A concentration and metabolic profile testing (MPT).

### Identification of *ADH1C* genotype

To determine the exclusive effect of vitamin A on intramuscular adipogenesis in Woking Black cattle, 336 TT-homozygous individuals were selected from a cohort of 502 cattle. This design minimized genetic interference from *ADH1C* polymorphisms, which alter VA metabolic efficiency [17]. The genotypes were determined by PCR–RFLP analysis, and the electrophoretic patterns of the TT and TC genotypes (Fig. S1), clearly distinguished homozygous individuals from heterozygotes based on fragment size distribution. DNA extraction and *ADH1C* genotyping were conducted as described in our previous study with minor modifications [14]. Briefly, genomic DNA was extracted from 0.1 g muscle samples using a manual extraction method. Samples were lysed in TL buffer (10 mmol/L Tris-Cl pH 8.0, 10 mmol/L EDTA pH 8.0, 100 mmol/L NaCl, and 2% sodium dodecyl sulfate) and digested with proteinase K at 56 °C. Following protein precipitation with ammonium acetate solution, DNA was isolated using isopropanol precipitation, washed with 70% ethanol, dried, and resuspended in TE buffer. For genotyping the ADH1C c.−64 T > C locus, PCR amplification was performed using a 1.1 × S4 Fidelity PCR Mix (Genesand, Beijing, China) with specific primers targeting the *ADH1C* gene (NC007304.4, forward primer: 5′-CAGGGCTTAAAGATCCCAGA-3′; reverse primer: 5′-TAGCCAATGCTTGTCTCTCG-3′). PCR conditions included initial denaturation at 98 °C for 2 min, followed by 35 cycles of 98 °C for 10 s, 55 °C for 15 s, and 72 °C for 10 s, with a final extension at 72 °C for 5 min. PCR–RFLP analysis was performed by digesting 10 μL of PCR product with BslI restriction enzyme (New England Biolabs, Hitchin, UK) at 55 °C for 3 h. The C allele was cleaved into 160 and 93 bp fragments [17]. Genotypes were determined by 2% agarose gel electrophoresis, and Woking black cattle with the TT genotype in study 2 (*n* = 336) were selected for further analysis.

### Serum vitamin A analysis

Serum vitamin A analysis was conducted as described previously with minor modifications [18]. Briefly, all analyses were performed in a dark room under red light to prevent vitamin A oxidation. HPLC-grade methanol and n-hexane were used, along with analytical-grade ethanol containing 0.04% butylated hydroxytoluene (BHT) as a stabilizer. For sample preparation, 200 μL of serum was added to a 2.0-mL microtube along with 20 μL of internal standard solution (retinyl acetate, > 99%, diluted into 25 mg/mL by BHT-stabilized ethanol), 200 μL of distilled water (DW), and 400 μL of BHT-stabilized ethanol. After thorough mixing, 800 μL of BHT-stabilized hexane was added for extraction, followed by centrifugation at 3,500 × *g* for 10 min at 4 °C. The supernatant (700 μL) was transferred to a 1.5-mL brown microtube, evaporated under nitrogen gas, and reconstituted with 500 μL of 95% methanol. Samples were then filtered (0.22 μm) and analyzed by HPLC. Chromatographic separation was performed using a C18 column (Elite, Dalian, China) with 95% methanol as the mobile phase at a flow rate of 1 mL/min. Vitamin A was detected at 325 nm with the column temperature maintained at 20 °C. Quantification was accomplished using the ratio of sample areas to the external standard (retinol, > 99%, different concentrations at 12.5, 6.25, 3.13, 1.56, 0.78, 0.39, 0.20, 0.10 and 0.05 μg/mL) curve, with corrections based on internal standard recovery rates. The average recovery exceeded 80%, and all samples were analyzed in duplicate.

### Measuring mRNA expression in longissimus dorsi muscle

Total RNA extraction from longissimus dorsi muscle samples was performed using the TRIzol method as described previously [14]. Briefly, 0.1 g of muscle tissue was homogenized in 1 mL TRIzol reagent (Thermo Scientific, Waltham, USA), followed by chloroform extraction and isopropanol precipitation. After washing with 75% and 100% ethanol, the RNA pellet was vacuum-dried and dissolved in 50 μL DEPC-treated water. RNA concentration and purity were determined using a NanoDrop spectrophotometer (Thermo Scientific, Waltham, USA), with A_260_/A_280_ ratios of 1.8–2.0. RNA integrity was assessed using the Agilent 2100 Bioanalyzer (RNA 6000 Nano Kit, Santa Clara, CA, USA), and samples with RIN ≥ 8.5 were used for subsequent analyses. First-strand cDNA was synthesized using the UnionScript First-strand cDNA Synthesis Kit (Genesand, Beijing, China). The reaction was performed with 1 μg total RNA in a 20 μL mixture, following the manufacturer's protocol with incubation at 37 °C for 2 min, 55 °C for 15 min, and 85 °C for 5 min. The resulting cDNA was diluted to 200 ng/μL for qRT-PCR analysis. Quantitative real-time PCR was conducted using SYBR Green Fast Mix (Genesand, Beijing, China) in a BIO-RAD system. Each 20 μL reaction contained 10 μL 2 × GS AntiQ qPCR SYBR Fast Mix, 0.6 μL each of forward and reverse primers, 2.5 μL cDNA, and 6.3 μL DEPC-treated water. The thermal cycling parameters were: initial denaturation at 95 °C for 3 min, followed by 40 cycles of 95 °C for 10 s, primer-specific annealing temperature for 30 s, and 72 °C for 30 s. Melting curve analysis was performed to verify reaction specificity. Gene-specific primers (Table S1) were designed using the NCBI online system and synthesized by Sangon Biotech (Shanghai, China). Relative mRNA expression was calculated using the 2^−ΔΔCT^ method, with normalization to the geometric mean of three housekeeping genes: *RPS9*, *RPLP0*, and *EIF3K* [12, 14].

### Serum metabolic profile analysis

Serum metabolic parameters were analyzed using commercially available kits from Nanjing Jiancheng Bioengineering Institute (China). The analysis included six parameters: high-density lipoprotein cholesterol (HDL-C), low-density lipoprotein cholesterol (LDL-C), glutamic pyruvic transaminase (GPT), glutamic oxaloacetic transaminase (GOT), glucose (GLU), non-esterified fatty acids (NEFA). All assays were performed according to the manufacturer's instructions, and absorbance measurements were taken using a microplate spectrophotometer (BioTek, USA).

### Fatty acid composition analysis

Fatty acid composition was analyzed as described previously with minor modifications [19]. Briefly, total lipids were extracted from 5 g muscle samples using 20 mL Folch solution (chloroform:methanol, 2:1, v/v) with 100 μL internal standard (methyl tridecanoate in hexane). Samples were incubated overnight at 4 °C with shaking at 180 r/min. After adding 8 mL of 0.88% NaCl solution, samples were further incubated for 2 h at 4 °C, followed by centrifugation at 3,000 × *g* for 15 min at 20 °C. The supernatant (10 mL) was transferred to glass tubes and evaporated under nitrogen at 55 °C. The lipid extract was methylated using 2 mL of methyl ester mixed solution (hexane:BF3:methanol, 20%:35%:45%) at 90 °C for 1 h. After cooling, 2 mL of hexane and 1 mL of 0.88% NaCl were added, followed by centrifugation at 3,000 × *g* for 10 min at 20 °C. The supernatant (1 mL) was transferred to GC vials for analysis. Fatty acid methyl esters were analyzed using a GC7980 gas chromatograph (Techcomp, Shanghai, China) equipped with a silica gel capillary column (100 m × 0.25 mm × 0.20 μmol/L) according to the previously described method [20]. The temperature program was: 70 °C (1 min), increased to 100 °C at 5 °C/min, held for 2 min, increased to 175 °C at 10 °C/min, held for 40 min, increased to 225 °C at 5 °C/min, and held for 40 min. Fatty acids were identified by comparing their retention times with those of standards and quantified as percentages of total fatty acid content based on peak area measurements.

### Protein extraction and western blot analysis

Total protein was extracted from muscle samples (*n* = 10 per group) as previously described with minor modifications [20]. Briefly, 0.1 g of muscle tissue was homogenized in 1 mL RIPA (Genesand, Beijing, China) lysis buffer containing protease inhibitor (PMSF, 100:1) using a pre-cooled tissue homogenizer. After centrifugation at 13,000 × *g* for 10 min at 4 °C, the supernatant was collected and protein concentration was determined using a BCA protein assay kit (Meilun, Dalian, China) according to the manufacturer's instructions. All samples were diluted to a final concentration of 1 μg/μL with PBS and loading buffer, denatured at 100 °C, and stored at −80 °C until analysis. Proteins were separated by SDS-PAGE using a commercial gel preparation kit. Samples (10 μg total protein/well) were loaded alongside a molecular weight marker and membranes were blocked with a protein-free rapid blocking solution (Epizyme, Shanghai, China). Electrophoresed at 80 V for 25 min through the stacking gel, followed by 120 V for 90 min through the separating gel. Proteins were then transferred to polyvinylidene fluoride (PVDF) membranes using a wet transfer method at 350 mA for 45–60 min. After transfer, membranes were blocked with protein-free blocking solution for 1–2 h at room temperature, followed by overnight incubation with primary antibodies (Table S2) at 4 °C. Membranes were washed three times with TBST (10 min each), incubated with appropriate secondary antibodies (Table S2) for 1 h at room temperature, and washed again three times with TBST (Meilun, Dalian, China). Immunoreactive bands were visualized using ECL chemiluminescence reagent (Meilun, Dalian, China). Band intensities were quantified using ImageJ software and normalized to β-actin.

### Analysis of RNA-sequencing data

Total RNA for RNA-sequencing was extracted as previously described and purified with RNAClean XP Kit (A63987, Beckman Coulter) and RNase-Free DNase Set (79254, Qiagen). RNA-seq library construction and sequencing were performed by Kaitai Bio (Hangzhou, China) using the Illumina NovaSeq platform with 150-bp paired-end reads following standard rRNA depletion protocols.

Raw reads were quality-filtered using Fastp v0.20.0, removing low-quality reads and reads shorter than 50 bp. Clean reads were mapped to the bovine reference genome (*Bos taurus* ARS-UCD1.2) using HISAT2 v2.1.0, and transcripts were assembled using StringTie v1.3.6. Gene expression levels were quantified as FPKM (Fragments Per Kilobase Million) using HTSeq v0.13.5. Differentially expressed genes (DEGs) were identified using edgeR with thresholds of FDR < 0.05 and |log_2_ fold change| > 1. GO enrichment analysis (http://geneontology.org/) and KEGG pathway analysis (http://www.kegg.jp/) were performed for DEGs.

### Functional verification of AMPK

AMPK inhibitor Compound C (HY-13418A, MedChem Express, Shanghai, China) and AMPK agonist AICAR (HY-13417, MedChem Express, Shanghai, China) were dissolved in DMSO at 10 mmol/L, filter-sterilized through 0.22-μm PVDF membranes, and applied to cell cultures from differentiation D7 at final concentrations of 10 μmol/L (Compound C) and 300 μmol/L (AICAR). Treatments were refreshed every 48 h.

### Statistical analysis

All statistical analyses were performed using SPSS 27.0 (IBM Corp., Armonk, NY, USA). Data are expressed as means ± SE. The BSMC experiment was performed with *n* = 3 biological replicates (cells from different animals). In vivo experiments used *n* = 12 animals per group for serum metabolite analyses, fatty acid profile and protein expression analyses with pooling method; while *n* = 10 per marbling grade for transcriptomic analyses, serum metabolite analyses, fatty acid profile, protein expression analyses with pooling method, and qPCR measurements with technical triplicates, and the vitamin A analyses was conducted for all the experimental cattle. Independent sample *t*-tests were used to determine the effect of vitamin A treatment during the late fattening period on slaughter carcass traits, blood metabolic profile tests, mRNA and protein expression levels, fatty acid profiles, and vitamin A concentrations. GLM procedure with Tukey’s test was performed to evaluate differences among carcass grade groups (A1, A2, A3, A4) for all measured parameters and to analyze cell culture experiments including ATRA dose-response effects, AMPK inhibition, AMPK activation treatments. Chi-square tests were used to assess frequency distributions of categorical variables. Transcriptomic correlation analysis integrated RNA-seq data with phenotypic parameters using Spearman's rank correlation between gene expression (FPKM) and quantitative traits. Significant correlations were defined as |ρ| > 0.6 with FDR-adjusted *P* < 0.05. Protein-level validation of the transcriptomic results was performed using independent sample *t*-tests to validate the expression trends of these genes and pathways. Statistical significance was considered at *P* < 0.05, and 0.05 ≤ *P* < 0.1 was interpreted as a statistical tendency.

## Results

### All-*trans*-retinoic acid enhances lipid accumulation in BSMCs during late adipogenic differentiation phase

**Fig. 1:**
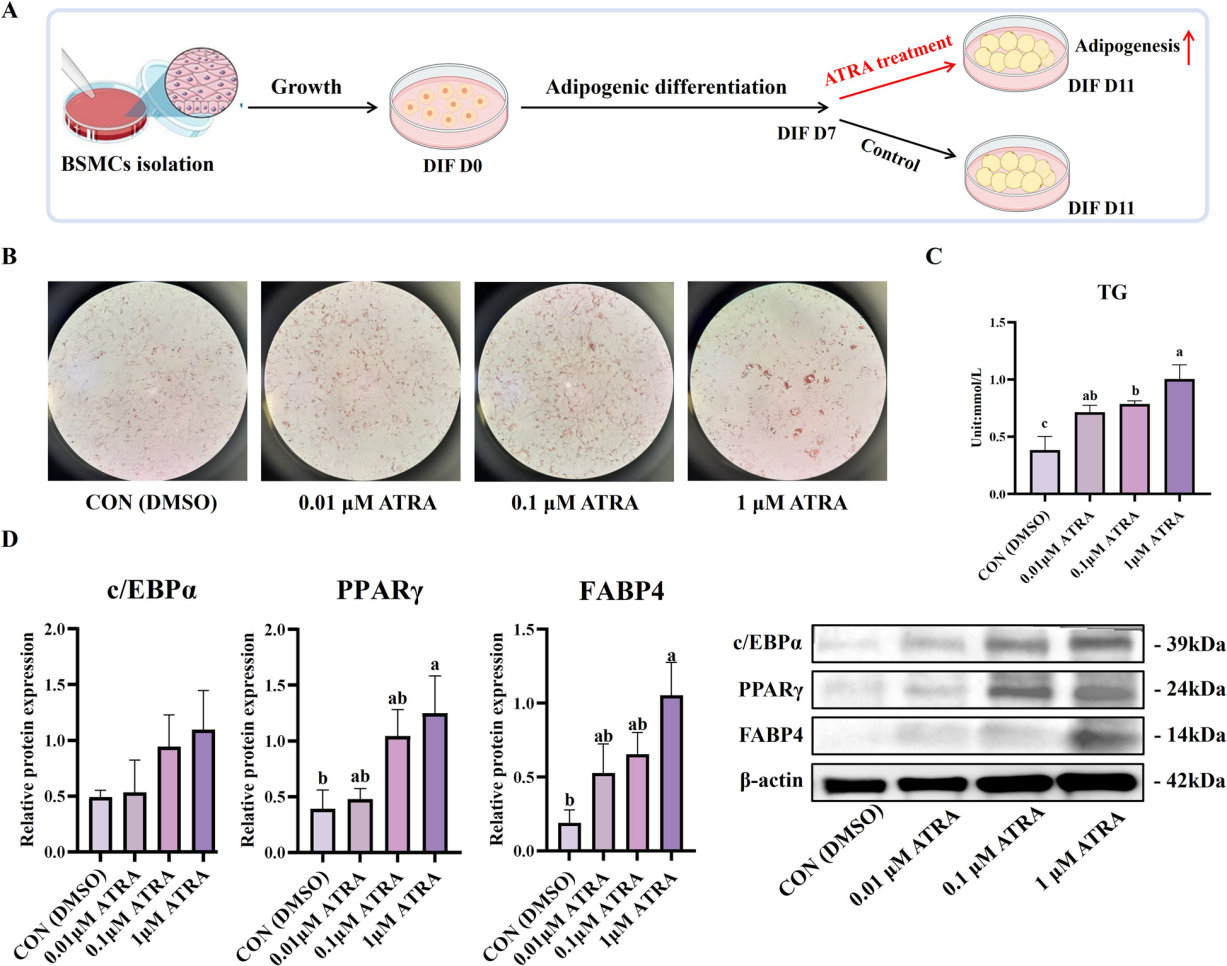
Vitamin A treatment during late adipogenic differentiation stage promotes triglyceride accumulation and adipogenesis-related protein expression in BSMCs of Woking black cattle. **A** Process of vitamin A treatment in BSMCs. BSMCs were treated with different concentrations of vitamin A (0.01, 0.1, 1 μmol/L) during the late differentiation stage (D7–D11), and samples were collected on D11 for analysis. **B** Oil Red O staining images show lipid droplet accumulation (magnification 100 ×). **C** Quantitative results of triglyceride (TG) content. **D** Expression of adipogenesis-related proteins, including PPARγ, C/EBPα and FABP4. Data are presented as the mean ± standard error. ^a^^–^^c^Different lowercase indicates significant differences (*P* < 0.05)

Due to the conflicting evidence regarding the role of retinoic acid in adipogenesis and our preliminary observations suggesting stage-specific effects, we investigated the effects of ATRA on BSMCs isolated from Woking black cattle during late-stage adipogenic differentiation. To test our hypothesis that appropriate ATRA supplementation during late fattening stage could enhance IMF deposition, BSMCs were treated with different concentrations of ATRA (DMSO control, 0.01 μmol/L, 0.1 μmol/L, 1 μmol/L) during the late differentiation stage (D7 to D11, Fig. 1A). ATRA treatment progressively enhanced adipocyte differentiation and lipid accumulation with increasing concentrations (Fig. 1B and C). Oil Red O staining revealed that with increasing ATRA concentration, the number of lipid droplets within cells progressively increased, with enhanced staining intensity and more widespread distribution throughout the cytoplasm. This morphological enhancement was accompanied by significant biochemical changes, as quantitative triglyceride analysis confirmed that TG levels in the ATRA treatment groups were significantly higher than those in the control group (*P* < 0.05), indicating that ATRA effectively promotes lipid synthesis and storage during the terminal phase of adipocyte maturation.

To elucidate the molecular mechanisms underlying these morphological and biochemical changes, we examined the expression of key adipogenic transcription factors and terminal differentiation markers. Western blot analysis revealed concentration-related upregulation of critical adipogenic regulators. PPARγ expression was maximally increased in the 1 μmol/L ATRA group (*P* < 0.05), while FABP4 expression was significantly elevated across all treatment concentrations (*P* < 0.05, Fig. 1D). Notably, C/EBPα expression remained unchanged across all treatment groups, suggesting that promotive effects of ATRA during late-stage differentiation are selectively mediated through specific transcriptional pathways rather than global activation of all adipogenic factors.

These findings demonstrate that ATRA supplementation during the late phase of adipogenic differentiation markedly promotes lipid accumulation in BSMCs, accompanied by upregulation of terminal differentiation markers PPARγ and FABP4. This promotive effect contrasts sharply with previous studies showing that retinoic acid suppresses early adipogenesis by downregulating PPARγ [21], supporting a stage-dependent model where ATRA acts as an inhibitor during early commitment but enhances lipid deposition during terminal adipocyte differentiation [22]. The selective upregulation of PPARγ and FABP4, without corresponding changes in C/EBPα, indicates that ATRA exerts stage-specific effects on adipogenic gene expression, facilitating terminal maturation and enhancing lipid storage capacity. These results support our hypothesis that stage-specific ATRA supplementation could enhance IMF accumulation in Woking black cattle.

### Vitamin A supplementation during late fattening enhances marbling grade and remodels fatty acid composition in beef cattle

**Fig. 2:**
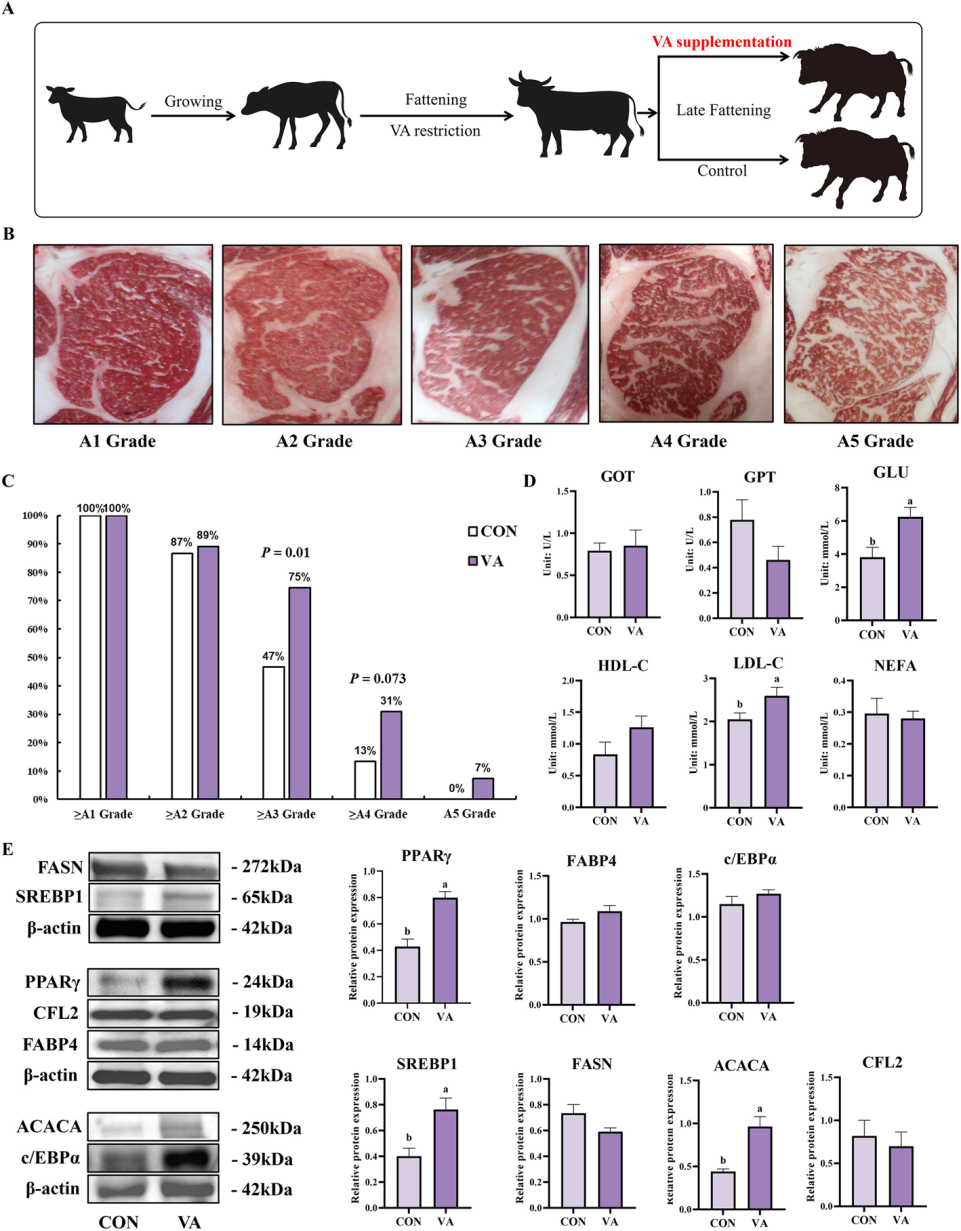
VA supplementation during fattening period enhances marbling score level and adipogenesis markers without disturbing biochemical parameters in Woking black cattle. **A** Flowchart of the experimental design for the Woking black cattle trial. **B** Cross-sectional images of the longissimus dorsi muscle, illustrating the typical distribution patterns of intramuscular fat deposition across different marbling grades (A1–A5). **C** Distribution proportions of different marbling grades (≥ A1, ≥ A2, ≥ A3, ≥ A4, A5) in the control group (CON) and vitamin A supplementation group (VA). **D** Serum metabolic profile analysis test results. **E** Analysis of the expression of fat synthesis-related proteins in the longissimus dorsi muscle. Data are presented as the mean ± standard error; ^*^P < 0.05

To validate our in vitro findings and investigate the effects of vitamin A supplementation on intramuscular fat deposition in vivo, Woking black cattle received vitamin A (3,000 IU/kg DM) during the late fattening period (Fig. 2A). Vitamin A supplementation significantly increased serum vitamin A concentration (*P* < 0.05) and enhanced intramuscular fat deposition as indicated by higher marbling scores (*P* < 0.05, Table 2), while subcutaneous fat thickness was significantly reduced (*P* < 0.05). This selective enhancement of intramuscular versus subcutaneous fat deposition suggests tissue-specific effects of vitamin A during late fattening. Representative images of marbling grades (Fig. 2B) demonstrated clear improvements in intramuscular fat distribution patterns, with vitamin A supplementation significantly increasing the proportion of high-grade marbling (A3 or above,* P* < 0.05) to 75% and facilitating attainment of premium A5 grade marbling (Fig. 2C). All experimental samples achieved at least A1 grade in both groups, but the vitamin A group showed marked advantages in higher grades.

**Table 2:** Effects of vitamin A supplementation during late fattening period on marbling score, carcass traits, and serum vitamin A concentration in Woking black cattle

Item	Control (*n* = 30)	VA supplementation (*n* = 55)	SE	*P*-value
Serum VA concentration, IU/dL	60.0	75.7^**^	3.65	0.005
Slaughter weight, kg	721.1	750.8	14.70	0.108
Carcass weight, kg	439.1	450.9	9.33	0.327
Eye muscle area, cm^2^	54.3	56.5	1.46	0.191
Rib muscle thickness, cm	6.4	6.3	0.11	0.319
Intramuscular fat (marbling score)	241.4	305.6^**^	16.10	0.006
Subcutaneous fat thickness, cm	2.4^*^	2.0	0.14	0.013
Intermuscular fat thickness, cm	3.6	3.5	0.15	0.362

Vitamin A treatment was conducted during late fattening period (24 months of age to 30 months of age) in Woking black cattle

Marbling score grade: 500 = A5, 400 = A4, 300 = A3, 200 = A2, 100 = A1

^*^P < 0.05; ^**^P < 0.01

Beyond morphological improvements, vitamin A supplementation induced comprehensive metabolic remodeling. Analysis of metabolic parameters revealed increased serum LDL-C and glucose concentrations (*P* < 0.05, Fig. 2D), while other parameters remained within normal physiological ranges. More remarkably, fatty acid composition analysis (Table 3) showed that vitamin A supplementation significantly altered the fatty acid profile in a nutritionally favorable manner. Medium-chain saturated fatty acids (SFAs), including caprylic acid (C8:0), capric acid (C10:0), and lauric acid (C12:0) increased significantly (*P* < 0.01). Long-chain SFAs also increased (*P* < 0.05), which led to increase levels of pentadecanoic acid (C15:0) and heptadecanoic acid (C17:0), accompanied by a 13.5% rise in total SFA content (*P* < 0.001). Simultaneously, nutritionally valuable polyunsaturated fatty acids (PUFAs) including linoleic acid (C18:2c n-6), EPA (C20:5 n-3), and DHA (C22:6 n-3) all increased significantly (*P* < 0.001), while alpha-linolenic acid (C18:3c n-3) decreased (*P* < 0.05). These changes collectively resulted in a remarkable increase in total PUFA content (*P* < 0.001) as well as a significantly elevated in the PUFA/SFA ratio. Conversely, oleic acid (C18:1 n9c) and total monounsaturated fatty acids (MUFAs) decreased significantly (*P* < 0.001).

**Table 3:** Effects of vitamin A supplementation on fatty acid profile of longissimus dorsi muscle in Woking black cattle during late fattening period

Fatty acid profiles	Control	VA supplementation	SE	*P*-value
Octanoic acid (C8:0)	0.014	0.097^**^	0.0110	< 0.001
Decanoic acid (C10:0)	0.014	0.046^**^	0.0087	0.006
Hendecanoic acid (C11:0)	0.045	0.045	0.0107	0.973
Lauric acid (C12:0)	0.030	0.050^**^	0.0062	0.009
Myristic acid (C14:0)	2.434	2.604	0.2072	0.488
Myristoleic acid (C14:1)	0.515	0.529	0.0682	0.860
Pentadecanoic acid (C15:0)	0.096	0.239^**^	0.0117	< 0.001
*Cis*-10-pentadecanoic acid (C15:1)	0.027	0.049^**^	0.0041	< 0.001
Palmitic acid (C16:0)	35.348	38.550^*^	0.9581	0.011
Palmitoleic acid (C16:1)	3.003	2.393	0.2436	0.051
Heptadecanoic acid (C17:0)	0.217	0.584^**^	0.0281	< 0.001
*Cis*-10-Heptadecenoic acid (C17:1)	0.252	0.451^**^	0.0273	< 0.001
Stearic acid (C18:0)	3.672	5.157^**^	0.2796	< 0.001
Trans vaccenic acid, TVA (C18:1 n11t)	0.161	0.841^**^	0.0939	< 0.001
Oleic acid (C18:1 n9c)	51.734	43.224^**^	1.1559	< 0.001
Elaidic acid (C18:1 n9t)	0.066	0.248^**^	0.0361	< 0.001
Linolelaidic acid (C18:2t n-6)	0.020	0.129^**^	0.0282	0.003
Linoleic acid (C18:2c n-6)	2.031	4.179^**^	0.2746	< 0.001
*Cis*-9, *trans*-11 CLA	0.045	0.028	0.0071	0.062
*Trans*-10, *cis*-12 CLA	0.004	0.003	0.0008	0.664
γ-Linolenic acid (C18:3c n-6)	0.027	0.037	0.0047	0.062
α-Linolenic acid (C18:3c n-3)	0.040	0.026^**^	0.0041	0.016
Arachidic acid (C20:0)	0.005	0.008	0.0012	0.064
*Cis*-11-Eicosenoicacid (C20:1)	0.039	0.049	0.0128	0.467
*Cis*-11,14-eicosadienoic acid (C20:2c n-6)	0.032	0.024	0.0053	0.170
*Cis*-11,14,17-eicosatrienoic acid (C20:3c n-3)	0.002	0.002	0.0004	0.534
Arachidonic acid, ARA (C20:4c n-6)	0.001	0.002	0.0003	0.209
Eicosapentaenoic acid, EPA (C20:5c n-3)	0.012	0.072^**^	0.0120	< 0.001
Heneicosanoic acid (C21:0)	0.020	0.006^*^	0.0056	0.025
Behenic acid (C22:0)	0.070	0.248^**^	0.0197	< 0.001
Docosahexaenoic acid, DHA (C22:6c n-3)	0.013	0.051^**^	0.0049	< 0.001
Tricosanoic acid (C23:0)	0.002	0.002	0.0003	0.840
Lignoceric acid (C24:0)	0.004	0.010^*^	0.0021	0.015
*Cis*-15-tetracosaenoic acid (C24:1)	0.005	0.017^**^	0.0025	< 0.001
SFA	41.972	47.645^**^	1.0543	< 0.001
MUFA	55.802	47.802^**^	0.9686	< 0.001
PUFA	2.226	4.553^**^	0.2754	< 0.001
MUFA/SFA	1.346	1.009^**^	0.0600	< 0.001
PUFA/SFA	0.054	0.096^**^	0.0063	< 0.001
n-6 fatty acids	2.115	4.374^**^	0.2684	< 0.001
n-3 fatty acids	0.066	0.151^**^	0.0161	< 0.001
n-6/n-3 fatty acids	36.449	33.117	4.1480	0.576

*SFA* Saturated fatty acids, *MUFA* Monounsaturated fatty acids, *PUFA* Polyunsaturated fatty acids

^*^P < 0.05; ^**^P < 0.01

At the molecular level, western blot analysis (Fig. 2E) revealed that vitamin A supplementation selectively upregulated key lipogenic regulators. PPARγ protein expression increased significantly (*P* < 0.05), accompanied by upregulation of the lipogenic transcription factor SREBP1 (*P* < 0.05) and its downstream target ACACA (*P* < 0.05), while FASN levels remained unchanged. As the master regulator of adipocyte differentiation, PPARγ promotes preadipocyte maturation and activates lipid metabolism genes [23], while ACACA encodes the rate-limiting enzyme for fatty acid synthesis [24], and SREBP1 coordinates overall lipid metabolism [25]. These results demonstrate that vitamin A supplementation during late fattening significantly enhances marbling grade while simultaneously improving the nutritional profile of beef through coordinated regulation of lipogenic transcription factors and metabolic remodeling, providing an effective strategy for premium beef production.

### Vitamin A supplementation enables the negative correlation between serum vitamin A levels and marbling grades in slaughtered beef cattle

**Fig. 3:**
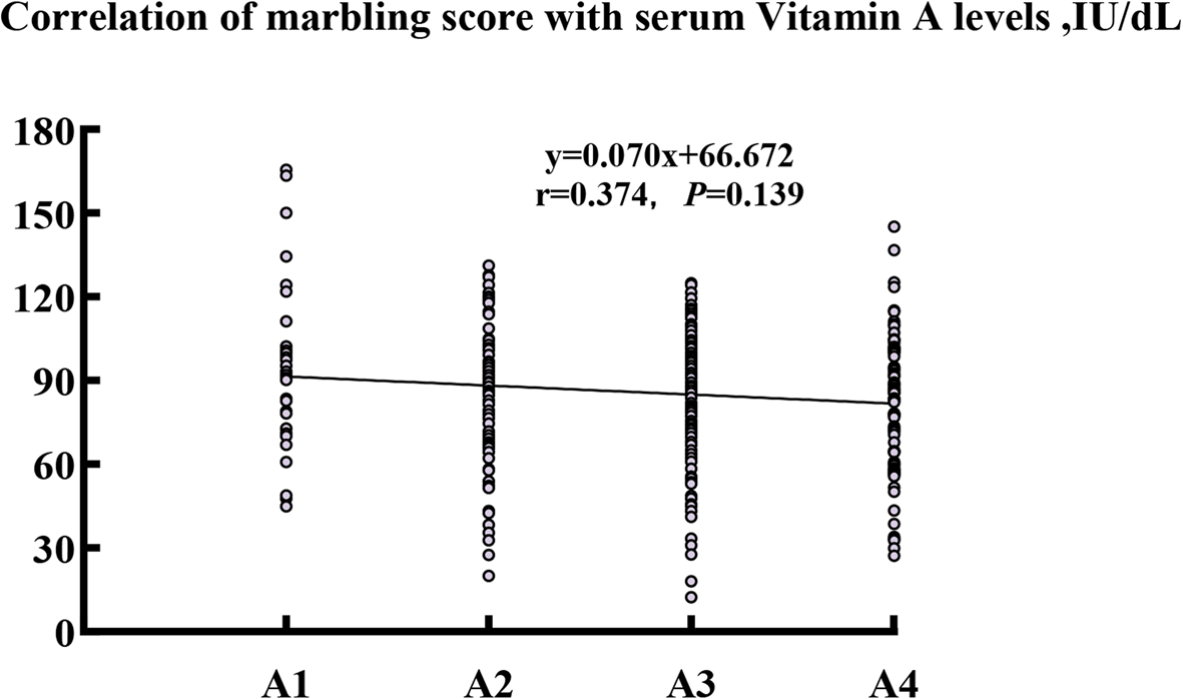
Impact of marbling score grading elevation (A1 to A4) on serum vitamin A levels in Woking black cattle

To validate the relationship between vitamin A supplementation and marbling quality under controlled genetic conditions, we conducted a comprehensive analysis using 336 Woking black cattle homozygous for the ADH1C TT genotype, ensuring uniform vitamin A metabolic capacity across all animals. Importantly, it was only after vitamin A supplementation in late-fattening period of cattle that we observed the anticipated negative correlation between serum vitamin A concentrations and marbling grades (Table 4, Fig. 3). This vitamin A supplementation-enabled relationship demonstrated that serum vitamin A levels decreased progressively from A1 to A4 grades (*P* < 0.05), with A4 cattle showing significantly lower concentrations than A1 cattle. This supplementation-induced vitamin A depletion pattern suggests that enhanced marbling development requires adequate vitamin A provision followed by active consumption during lipogenesis, occurring alongside significant increases in eye muscle area, rib muscle thickness, and intermuscular fat thickness across ascending marbling grades (*P* < 0.05). Cattle with serum vitamin A concentrations between 70 and 90 IU/dL showed higher probability of achieving A3 or above grades, indicating an optimal range for sustained fat deposition.

**Table 4:** Impact of marbling score grading (A1–A4) on carcass traits and serum vitamin A levels in Woking black cattle

Item	Marbling score grade of cattle				SE	*P*-value
	A1	A2	A3	A4		
Serum VA concentration, IU/dL	96.6^a^	86.3^b^	85.8^b^	80.6^b^	6.70	0.033
Slaughter weight, kg	709.9	709.2	706.3	724.5	18.09	0.547
Carcass weight, kg	423.0	420.0	422.1	433.2	12.18	0.425
Eye muscle area, cm^2^	52.3^b^	54.2^b^	54.6^b^	58.9^a^	1.58	< 0.001
Ribeye muscle thickness, cm	5.8^c^	6.1^bc^	6.3^b^	6.5^a^	0.12	< 0.001
Subcutaneous fat thickness, cm	2.1^a^	1.9^b^	2.0^ab^	2.0^ab^	0.16	0.192
Intermuscular fat thickness, cm	3.0^c^	3.2^b^	3.4^b^	3.7^a^	0.12	< 0.001

^a–c^Means with different superscripts in the same row are significantly different (*P* < 0.05)

**Fig. 4:**
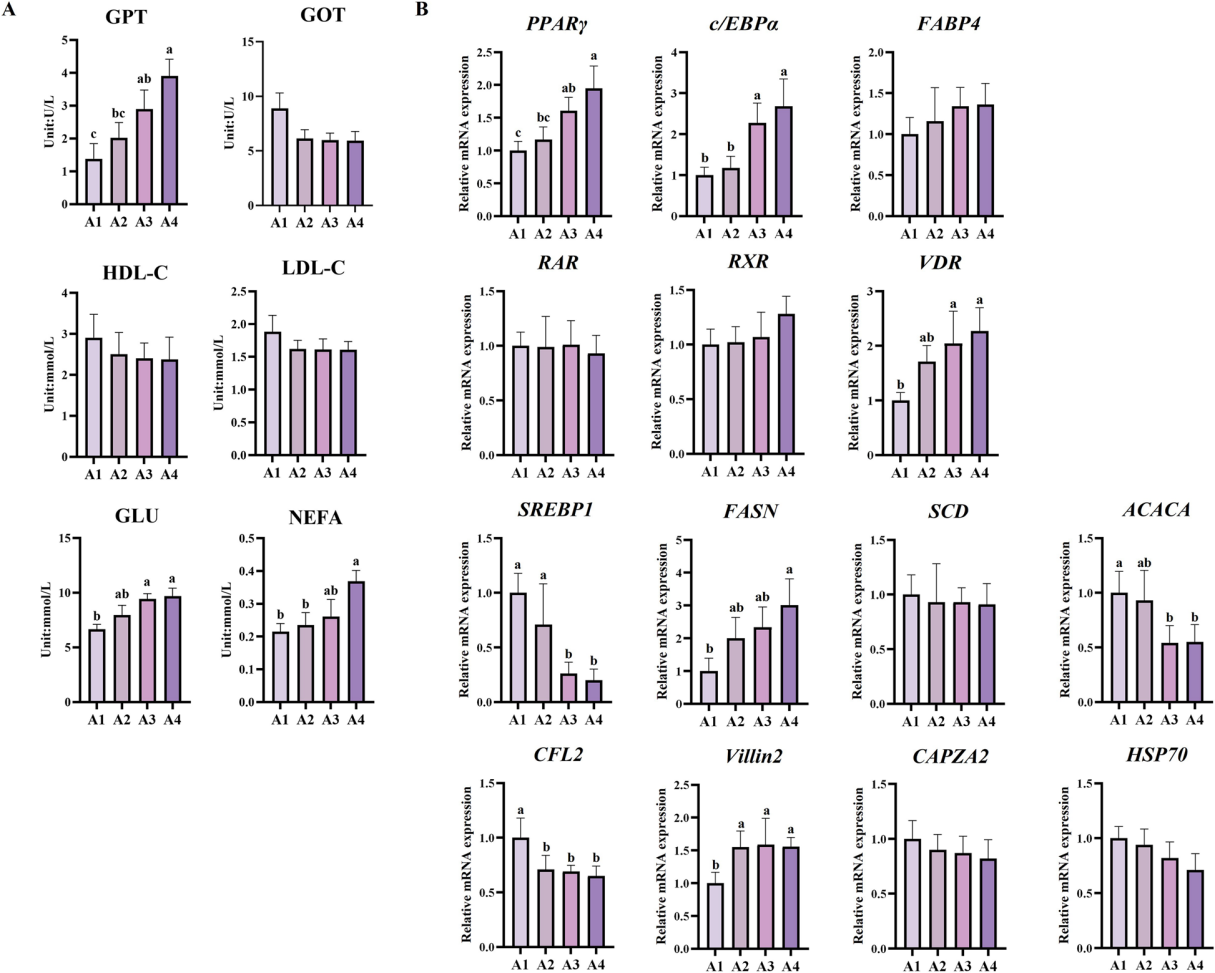
Vitamin A supplementation during late fattening period modulates serum biochemical parameters and adipogenesis-related gene expression in Woking black cattle. **A** Serum metabolic profile analysis test results. **B** Effect of vitamin A supplementation during the late fattening period on mRNA expression of genes related to preadipocyte development, adipogenesis, muscle development, and intramuscular fat deposition. Data are presented as the mean ± standard error. ^a^^–^^c^Different lowercase indicate significant differences (*P* < 0.05)

Biochemical analysis revealed grade-specific metabolic signatures consistent with enhanced lipogenic activity (Fig. 4A). GPT levels decreased significantly with increasing marbling grades (*P* < 0.05), while glucose and NEFA levels showed significant increases (*P* < 0.05). Notably, NEFA levels in A4 cattle were significantly higher than A1 cattle (*P* < 0.05), indicating enhanced lipolytic activity and fatty acid flux associated with superior marbling development.

Fatty acid profiling demonstrated grade-specific compositional improvements that validated enhanced fat synthesis machinery (Table 5). A4-grade beef exhibited significantly higher contents of nutritionally valuable fatty acids, including α-linolenic acid (C18:3 n-3), total n-3 fatty acids, and conjugated linoleic acid (CLA) isomers (*P* < 0.05), along with elevated myristoleic acid (C14:1) and medium-chain saturated fatty acids. The n-6/n-3 ratio was markedly lower in A4-grade than A1-grade beef (*P* < 0.001), while *cis*-9, *trans*-11 CLA accumulation increased progressively with ascending quality grades (*P* < 0.001). These beneficial fatty acid enrichments aligned with enhanced adipocyte maturation processes.

**Table 5:** Effects of marbling score grading (A1–A4) on fatty acid profile of longissimus dorsi muscle in Woking black cattle

Fatty acid profiles	A1 grade	A2 grade	A3 grade	A4 grade	SE	*P*-value
Octanoic acid (C8:0)	0.031^a^	0.027^ab^	0.013^b^	0.023^ab^	0.0066	0.093
Decanoic acid (C10:0)	0.012	0.015	0.008	0.011	0.0036	0.272
Hendecanoic acid (C11:0)	0.040	0.048	0.041	0.048	0.0068	0.568
Lauric acid (C12:0)	0.026^b^	0.032^ab^	0.033^ab^	0.038^a^	0.0034	0.029
Myristic acid (C14:0)	1.846^b^	2.194^ab^	2.590^a^	2.662^a^	0.2257	0.020
Myristoleic acid (C14:1)	0.213^b^	0.267^b^	0.318^b^	0.469^a^	0.0591	0.001
Pentadecanoic acid (C15:0)	0.112^b^	0.129^ab^	0.129^ab^	0.159^a^	0.0142	0.050
*Cis*-10-pentadecanoic acid (C15:1)	0.033^ab^	0.038^a^	0.029^b^	0.030^b^	0.0032	0.031
Palmitic acid (C16:0)	35.825	37.111	37.299	36.868	1.0932	0.692
Palmitoleic acid (C16:1)	1.943	1.939	2.234	2.389	0.2649	0.425
Heptadecanoic acid (C17:0)	0.329	0.361	0.335	0.380	0.0348	0.588
*Cis*-10-Heptadecenoic acid (C17:1)	0.267	0.270	0.253	0.307	0.0287	0.352
Stearic acid (C18:0)	5.298	5.927	5.248	5.298	0.4862	0.628
*Trans* vaccenic acid, TVA (C18:1 n11t)	0.395	0.447	0.491	0.357	0.1298	0.646
Oleic acid (C18:1 n9c)	50.955	48.363	47.987	48.002	1.4372	0.171
Elaidic acid (C18:1 n9t)	0.051	0.059	0.047	0.052	0.0060	0.234
Linolelaidic acid (C18:2t n-6)	0.025	0.027	0.022	0.025	0.0026	0.287
Linoleic acid (C18:2c n-6)	2.256	2.313	2.543	2.400	0.2348	0.669
*Cis*-9, *trans*-11 CLA	0.031^c^	0.043^bc^	0.052^b^	0.095^a^	0.0096	< 0.001
*Trans*-10, *cis*-12 CLA	0.003^b^	0.005^b^	0.005^b^	0.010^a^	0.0012	< 0.001
γ-Linolenic acid (C18:3c n-6)	0.028^b^	0.032^ab^	0.035^ab^	0.038^a^	0.0036	0.035
α-Linolenic acid (C18:3c n-3)	0.016^b^	0.019^b^	0.023^b^	0.064^a^	0.0056	< 0.001
Arachidic acid (C20:0)	0.008^ab^	0.009^a^	0.005^b^	0.006^ab^	0.0021	0.103
*Cis*-11-Eicosenoicacid (C20:1)	0.028	0.041	0.028	0.032	0.0151	0.674
*Cis*-11,14-eicosadienoic acid (C20:2c n-6)	0.044	0.056	0.044	0.043	0.0114	0.517
*Cis*-11,14,17-eicosatrienoic acid (C20:3c n-3)	0.001	0.001	0.001	0.001	0.0003	0.656
Arachidonic acid, ARA (C20:4c n-6)	0.002	0.002	0.001	0.001	0.0004	0.361
Eicosapentaenoic acid, EPA (C20:5c n-3)	0.014^b^	0.020^b^	0.023^ab^	0.032^a^	0.0063	0.013
Heneicosanoic acid (C21:0)	0.015^ab^	0.018^a^	0.013^ab^	0.009^b^	0.0036	0.147
Behenic acid (C22:0)	0.141	0.168	0.132	0.124	0.0351	0.545
Docosahexaenoic acid, DHA (C22:6c n-3)	0.007	0.009	0.010	0.010	0.0013	0.140
Tricosanoic acid (C23:0)	0.002	0.002	0.001	0.002	0.0005	0.353
Lignoceric acid (C24:0)	0.001^b^	0.003^a^	0.002^ab^	0.002^ab^	0.0007	0.131
*Cis*-15-tetracosaenoic acid (C24:1)	0.004^b^	0.006^b^	0.005^b^	0.010^a^	0.0026	0.021
SFA	43.686	46.043	45.850	45.632	1.3548	0.423
MUFA	53.888	51.430	51.392	51.649	1.4077	0.339
PUFA	2.426	2.527	2.758	2.720	0.2428	0.516
MUFA/SFA	1.255	1.124	1.129	1.146	0.0765	0.345
PUFA/SFA	0.056	0.055	0.060	0.060	0.0055	0.737
n-6 fatty acids	2.357	2.434	2.650	2.518	0.2382	0.682
n-3 fatty acids	0.038^b^	0.050^b^	0.056^b^	0.107^a^	0.0109	< 0.001
n-6/n-3 fatty acids	64.266^a^	51.265^b^	46.888^b^	25.574^c^	6.1199	< 0.001

*SFA* Saturated fatty acids, *MUFA* Monounsaturated fatty acids, *PUFA* Polyunsaturated fatty acids

^a–c^Means with different superscripts in the same row are significantly different (*P* < 0.05)

Molecular analysis revealed coordinated regulation of adipogenic pathways with vitamin A-responsive signaling. These gene-expression differences reflect vitamin A-responsive regulation rather than intrinsic differences unrelated to treatment, because all animals analyzed had received vitamin A supplementation during the late fattening period. Gene expression analysis (Fig. 4B) showed significantly higher *PPARγ* and *C/EBPα* expression in A4 versus A1 grades (*P* < 0.05), confirming enhanced adipocyte development. Interestingly, *SREBP1* and *ACACA* were downregulated at mRNA level in A4 cattle (*P* < 0.05), while *FASN* showed significant upregulation, suggesting complex transcriptional regulation. *CFL2* downregulation and villin 2 upregulation (*P* < 0.05) may facilitate intramuscular fat cell recruitment over muscle cell differentiation [26]. *VDR* expression was highest in A4 grade (*P* < 0.05), supporting vitamin A signaling through nuclear receptor pathways in intramuscular fat development [27].

**Fig. 5:**
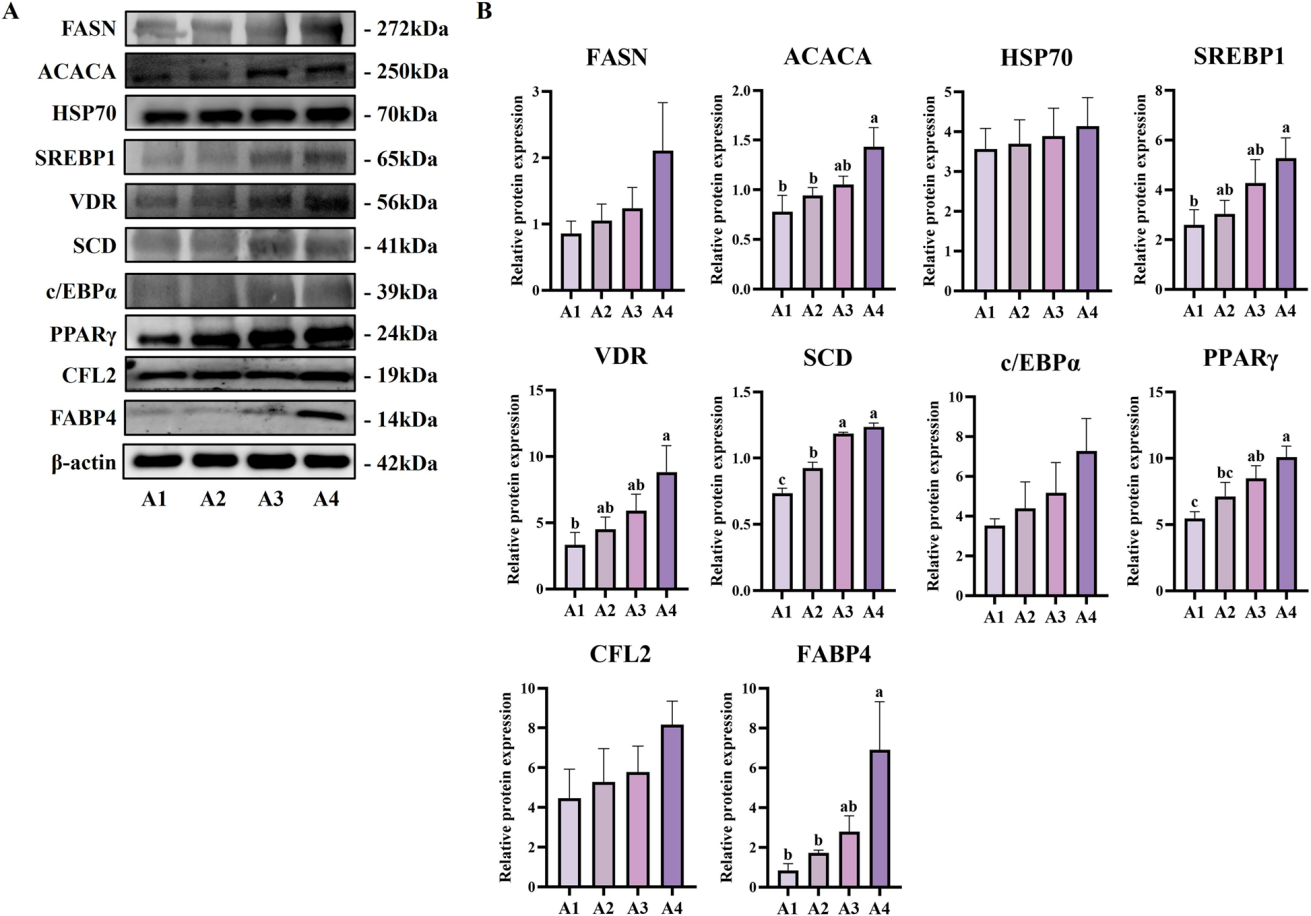
Vitamin A supplementation during late fattening period enhances adipogenic protein expression in Woking black cattle. **A** Western blot analysis of lipid synthesis and transcription regulation-related proteins in samples of the longest back muscle with different marble patterns (A1–A4). **B** Statistical results of the relative expression levels of the corresponding proteins. Data are presented as the mean ± standard error. ^a^^–^^c^Different lowercase indicate significant differences (*P* < 0.05)

Protein expression analysis confirmed coordinated upregulation of lipogenic machinery with complex post-transcriptional regulation (Fig. 5A and B). FASN, SCD, ACACA, PPARγ, and FABP4 proteins were all significantly elevated in A4 cattle (*P* < 0.05). Notably, SREBP1 showed increased protein expression in A4 cattle despite mRNA downregulation, indicating complex post-transcriptional regulation potentially associated with metabolic adaptation. VDR protein expression was highest in A4 cattle (*P* < 0.05), confirming enhanced vitamin A-responsive transcriptional activity in superior marbling grades. These results demonstrate that late-fattening vitamin A supplementation creates applicable conditions for enhanced marbling development through coordinated metabolic regulation and active vitamin A utilization in adipogenesis.

### Transcriptomic analysis reveals paradoxical AMPK activation alongside enhanced marbling development

**Fig. 6:**
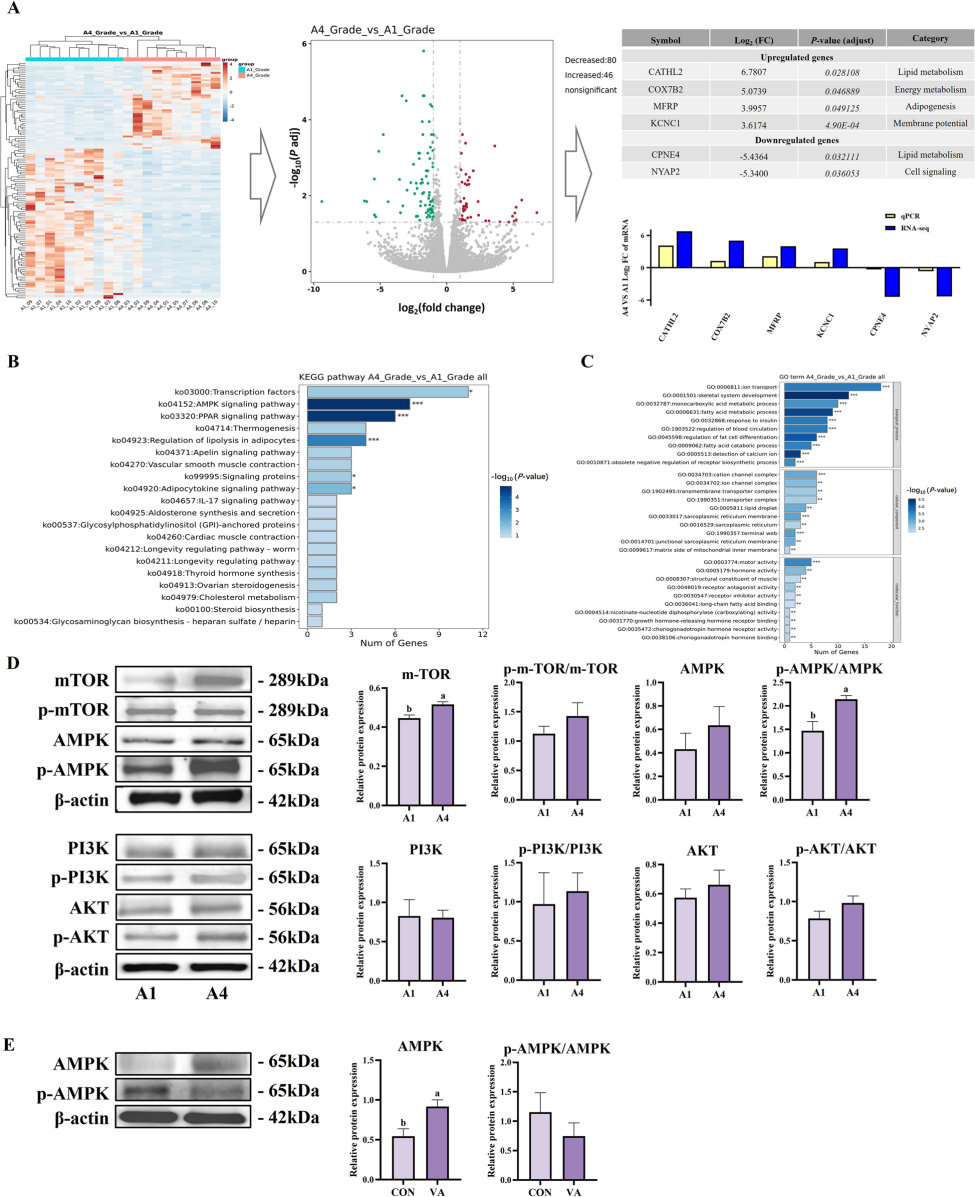
Integrated transcriptomic profiling and proteomic verification reveal key adipogenic pathways underlying marbling grade differences (A1 vs. A4) in longissimus dorsi muscle of Woking black cattle. **A** Integrated RNA-seq and qPCR validation of differentially expressed genes (DEGs) between A1-grade (low marbling) and A4-grade (high marbling) groups. Left: hierarchical clustering heatmap of DEGs; middle: volcano plot showing 80 downregulated and 46 upregulated genes in A4 vs. A1 (cutoff: |log_2_FC| > 1.5, FDR < 0.05); right: selected DEGs with corresponding log₂FC and adjusted *p*-values. Bottom: qPCR and RNA-seq log_2_FC comparisons for seven DEGs. **B** KEGG pathway enrichment analysis of DEGs reveals significant activation of AMPK signaling, adipocytokine signaling, thermogenesis, and lipid metabolism-related pathways in A4-grade samples. **C** Gene Ontology (GO) enrichment analysis indicates A4-grade group exhibits significant changes in oxidoreductase activity, mitochondrial inner membrane components, and protein transport processes. **D** Western blot validation of key signaling proteins in longissimus dorsi tissue. **E** Protein expression levels of AMPK and phosphorylated AMPK in longissimus dorsi of control (CON) and vitamin A-supplemented (VA) cattle. Data are presented as the mean ± standard error; ^*^P < 0.05

To further elucidate the molecular regulatory mechanisms underlying fat deposition in muscle tissue of Woking black cattle with different marbling levels, we performed transcriptomic sequencing on longissimus dorsi muscle tissues from A1 and A4 grade cattle (*n* = 10 per grade). RNA-seq analysis identified 126 DEGs between A1 and A4 grades (46 upregulated, 80 downregulated; FDR < 0.05, |log_2_ fold change| > 1; Additional file 2), with volcano plots and heatmaps revealing significant expression differences (Fig. 6A). Among these DEGs, six key genes associated with lipid metabolism and cellular energy regulation were selected for qPCR validation based on their biological relevance and expression magnitude. These genes included *CATHL2* (cathelicidin antimicrobial peptide-like 2), *COX7B2* (cytochrome C oxidase subunit 7B2), *KCNC1* (potassium voltage-gated channel subfamily C member 1), and *MFRP* (membrane frizzled-related protein), which were significantly upregulated in A4-grade samples, while *CPNE4* (Copine IV) and *NYAP2* (neuronal tyrosine-phosphorylated phosphoinositide-3-kinase adapter 2) were downregulated. qPCR validation confirmed consistent expression trends with the RNA-seq results, validating the reliability of the transcriptomic analysis.

KEGG pathway enrichment analysis revealed an unexpected metabolic signature: significant activation of the PPARγ and AMPK signaling pathway in A4-grade samples (Fig. 6B), despite AMPK being traditionally recognized as a central inhibitor of anabolic processes including fatty acid synthesis [28, 29]. While AMPK is widely recognized as a central regulator of energy homeostasis, the present study focused on regulatory relationship of AMPK with lipogenic pathways during late-stage adipocyte differentiation, where triglyceride synthesis rather than lipid mobilization represents the dominant metabolic activity. AMPK activation observed in this context was therefore interpreted as a compensatory response to enhanced lipogenic flux. Western blot analysis confirmed this paradoxical relationship between AMPK activity and marbling grade (Fig. 6D). Contrary to conventional understanding, p-AMPK expression was significantly higher in A4-grade samples compared to A1-grade samples (*P* < 0.05), despite A4 cattle having substantially more intramuscular fat. The phosphorylated forms of PI3K, AKT, and mTOR showed no significant differences between groups, indicating that this AMPK activation was not part of a general shift in energy-sensing pathways. Similarly, vitamin A supplementation significantly increased p-AMPK expression compared to controls (Fig. 6E), presenting the striking contradiction where enhanced intramuscular fat deposition and AMPK activation occur concomitantly.

Analysis of differentially expressed genes provided mechanistic insights into this unexpected regulation. The upregulated *CATHL2*, an innate immunity-related peptide, may modulate lipid accumulation by inhibiting inflammation and promoting preadipocyte differentiation through systemic energy metabolism regulation [30]. Enhanced *COX7B2* expression, encoding a mitochondrial cytochrome c oxidase subunit, suggests improved oxidative phosphorylation efficiency to meet increased energy demands [31]. *KCNC1* upregulation may contribute to adipocyte membrane potential stability and insulin sensitivity regulation [32], while *MFRP* potentially influences lipid deposition through *Wnt* signaling modulation during preadipocyte differentiation [33]. The concurrent downregulation of *CPNE4* and *NYAP2*, both negative regulators of lipid metabolism, may relieve inhibitory constraints on lipogenic pathways, thereby facilitating intramuscular fat accumulation.

These findings reveal an unexpected metabolic paradigm where AMPK activation coincides with enhanced marbling development, suggesting that in the context of vitamin A supplementation and intensive lipogenesis by activation of the PPARγ, AMPK may function as a metabolic sensor responding to increased energy demands rather than acting as a conventional inhibitor of fat synthesis. This paradoxical activation may represent an adaptive mechanism to maintain energy homeostasis during periods of enhanced intramuscular fat deposition, highlighting the complexity of metabolic regulation in tissue-specific adipogenesis.

### AMPK functions as a negative feedback regulator during vitamin A-induced adipogenesis in BSMCs

**Fig. 7:**
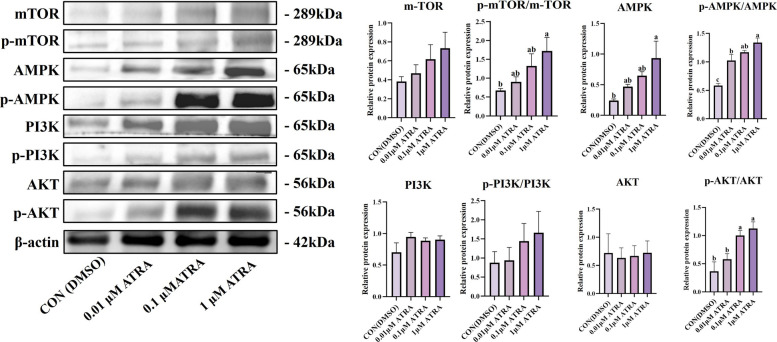
ATRA activates adipogenic pathways to promote triglyceride accumulation and marker expression in BSMCs of Woking black cattle. Analysis of signaling pathway-related protein expression, including energy sensing and synthesis signaling pathway proteins (AMPK, p-AMPK, mTOR, p-mTOR, PI3K, p-PI3K, AKT, p-AKT). Data are presented as the mean ± standard error. ^a–c^Different lowercase indicate significant differences (*P* < 0.05)

To validate whether the paradoxical AMPK activation observed in vivo operates through similar mechanisms at the cellular level, we performed complementary experiments using BSMCs. ATRA treatment of BSMCs recapitulated the in vivo signaling patterns, with significant upregulation of both AMPK and p-AMPK in the 1 μmol/L vitamin A group (*P* < 0.05, Fig. 7). The p-mTOR/mTOR ratio also increased significantly in the 1 μmol/L group (*P* < 0.05), consistent with established role of mTOR in adipogenesis and cell growth [34]. Additionally, p-AKT was significantly upregulated in high vitamin A concentration groups (*P* < 0.05). These findings confirmed that the activation patterns observed in vitro were consistent with those detected in vivo, with AMPK exhibiting the most pronounced changes among the pathways examined.

**Fig. 8:**
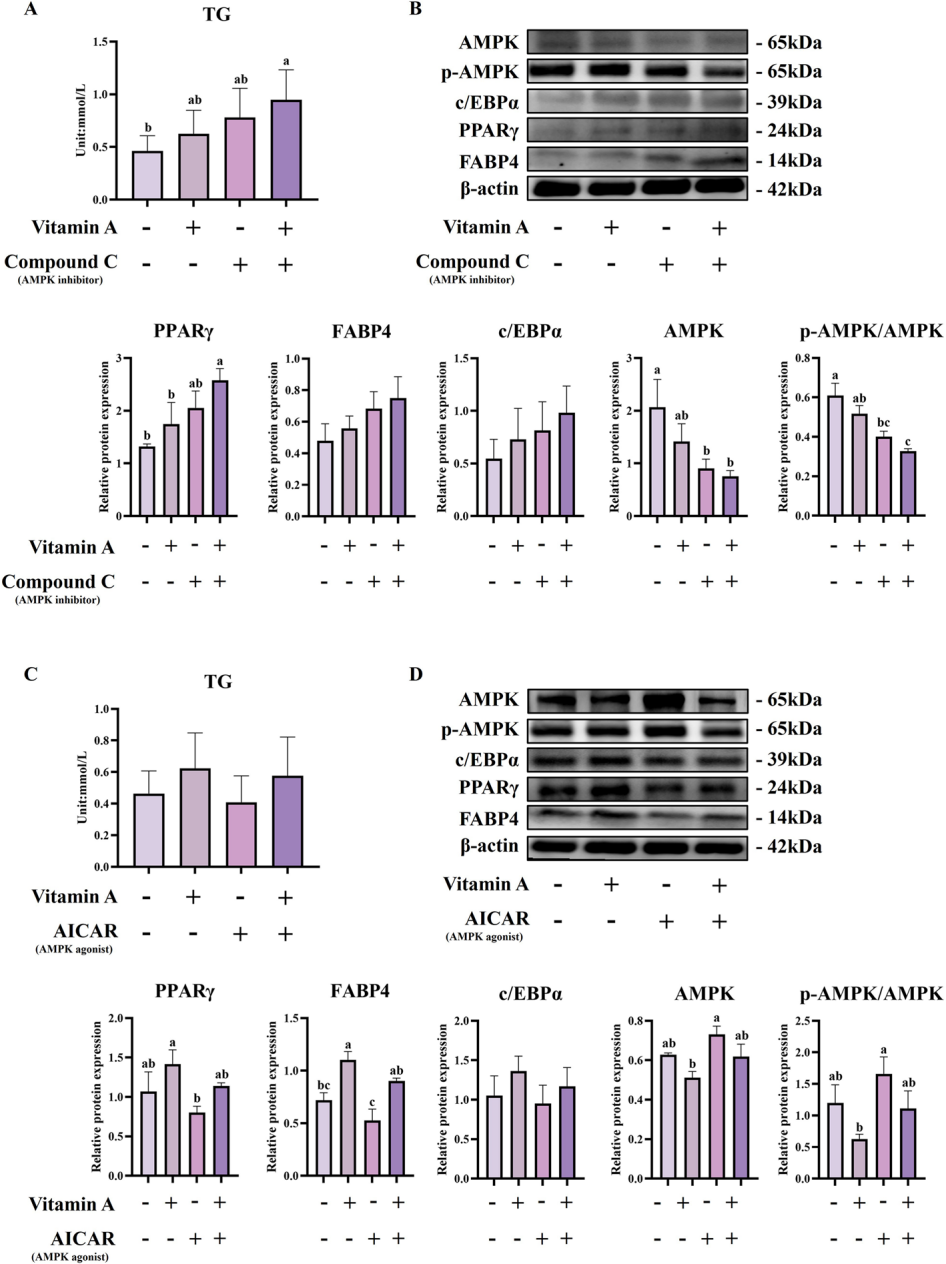
Pharmacological modulation of AMPK activity reveals its exclusive mediating role in vitamin A-Induced adipogenesis in BSMCs of boking black cattle. **A** Triglyceride (TG) concentrations and the expression levels of AMPK, p-AMPK, C/EBPα, PPARγ, and FABP4 proteins in adipocytes treated with control (CON), ATRA, Compound C, or ATRA + Compound C. **B** Quantification of relative protein expression levels shown in (**A**). **C** TG concentrations and protein expression levels in adipocytes treated with CON, ATRA, AICAR, or ATRA + AICAR. **D** Quantification of relative protein expression levels shown in (**C**). Data are presented as means ± standard deviation. ^a–c^Different lowercase indicate significant differences (*P* < 0.05)

To investigate the functional significance of AMPK activation during enhanced adipogenesis, we employed pharmacological modulation using AMPK inhibitor Compound C and agonist AICAR. AMPK inhibition with Compound C dramatically enhanced vitamin A-induced adipogenesis (Fig. 8A and B). The combined ATRA + Compound C treatment showed the highest triglyceride content, significantly exceeding control levels (*P* < 0.05). Western blot analysis confirmed that Compound C significantly reduced AMPK and p-AMPK expression (*P* < 0.05), while PPARγ expression increased progressively from vitamin A alone to combined treatment, reaching peak levels in the ATRA + Compound C group (*P* < 0.05). Conversely, AMPK activation with AICAR suppressed vitamin A-induced adipogenesis (Fig. 8C and D). While vitamin A alone enhanced adipogenesis, AICAR treatment abolished this effect, with both AICAR and ATRA + AICAR groups showing no significant increases in triglyceride levels compared to controls. AICAR treatment significantly increased p-AMPK levels (*P* < 0.05) while suppressing expression of adipogenic transcription factors. PPARγ and FABP4 expression were significantly lower in AICAR and ATRA + AICAR groups compared to vitamin A-only treatment (*P* < 0.05), while C/EBPα showed similar suppression patterns.

These results demonstrate that AMPK functions as a negative feedback regulator during vitamin A-mediated adipogenesis, consistent with previous studies showing AMPK-mediated inhibition of PPARγ activity and adipocyte differentiation [35, 36]. The enhanced adipogenesis following AMPK inhibition and suppressed lipogenesis following AMPK activation reveal that vitamin A-induced AMPK activation represents a homeostatic mechanism to prevent excessive lipid accumulation. This regulatory circuit suggests that vitamin A promotes adipogenesis while simultaneously activating AMPK as a metabolic brake, creating a balanced system for controlled lipid accumulation during late-stage adipogenic differentiation.

## Discussion

The current approach in premium beef production relies on vitamin A restriction throughout the entire fattening period to enhance intramuscular fat (IMF) deposition, based on the established negative correlation between serum vitamin A levels and marbling grade [5]. However, this conventional strategy often compromises nutritional quality and feeding efficiency due to prolonged nutrient restriction. Our study fundamentally challenges this approach by demonstrating that strategic vitamin A supplementation during late fattening not only enhances IMF deposition but also improves fatty acid composition through a sophisticated molecular mechanism where PPARγ signaling overrides AMPK-mediated metabolic constraints.

Our findings reveal that the relationship between vitamin A and marbling development is far more complex than previously understood, with timing being the critical determinant of outcome. The large-scale validation using 336 genetically homogeneous Woking black cattle (TT genotype of *ADH1C* gene) provided unprecedented evidence that vitamin A supplementation during late fattening creates a metabolic environment where serum vitamin A depletion correlates with superior marbling grades. This inverse correlation emerges only after supplementation, indicating active vitamin A utilization rather than simple restriction. The progressive decline in serum vitamin A from A1 to A4 grades demonstrates that superior marbling development requires adequate vitamin A provision followed by intensive consumption during lipogenesis, with cattle maintaining serum concentrations between 70–90 IU/dL showing optimal marbling development.

At the molecular level, our integrated analysis reveals a remarkable paradox where vitamin A supplementation simultaneously activates both lipogenic and anti-lipogenic pathways, with PPARγ signaling ultimately dominating over AMPK-mediated metabolic constraints. Both in vivo marbling grade comparisons and in vitro BSMC experiments consistently demonstrated that vitamin A treatment upregulated PPARγ expression while paradoxically increasing AMPK phosphorylation. This unexpected co-activation suggests that AMPK functions as a metabolic sensor responding to enhanced energy demands rather than acting as a simple inhibitor of fat synthesis [28, 29]. The transcriptomic analysis provided crucial mechanistic insights into this apparent contradiction. The increased p-AMPK levels in A4-grade cattle and vitamin A-treated cells represent a compensatory response to intensive lipogenic activity, functioning as a negative feedback mechanism to prevent excessive fat accumulation [37, 38]. However, the functional validation using AMPK modulators revealed that this endogenous AMPK activation was insufficient to override the dominant PPARγ-driven lipogenic program. AMPK inhibition with Compound C dramatically enhanced vitamin A-induced adipogenesis, while AMPK activation with AICAR completely abolished the lipogenic effects of vitamin A, confirming that AMPK acts as a metabolic brake that vitamin A-activated PPARγ signaling successfully overcomes.

Central to this mechanism is PPARγ functioning as the dominant transcriptional regulator of terminal adipogenesis. In both A4-grade cattle and vitamin A-treated BSMCs, PPARγ upregulation drove coordinated expression of downstream targets including FABP4, facilitating fatty acid transport and lipid droplet formation [23, 39]. Despite concurrent AMPK activation, the lipogenic machinery including ACACA, SREBP1, and FASN remained highly active, indicating that PPARγ-mediated transcriptional programs can override AMPK-dependent metabolic inhibition under appropriate conditions [34, 40].

The observed improvements in fatty acid composition including elevated EPA, DHA, and beneficial n-3 fatty acids, which primarily reflect enhanced fatty acid desaturation and incorporation into triglycerides during terminal adipogenesis. These compositional changes are consistent with PPARγ-mediated regulation of desaturase expression and preferential uptake of polyunsaturated fatty acids during the lipid-accumulation phase of adipocyte development. Vitamin A supplementation significantly increased beneficial fatty acids including EPA, DHA, and conjugated linoleic acids while elevating the PUFA/SFA ratio. The enhanced expression of SREBP1 and downstream desaturases likely contributed to this improved fatty acid composition [41, 42], while PPARγ activation facilitated PUFA uptake and stabilization in muscle tissue [43]. These compositional improvements represent a dual benefit that increased marbling grade coupled with enhanced nutritional value.

The stage-dependent vitamin A effects resolves longstanding contradictions in the literature regarding retinoic acid and adipogenesis. While early-stage vitamin A exposure inhibits preadipocyte differentiation [21], our data demonstrate that late-stage supplementation promotes terminal differentiation and lipid accumulation [22]. During the in vivo study, vitamin A supplementation (24–30 months) occurs in a systemic metabolic environment involving hormonal and nutritional interactions, whereas the in vitro study applied ATRA during D7–D11 of BSMC differentiation. Consequently, the direction or magnitude of certain markers may differ. This temporal specificity reflects the different cellular requirements and signaling environments present during early commitment versus terminal maturation phases of adipogenesis. Our findings have significant implications for premium beef production strategies. Rather than universal vitamin A restriction throughout fattening, we propose a precision nutrition approach combining early-to-mid fattening restriction with strategic late-stage supplementation. This approach accommodates the dynamic demands of adipogenic signaling pathways while optimizing both marbling development and meat quality. The demonstration that vitamin A supplementation during late fattening enhances rather than inhibits IMF deposition challenges fundamental assumptions in the beef industry and opens new avenues for marbling enhancement without compromising animal welfare or feeding efficiency.

Our future investigations will focus on several directions to further validate and expand these findings. First, optimizing vitamin A dosage and timing protocols across different cattle breeds and production systems will be essential to establish broader applicability of this approach. While the current study demonstrates significant improvements in marbling quality, future work should incorporate comprehensive production metrics including feed efficiency and growth performance to evaluate the overall economic benefits of late-stage vitamin A supplementation. Additionally, although serum glucose and LDL-C showed modest increases within normal physiological ranges, likely reflecting metabolic adaptations to enhanced lipogenesis, monitoring these parameters alongside welfare indicators in larger-scale trials would provide valuable insights into the systemic effects of this nutritional strategy. From a mechanistic perspective, several questions remain. Elucidating how PPARγ signaling overcomes AMPK-mediated metabolic constraints and exploring the potential roles of other nuclear receptors including VDR in coordinating vitamin A-responsive lipogenic programs remain important research priorities [27, 44]. Additionally, while our study focused on lipogenic pathways during late-stage adipogenesis where triglyceride synthesis dominates, a more complete understanding the metabolic effects of vitamin A would benefit from assessment of lipolytic and fatty acid oxidation pathways across different developmental stages. Understanding the temporal dynamics of metabolic switching during different fattening phases will ultimately enable development of more precise nutritional interventions for maximizing intramuscular fat deposition while maintaining quality in premium beef production systems.

## Conclusions

This study demonstrated that strategic vitamin A supplementation (3,000 IU/kg DM) during late fattening significantly enhances intramuscular fat deposition in Woking black cattle, achieving ≥ A3 marbling grades in 75% of individuals while improving fatty acid composition with elevated EPA, DHA, and beneficial n-3 fatty acids in higher-grade beef. Under supplementation conditions, serum vitamin A concentrations showed inverse correlation with marbling grades (optimal window: 70–90 IU/dL), indicating active utilization rather than restriction drives superior marbling. The PPARγ-AMPK regulatory axis was identified as the central mechanism, where vitamin A supplementation paradoxically activates both PPARγ-driven lipogenic pathways and AMPK phosphorylation, with the dominant PPARγ signaling ultimately overriding AMPK-mediated metabolic constraints to promote intramuscular fat accumulation. These findings establish a novel nutritional paradigm for premium beef production that challenges conventional vitamin A restriction practices, offering dual benefits of enhanced marbling quality and improved nutritional value through temporally targeted supplementation.

## Supplementary Information

Additional file 1: Fig. S1. Agarose gel electrophoresis of the ADH1C c.-64T>C PCRRFLP. Table S1. Gene information and polymerase chain reactionprimer sequences. Table S2. Antibody information and dilutions. Table S3. RNA-seq Quality Metrics.

Additional file 2. Differentially expressed genes identified by RNA-seq.

Additional file 3. Original gels of the Western blots.

## Data Availability

No datasets were generated or analysed during the current study.

## Abbreviations

ACACA: Acetyl-CoA carboxylase
ADH1C: Alcohol dehydrogenase 1C
AICAR: 5-Aminoimidazole-4-carboxamide ribonucleotide
AKT: Protein kinase B
AMPK: AMP-activated protein kinase
ATRA: All-*trans*-retinoic acid
BSMC: Bovine skeletal muscle-derived cells
c/EBPα: CCAAT/enhancer-binding protein alpha
CFL2: Cofilin 2
CLA: Conjugated linoleic acid
CPNE4: Copine IV
DEPC: Diethyl pyrocarbonate
DHA: Docosahexaenoic acid
DMEM: Dulbecco’s modified Eagle’s medium
DMSO: Dimethyl sulfoxide
ECL: Enhanced chemiluminescence
EPA: Eicosapentaenoic acid
FABP4: Fatty acid-binding protein 4
FASN: Fatty acid synthase
FBS: Fetal bovine serum
FPKM: Fragments per kilobase million
GC: Gas chromatography
GLU: Glucose
GPT: Glutamic pyruvic transaminase
IACUC: Institutional Animal Care and Use Committee
IMF: Intramuscular fat
LDL-C: Low-density lipoprotein cholesterol
mTOR: Mammalian target of rapamycin
NEFA: Non-esterified fatty acids
NRC: National Research Council
P/S: Penicillin-streptomycin
PMSF: Phenylmethylsulfonyl fluoride
PPARγ: Peroxisome proliferator-activated receptor gamma
PVDF: Polyvinylidene fluoride
RA: Retinoic acid
RAR: Retinoic acid receptor
RNA-seq: RNA sequencing
RIPA: Radioimmunoprecipitation assay buffer
RXR: Retinoid X receptor
SCD: Stearoyl-CoA desaturase
SDS-PAGE: Sodium dodecyl sulfate–polyacrylamide gel electrophoresis
SREBP1: Sterol regulatory element-binding protein 1
TG: Triglyceride
TSC2: Tuberous sclerosis complex 2
VA: Vitamin A
VDR: Vitamin D receptor
