# Supplementary material for: Vitamin A-activated PPARγ signaling enhances intramuscular fat accumulation by overriding AMPK-mediated inhibition in late-fattening beef cattle
Source: J Anim Sci Biotechnol. 2026 Feb 12;17:29. doi: 10.1186/s40104-025-01343-1 (PMC12896250; doi:10.1186/s40104-025-01343-1)
Supplement: Supplementary file 1 — Additional file 1: Fig. S1. Agarose gel electrophoresis of the ADH1C c.-64T>C PCRRFLP. Table S1. Gene information and polymerase chain reactionprimer sequences. Table S2. Antibody information and dilutions. Table S3. RNA-seq Quality Metrics. [file 40104_2025_1343_MOESM1_ESM.docx]

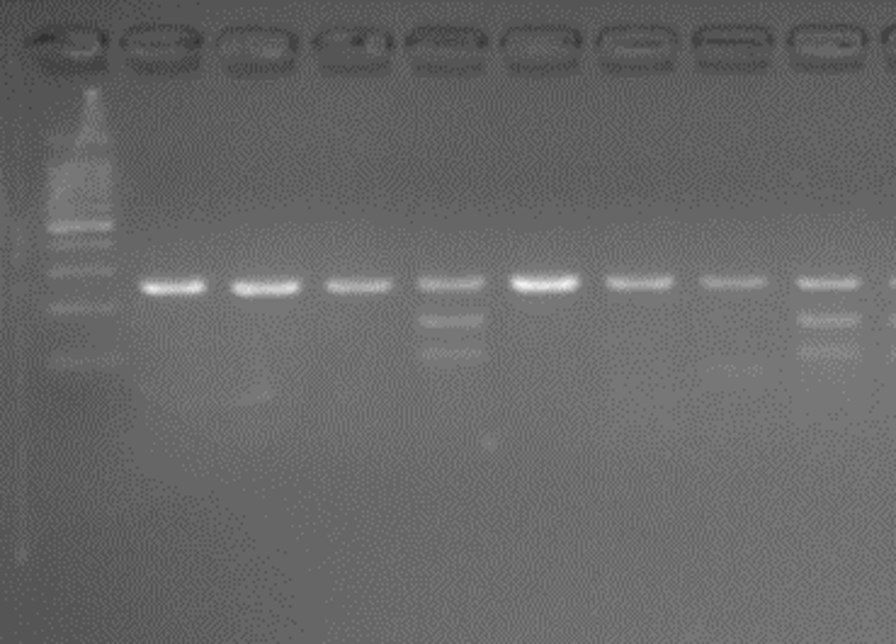


**Supplementary Figure 1.** Agarose gel electrophoresis of the ADH1C c.-64T>C PCRRFLP. Lane 1 is 100bp DNA Ladder Marker, Lane 2 is TT control, Lane 4 is TC control.

**Supplementary Table 1.** Gene information and polymerase chain reaction (PCR) primer sequences.

| Target gene | Forward primer (5’→3’) | Reverse primer (5’→3’) | Accession number | Annealing temperature, ℃ |
| --- | --- | --- | --- | --- |
| PPARγ | GCTCCAAGAGTACCAAAGTGCAA | CCTGACGCTTTATCCCCACA | NM_181024.2 | 63.4 |
| C/EBPα | TGGAGCTGACCAGTGACAAT | AGTTCGCGGCTCAGTTGTTC | NM_176784.2 | 63.4 |
| FABP4 | TGACAGGAAAGTCAAGAGCATCGT | GGTGGTTGATTTTCCATCCCAGT | NM_174314.2 | 60.6 |
| CFL2 | AGCAGATCTTGGTGGGTGAC | TGTGGCATCGTACAAAGCAT | NM_001076154.2 | 62.3 |
| Villin 2 | GGCTCCTGACTTCGTGTTCT | ATCTGCTGCACCTCAATGGT | NM_174217.2 | 63.4 |
| CAPZA2 | CCGAATGGAGTCTGCACTGT | CACTCTGACCTCCAACGACC | NM_001012998.2 | 62.3 |
| *RAR* | CACCAGCAGAGGTATCTCAGTAAC | CTTCTCCTGAAAAAGCCCTTGC | NM_001192861.1 | 60.2 |
| *RXRA* | AACACAAGTACCCCGAGCAG | GTGTCCCCGATGAGCTTGAA | NM_001304343.1 | 60.0 |
| *VDR* | GCCAGCACTTCCTTACCTGA | TTCATGCTCCGCCTGAAGAA | NM_001167932.2 | 60.2 |
| HSP70 | GACATCAGCCAGAACAAGCG | AAGTCGATGCCCTCGAACAG | NM_203322.3 | 64.0 |
| SREBP1 | CGCTCTTCCATCAATGACA | TTCAGCGATTTGCTTTTGTG | NM_001113302.1 | 62.3 |
| *FASN* | GACCTGGGAGGAGTGTAAGC | GCGATAGCGTCCATGAAGTA | NM_001012669.1 | 62.3 |
| *SCD* | CCACGTTCTTCATTGATTGC | CAGCCACTCTTGTAGCTTTCC | NM_173959.4 | 60.0 |
| ACACA | GGAGACAAACAGGGACCATTAC | GTGGAAGGAATGCTTGGGAG | NM_174224.2 | 60.0 |
| RPS9 | CCGGAACAAACGTGAGGTCT | GCAACAGGGCATTACCTTCG | NM_001101152.2 | 63.4 |
| RPLP0 | CAACCCGGCTCTGGAGAAACTG | ACTTCACACGGCGCTATGG | NM_001012682.1 | 62.3 |
| EIF3K | CAAGGGGATCGACAGGTACAAC | AGGCATTTTCTTTGGCCTGTG | NM_001034489.2 | 62.3 |

PPARγ = peroxisome proliferator activated receptor gamma; c/EBPα = CCAAT enhancer binding protein alpha; FABP4 = fatty acid binding protein 4; CFL2 = cofilin 2; Villin 2 = villin 2; CAPZA2 = capping actin protein of muscle Z-line subunit alpha 2; RAR = retinoic acid receptor; RXRA = retinoid X receptor alpha; VDR = vitamin D receptor; HSP70 = heat shock protein 70; SREBP1 = sterol regulatory element binding protein 1; FASN = fatty acid synthase; SCD = stearoyl-CoA desaturase; ACACA = acetyl-CoA carboxylase alpha; RPS9 = ribosomal protein S9; RPLP0 = ribosomal protein lateral stalk subunit P0; EIF3K = eukaryotic translation initiation factor 3 subunit K.

**Supplementary Table 2.** Antibody information and dilutions.

| Antibodies Name | Diluted Multiples | Accession Number | Reagent Company |
| --- | --- | --- | --- |
| Mouse anti-β-actin polyclonal antibody | 1:5,000 | T0022 | Affinity |
| Rabbit anti-PPARγ polyclonal antibody | 1:5,000 | PAA886Bo01 | Cloud-clone |
| Rabbit anti-C/EBPα polyclonal antibody | 1:2,000 | bs-1630R | Bioss |
| Rabbit anti-FABP4 Polyclonal antibody | 1;5,000 | 12802-1-AP | Proteintech |
| Mouse anti-VDR Monoclonal antibody | 1:8,000 | 67192-1-Ig | Proteintech |
| Rabbit anti-HSP70 Polyclonal antibody | 1:5,000 | 10995-1-AP | Proteintech |
| Rabbit anti- FASN Polyclonal antibody/ | 1:5,000 | 10624-2-AP | Proteintech |
| Rabbit anti-ACC1 Polyclonal antibody/21923-1-AP | 1:1,000 | 21923-1-AP | Proteintech |
| Rabbit anti-SCD1 polyclonal antibody | 1:1,000 | bs-3787R | Bioss |
| Rabbit anti-REBP1 polyclonal antibody | 1:2,000 | bs-1402R | Bioss |
| Rabbit anti-AMPK alpha 1 polyclona antibody | 1:1,000 | AF6422 | Affinity |
| Rabbit anti-phospho AMPK alpha (Thr172) antibody | 1:1,000 | AF3423 | Affinity |
| Rabbit anti-PI3K p85 alpha polyclonal antibody | 1:2,000 | AF6241 | Affinity |
| Rabbit anti Phospho-PI3K p85 alpha polyclonal antibody | 1:2,000 | AF3241 | Affinity |
| Rabbit anti-Akt polyclonal antibody | 1:1000 | 10176-2-AP | Proteintech |
| Mouse anti-phospho-Akt (Ser473) monoclonal antibody | 1:1000 | bsm-33281M | Bioss |
| Rabbit anti-mTOR polyclonal antibody | 1:2000 | 28273-1-AP | Proteintech |
| Rabbit anti-phospho-mTOR polyclonal antibody | 1:2000 | 67778-1-lg | Proteintech |
| Goat anti-rabbit IgG antibody | 1:20,000 | bs-40295G-HRO | Bioss |
| Goat anti-mouse IgG antibody | 1:20,000 | bs-40296G-HRP | Bioss |

**Supplementary Table 3.** RNA-seq Quality Metrics

| Sample | Raw Reads | Total Clean Reads | GC Contents | Total Mapping Ratio | Uniquely Mapping Ratio |
| --- | --- | --- | --- | --- | --- |
| A1_01 | 47159560 | 46509518 | 51.36% | 98.17% | 94.63% |
| A1_02 | 43732988 | 43117226 | 51.09% | 98.40% | 94.73% |
| A1_03 | 41352182 | 40631644 | 50.86% | 98.22% | 94.71% |
| A1_04 | 44628218 | 43754272 | 51.18% | 98.21% | 94.65% |
| A1_05 | 42248248 | 41499100 | 51.70% | 98.38% | 94.59% |
| A1_06 | 45541538 | 44885190 | 51.30% | 97.93% | 94.28% |
| A1_07 | 41849474 | 41164594 | 50.79% | 98.27% | 94.92% |
| A1_08 | 40458448 | 39581256 | 51.85% | 97.92% | 94.03% |
| A1_09 | 43498794 | 43078672 | 52.19% | 98.20% | 94.47% |
| A1_10 | 44158962 | 43479138 | 50.96% | 97.93% | 94.49% |
| A4_01 | 46474828 | 45301582 | 52.37% | 98.11% | 94.38% |
| A4_02 | 40542764 | 36725770 | 51.09% | 97.81% | 94.33% |
| A4_03 | 48133870 | 45977638 | 51.60% | 97.60% | 93.59% |
| A4_04 | 59428500 | 57077248 | 51.07% | 98.11% | 94.19% |
| A4_05 | 55822828 | 54309330 | 50.86% | 97.76% | 94.17% |
| A4_06 | 56837002 | 54295362 | 51.55% | 98.34% | 94.56% |
| A4_07 | 56831798 | 54669614 | 51.54% | 98.20% | 94.43% |
| A4_08 | 50670522 | 48868614 | 51.11% | 97.34% | 93.75% |
| A4_09 | 46454690 | 45939356 | 47.43% | 98.27% | 95.63% |
| A4_10 | 62794434 | 60944634 | 50.28% | 98.19% | 94.78% |
