# Supplementary material for: Vitamin A-activated PPARγ signaling enhances intramuscular fat accumulation by overriding AMPK-mediated inhibition in late-fattening beef cattle
Source: J Anim Sci Biotechnol. 2026 Feb 12;17:29. doi: 10.1186/s40104-025-01343-1 (PMC12896250; doi:10.1186/s40104-025-01343-1)

**Figure 1(E).** Analysis of the expression of fat synthesis-related proteins in the longissimus dorsi muscle.

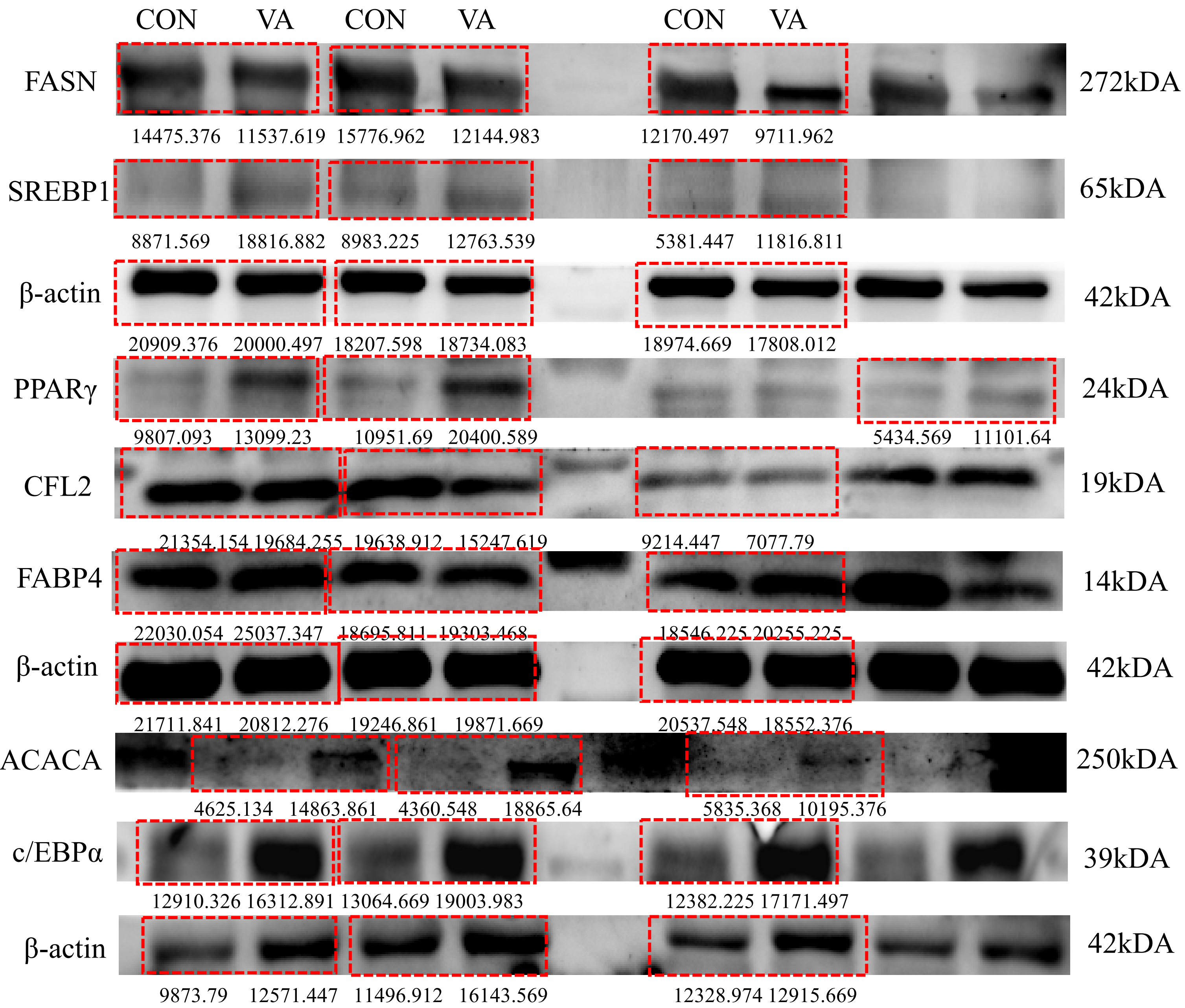

**FASN**

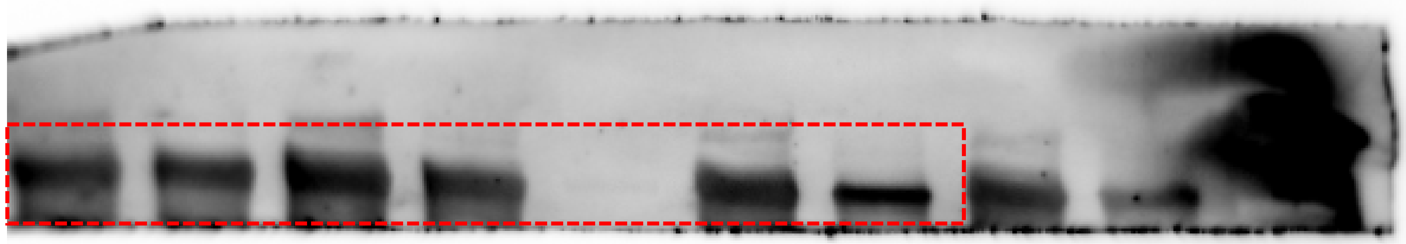

SREBP1

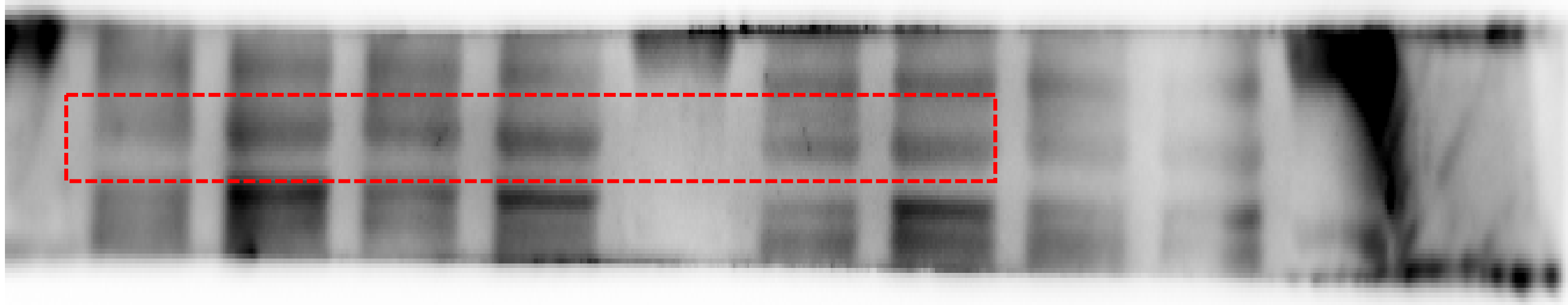

β-actin_1

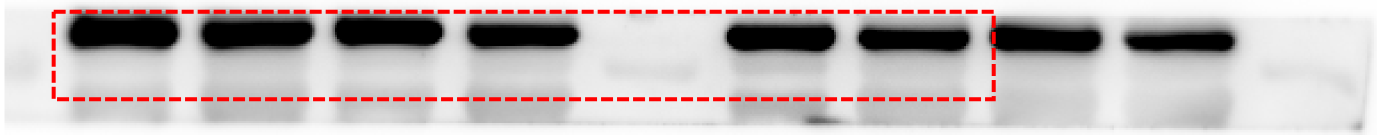

**PPARγ**

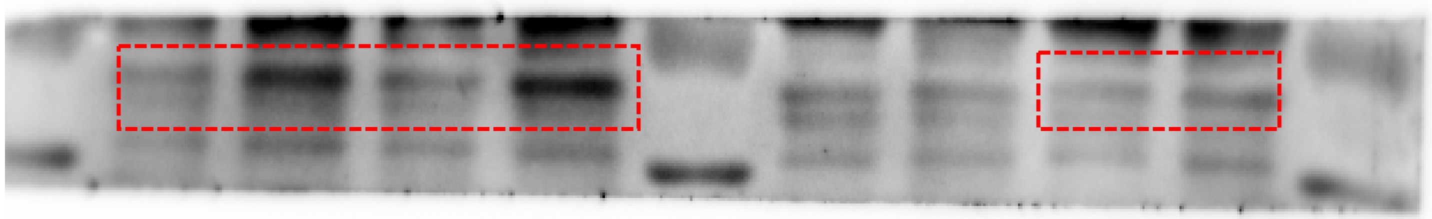

**CFL2**

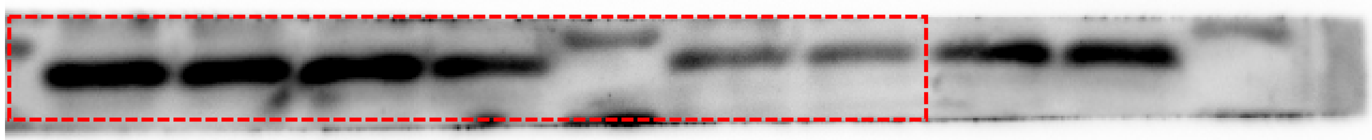

**FABP4**

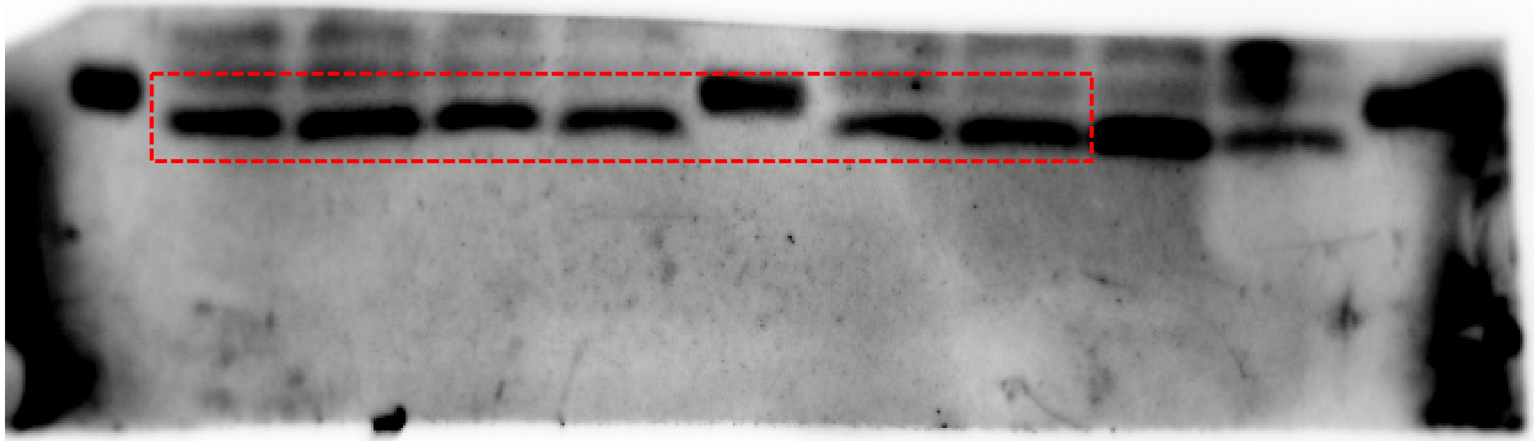

**β-actin_2**

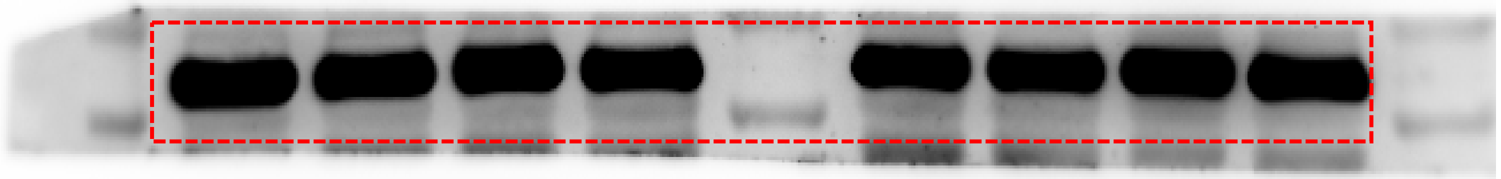

ACACA

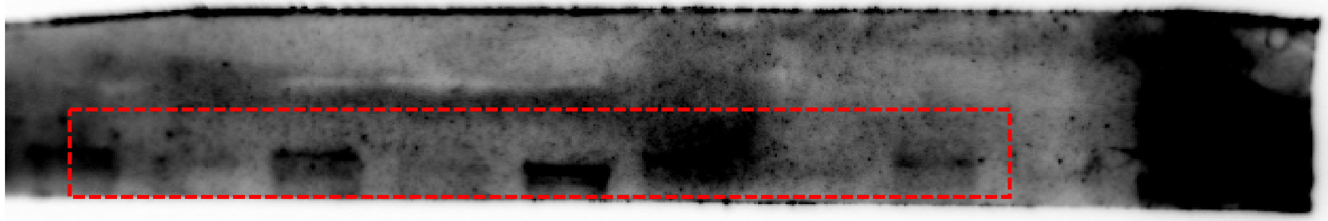

c/EBPα

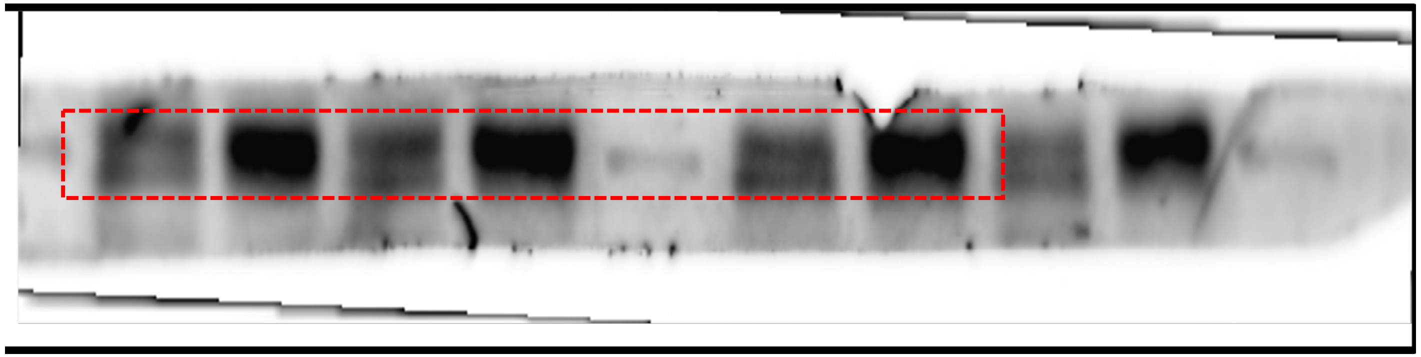

**β-actin_3**

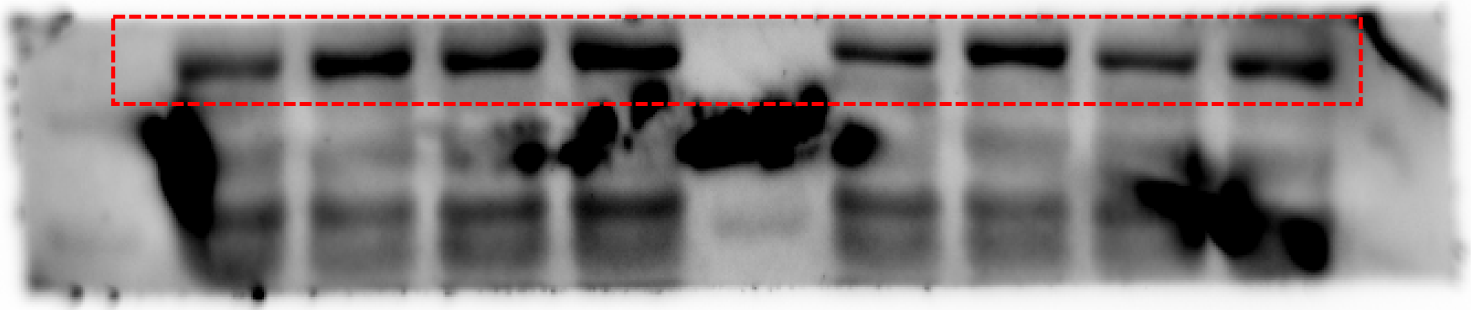

**Figure 2(D).** Expression of adipogenesis-related proteins in BSMCs of Woking black cattle.

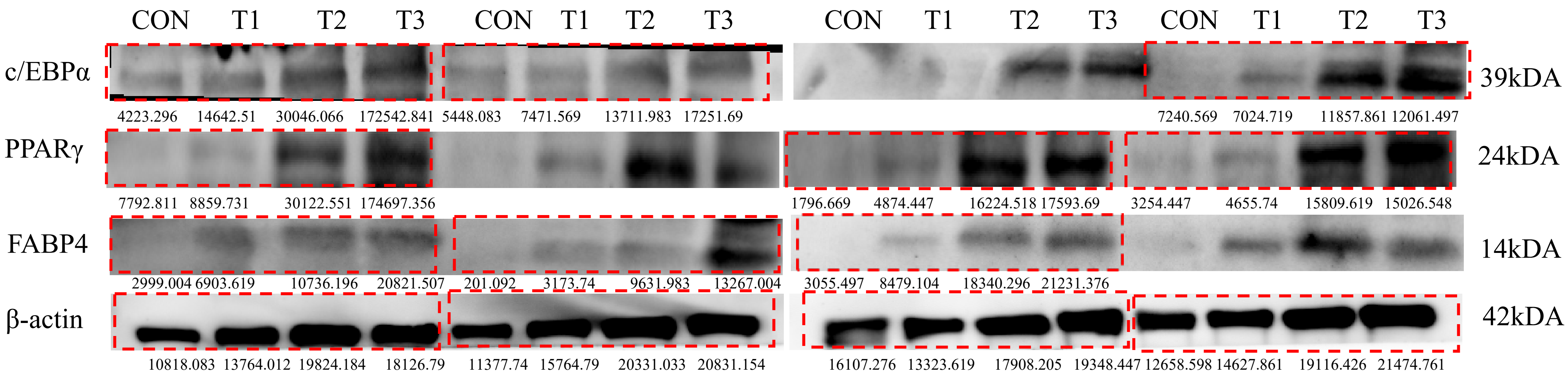

c/EBPα

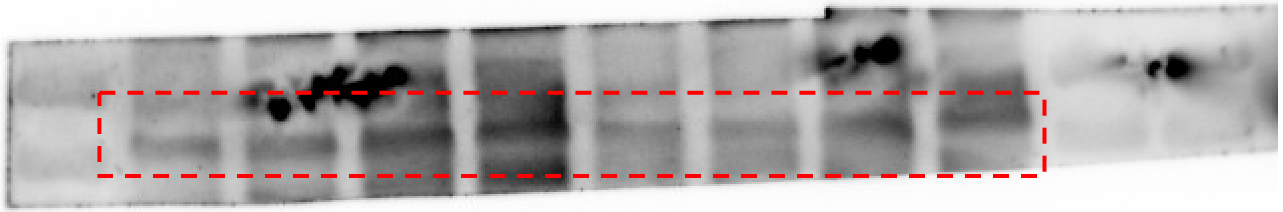

(1)

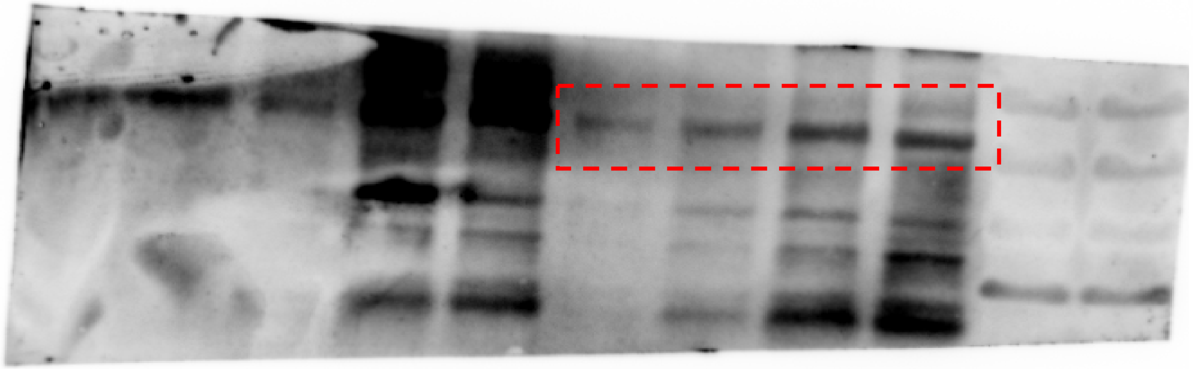

(2)

**PPARγ**

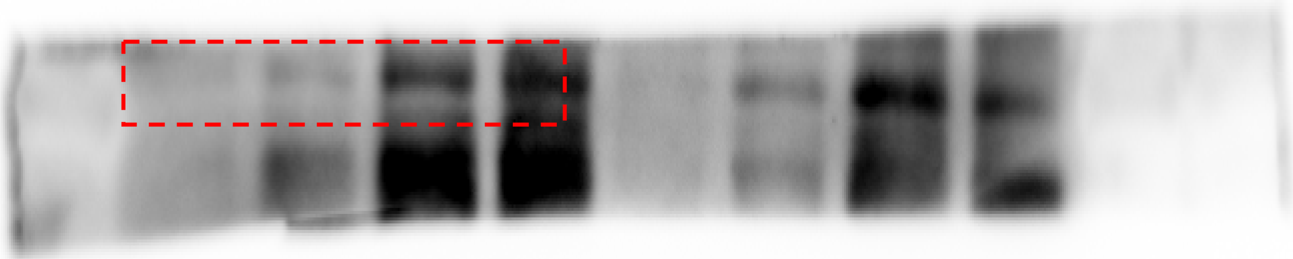

**(1)**

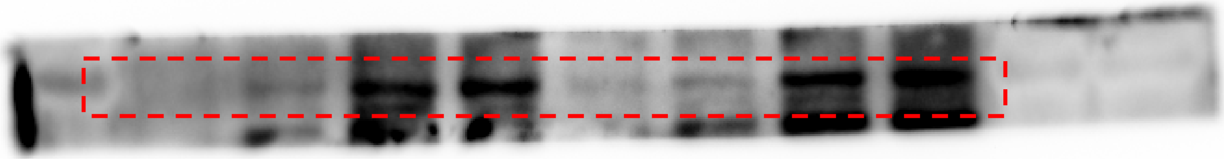

**(2)**

**FABP4**

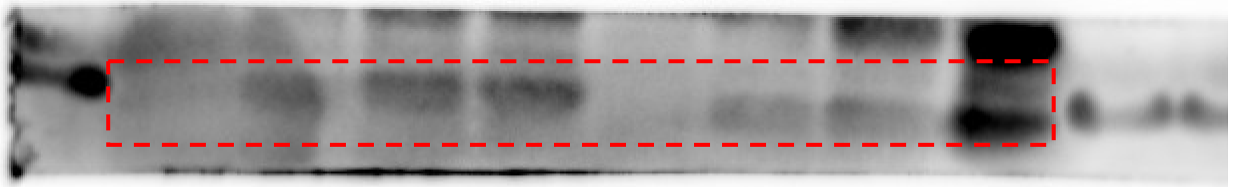

**(1)**

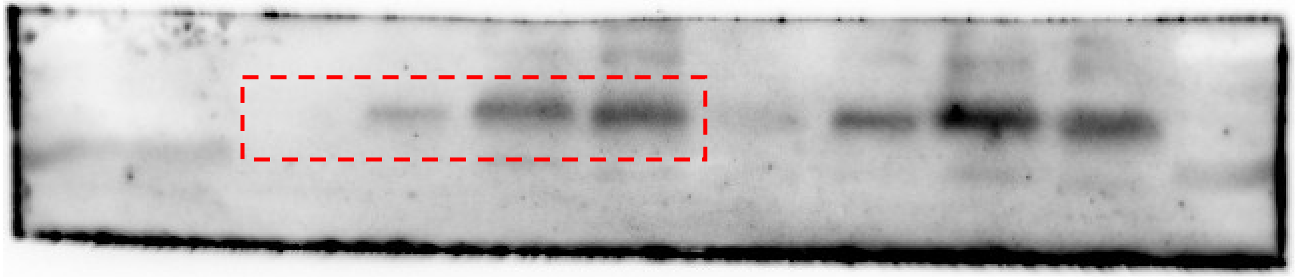

**(2)**

**β-actin**

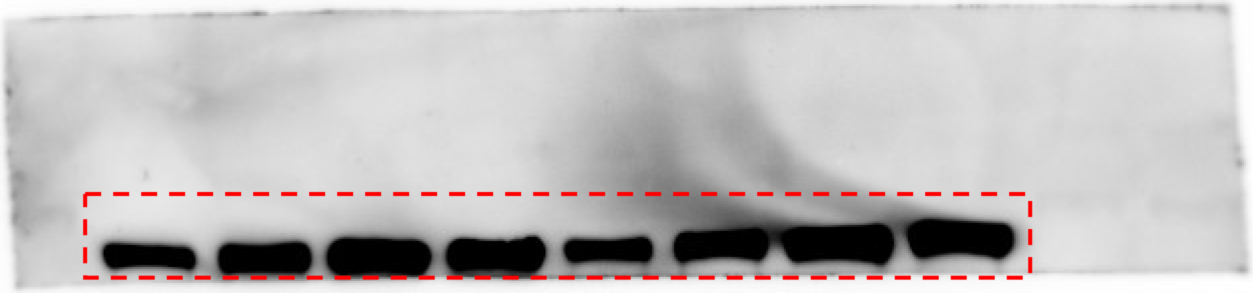

(1)

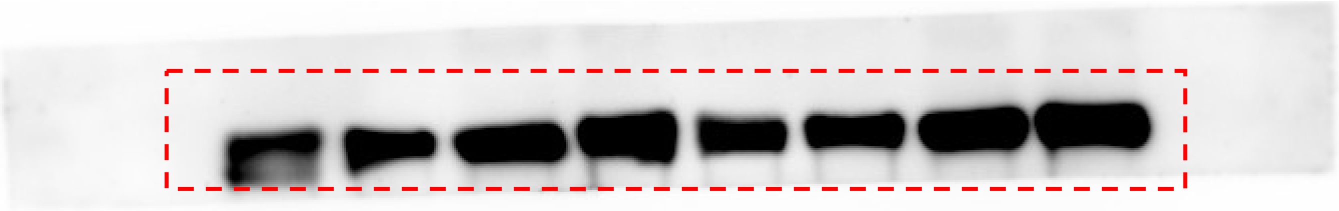

(2)

Figure 5(A). Vitamin A supplementation during late fattening period enhances adipogenic protein expression in Woking black cattle.

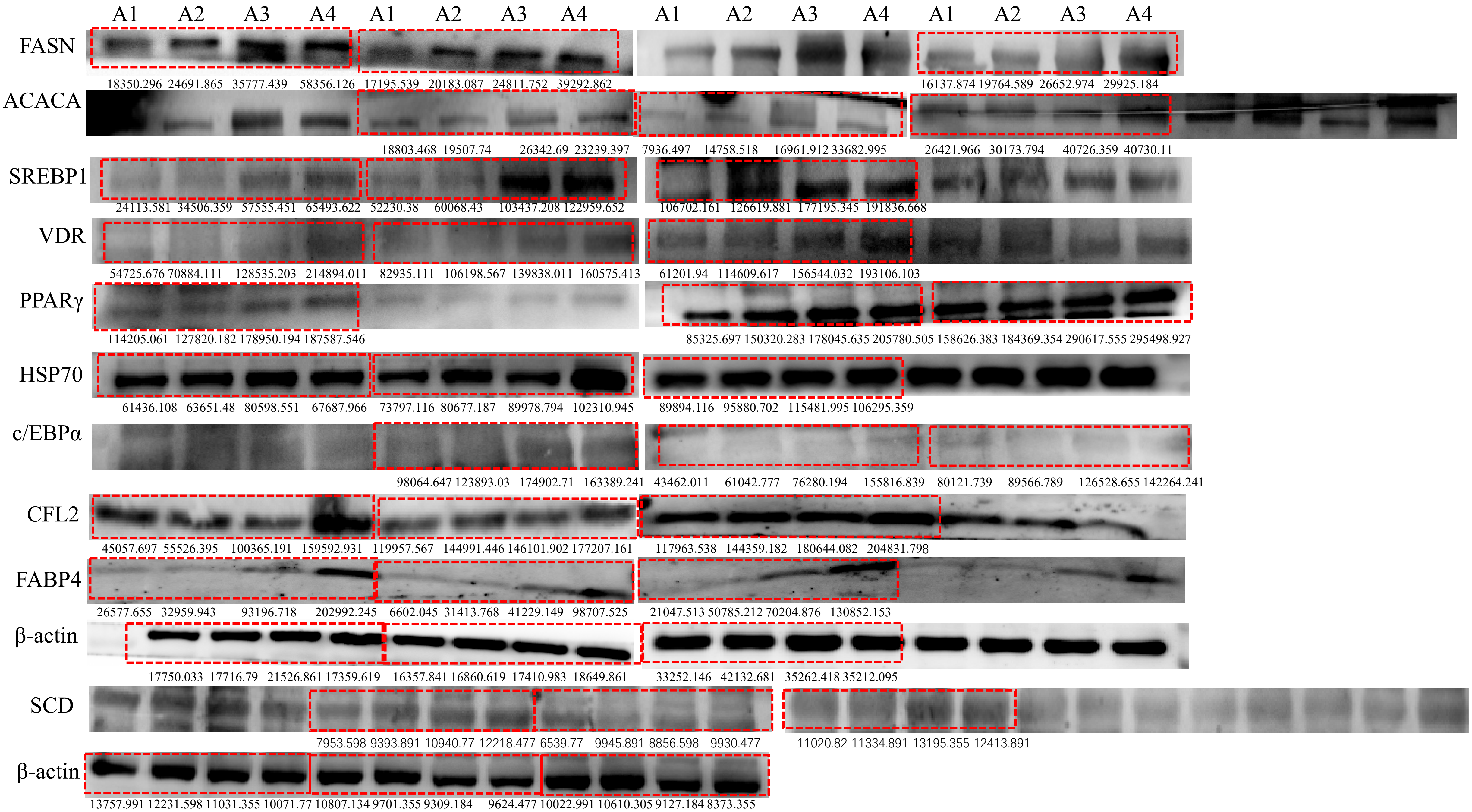

FASN

(1)

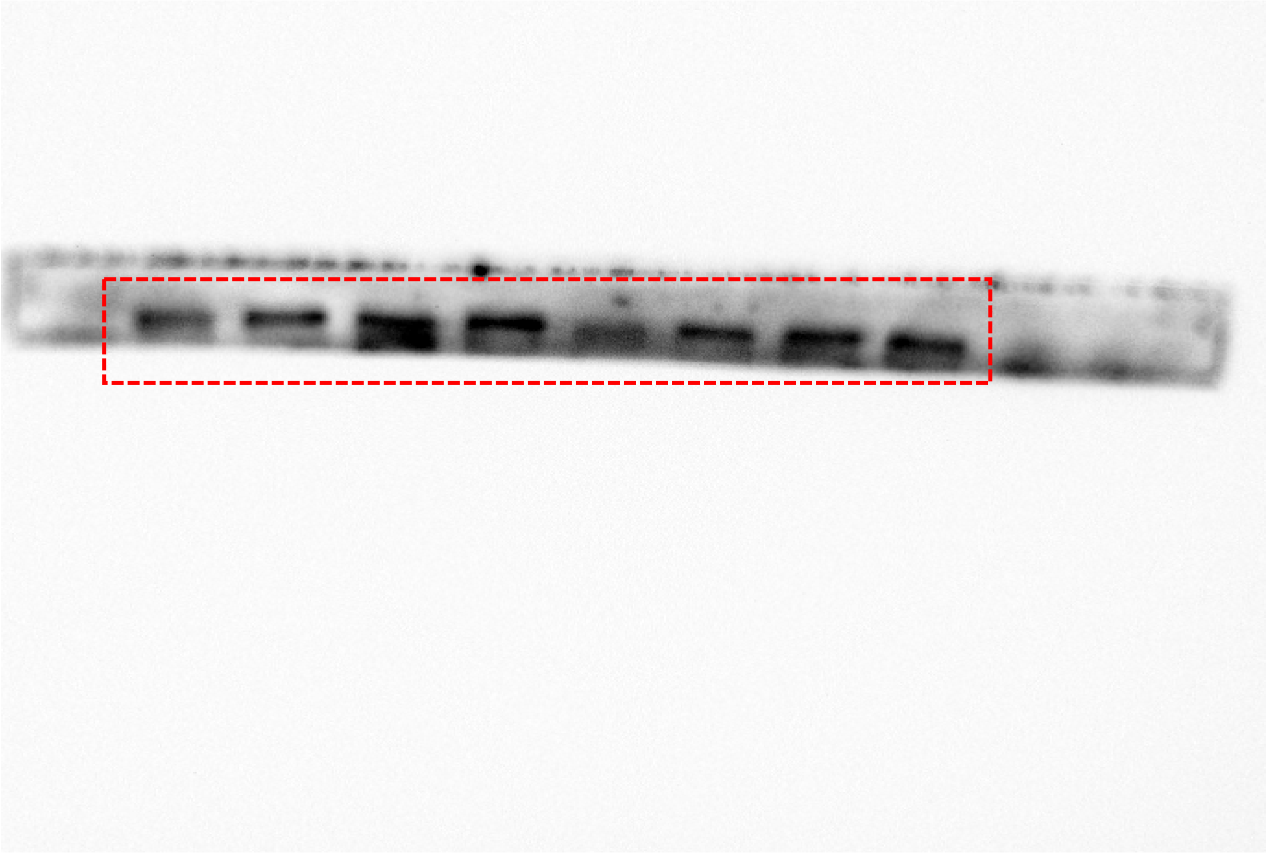

(2)

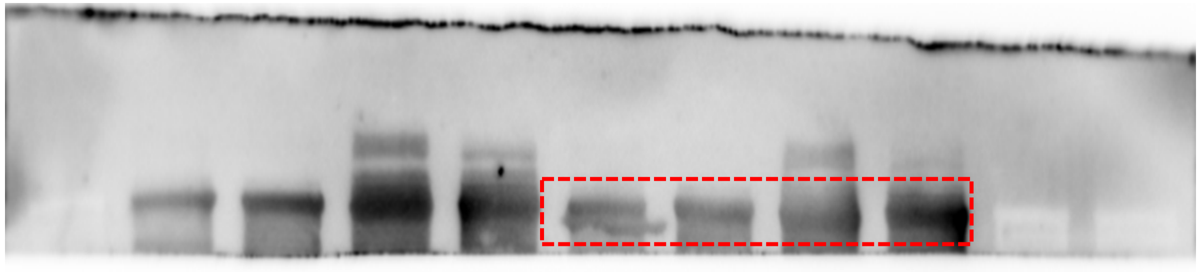

ACACA

(1)

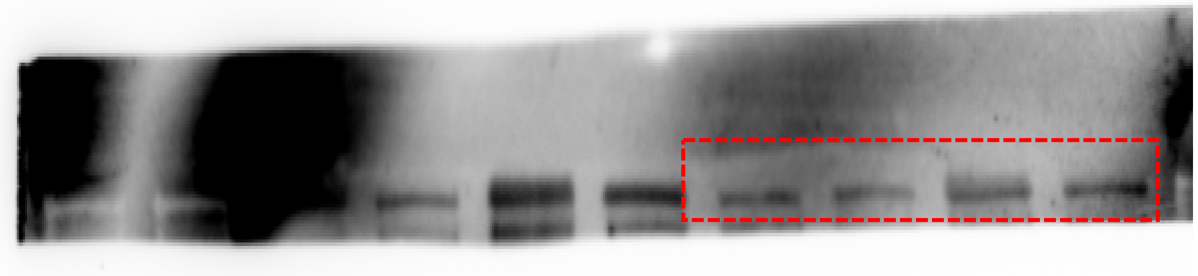

(2)

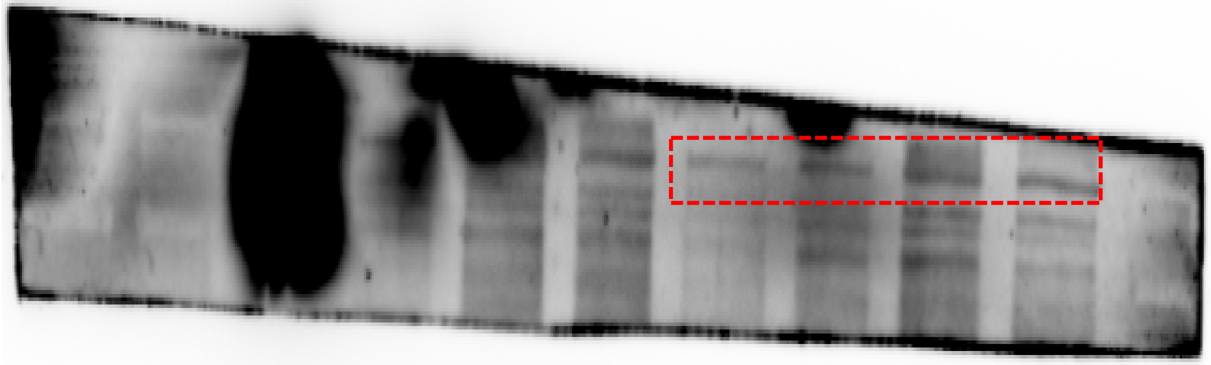

(3)

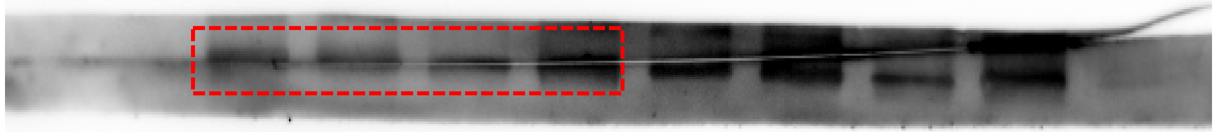

SREBP1

(1)

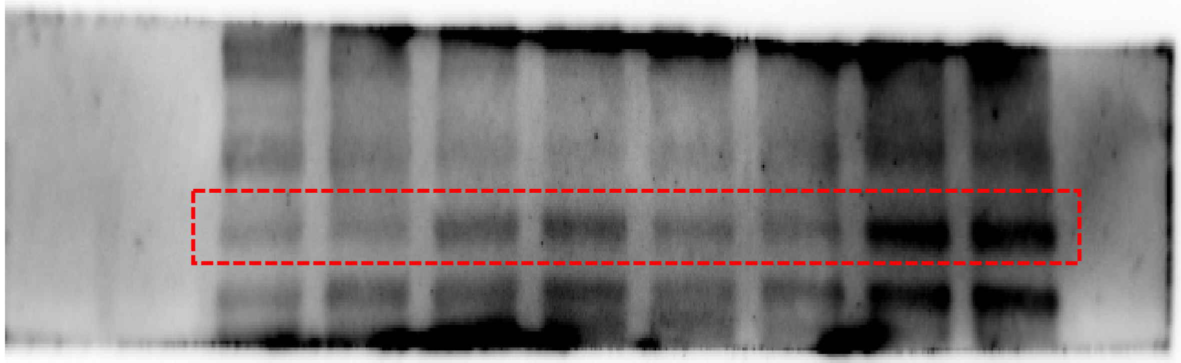

(2)

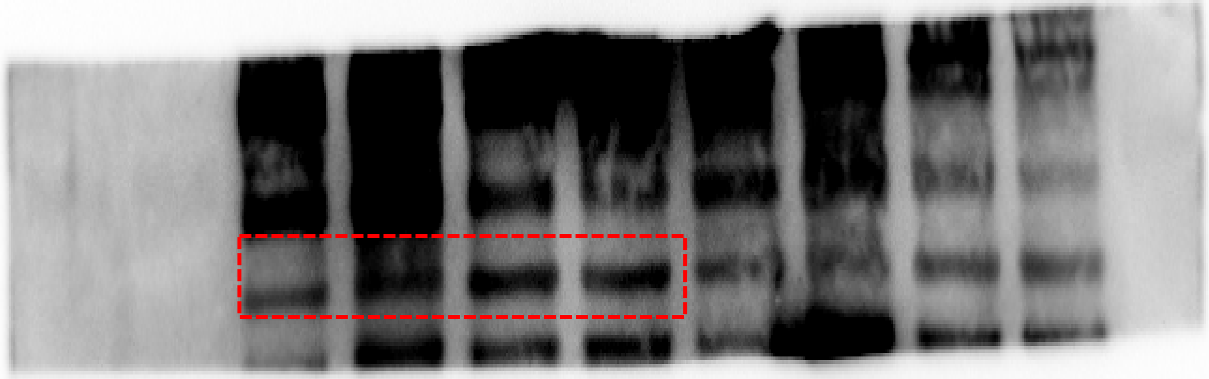

VDR

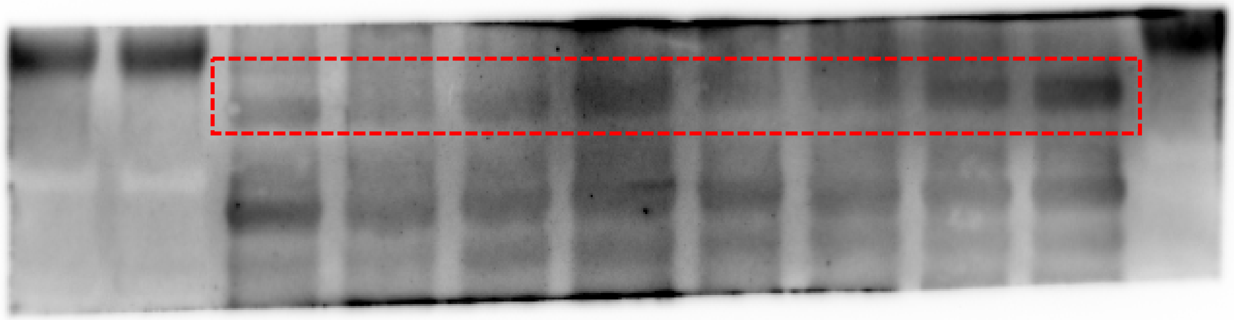

(1)

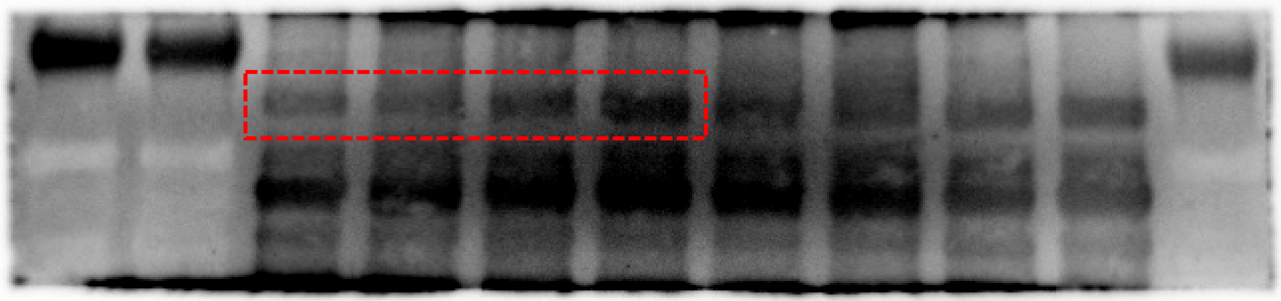

(2)

PPARγ

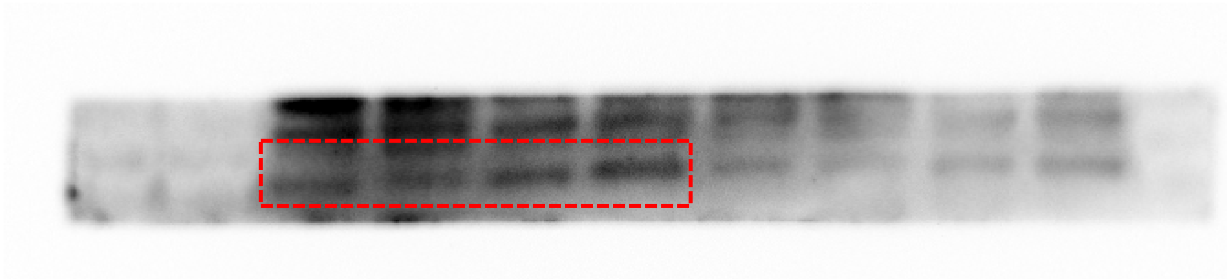

(1)

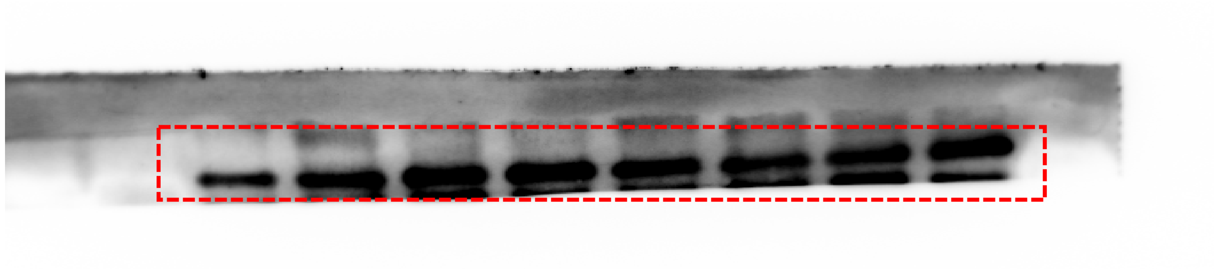

(2)

HSP70

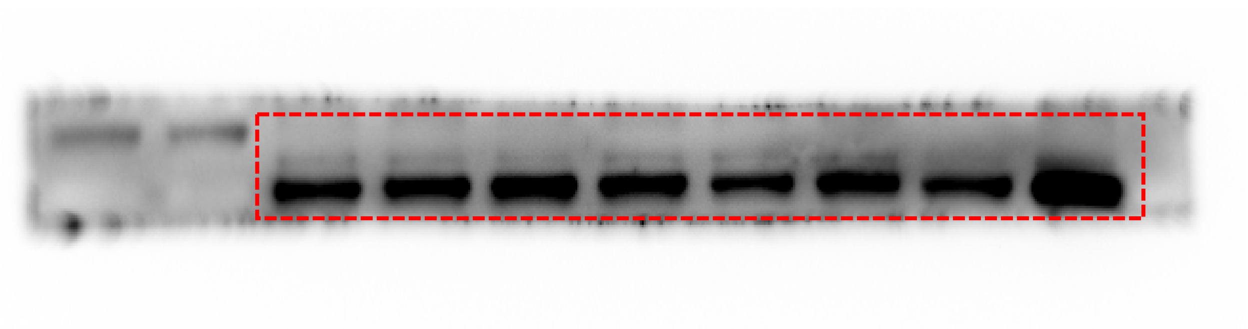

**(1)**

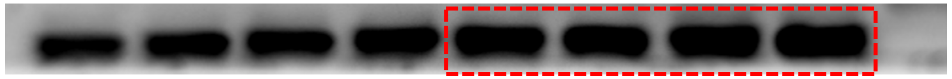

**(2)**

c/EBPα

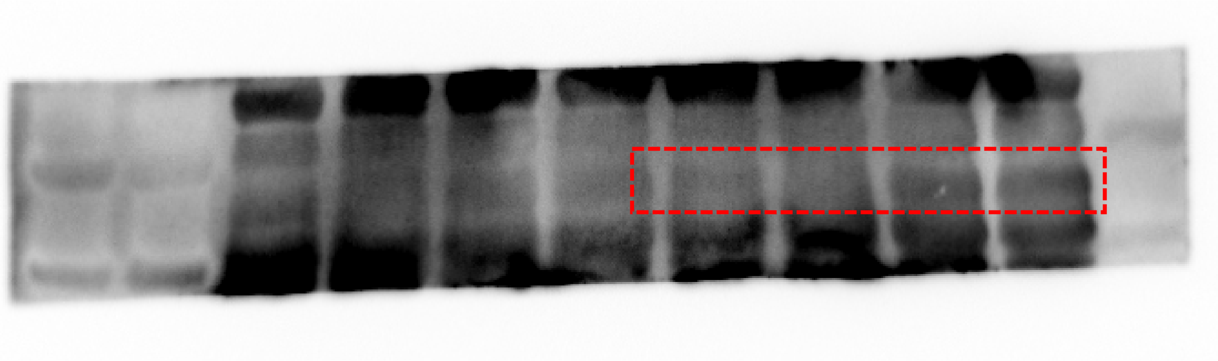

**(1)**

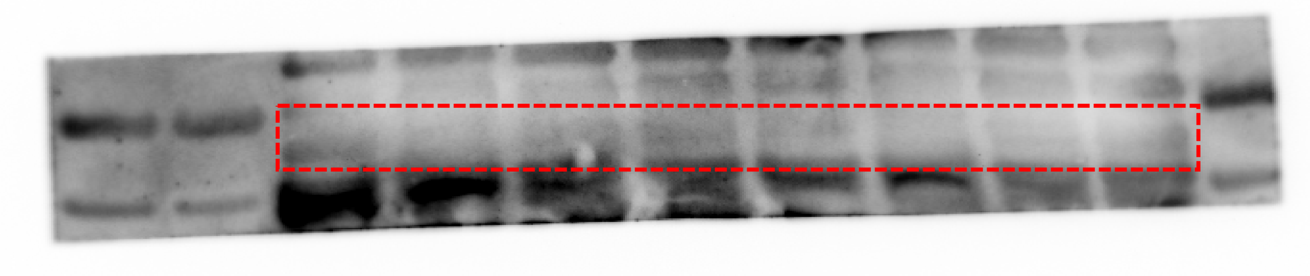

**(2)**

CFL2

(1)

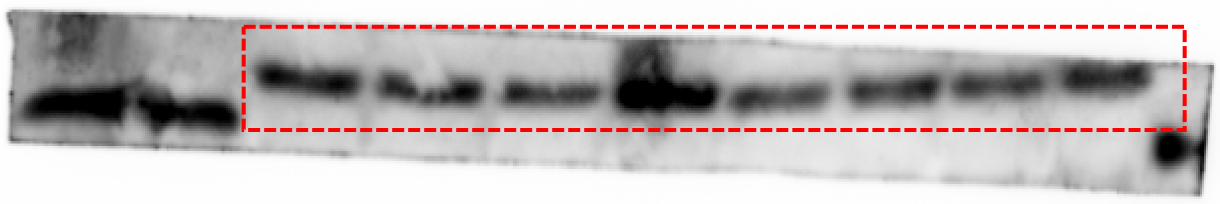

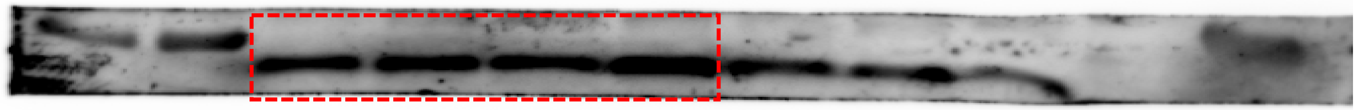

(2)

FABP4

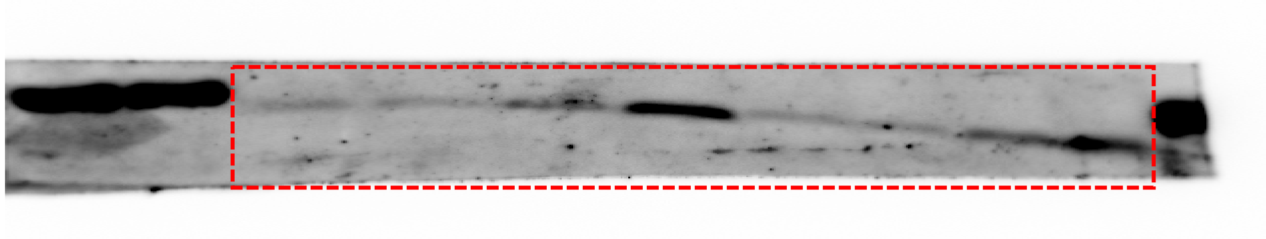

(1)

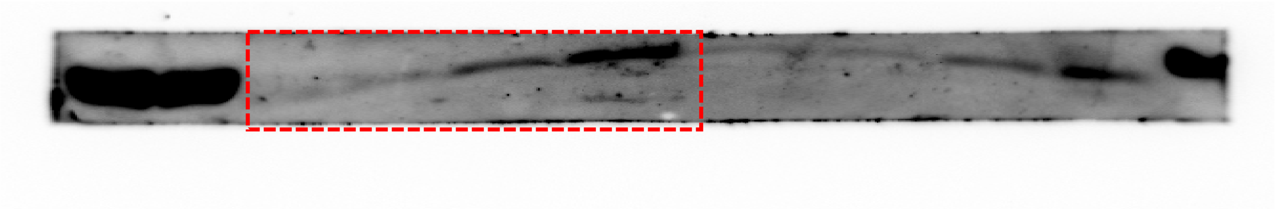

(2)

β-actin_1

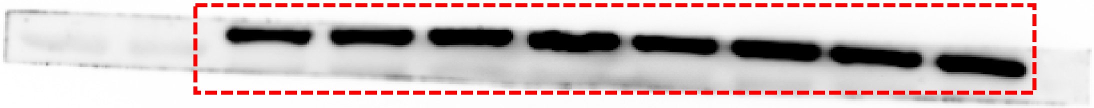

**(1)**

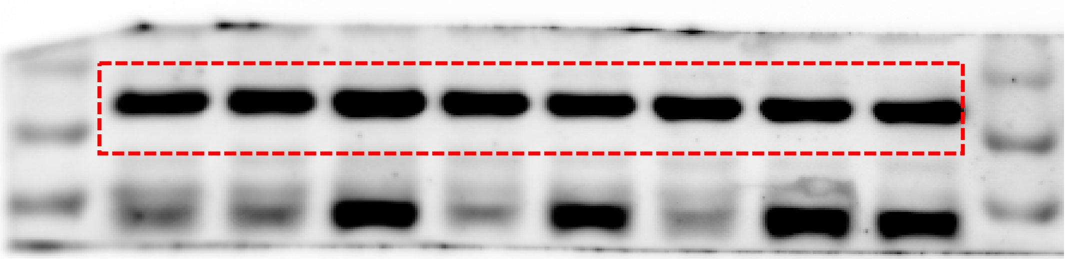

**(2)**

SCD

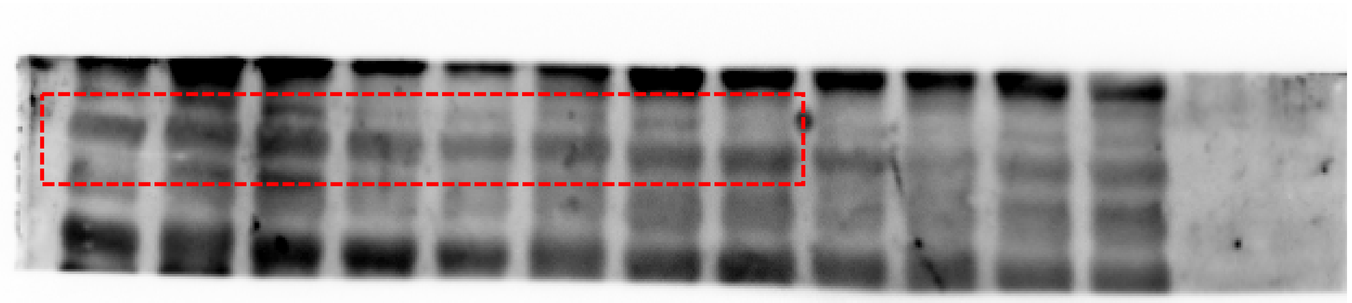

(1)

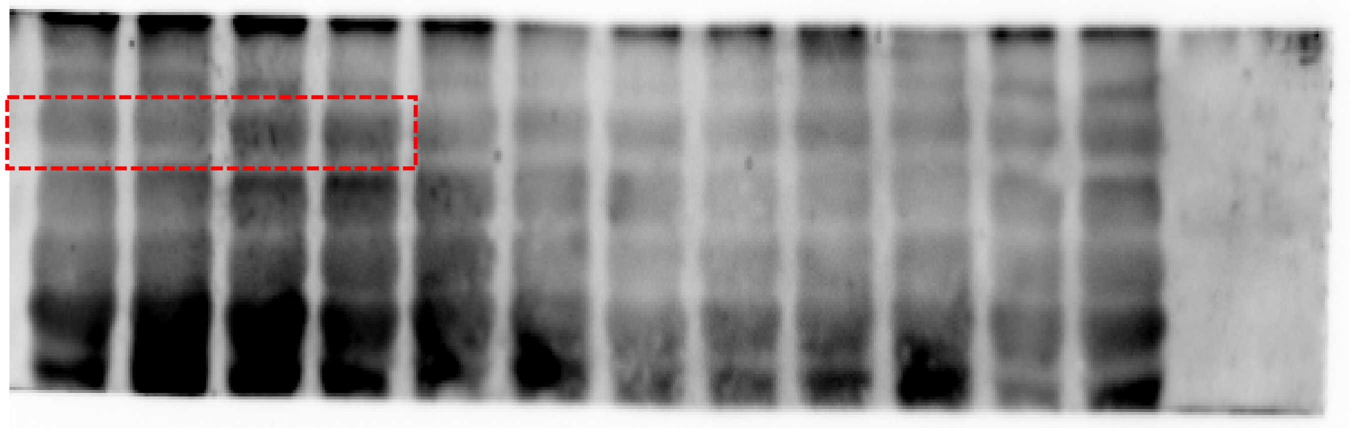

(2)

β-actin_2

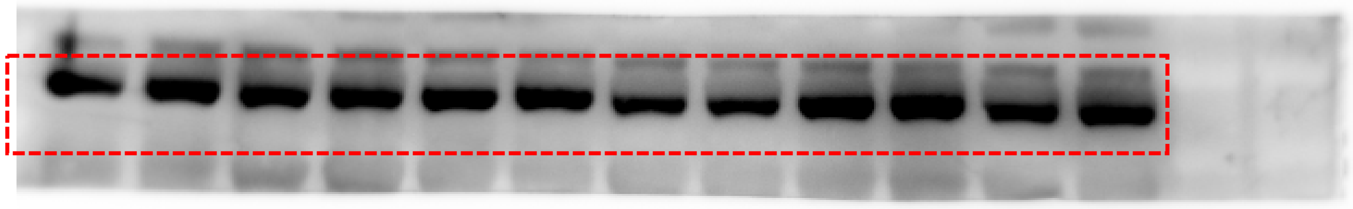

**Figure 6.(D)** Integrated transcriptomic profiling and proteomic verification reveal key adipogenic pathways underlying marbling grade differences (A1 vs A4) in longissimus dorsi muscle of Woking black cattle

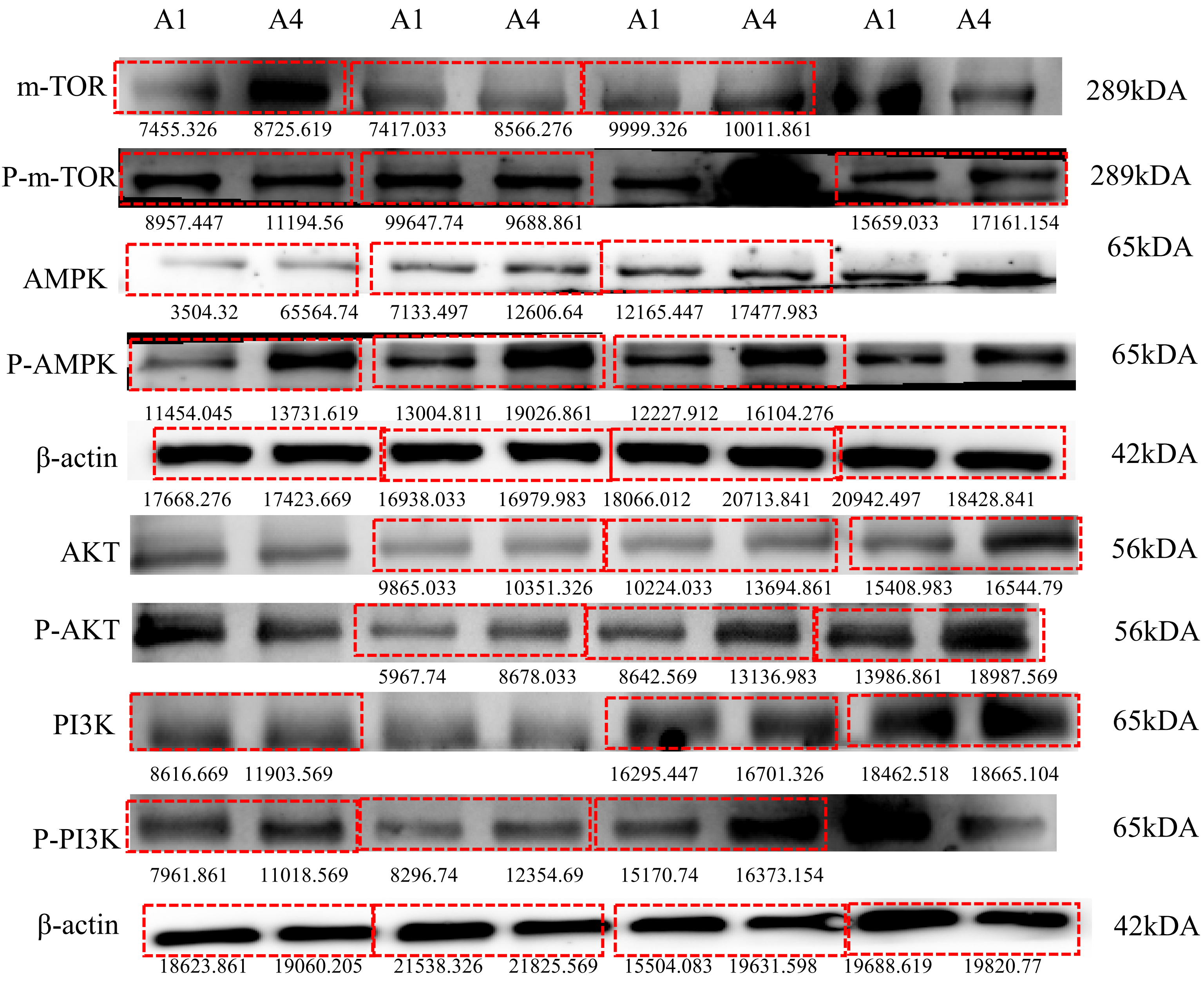

m-TOR

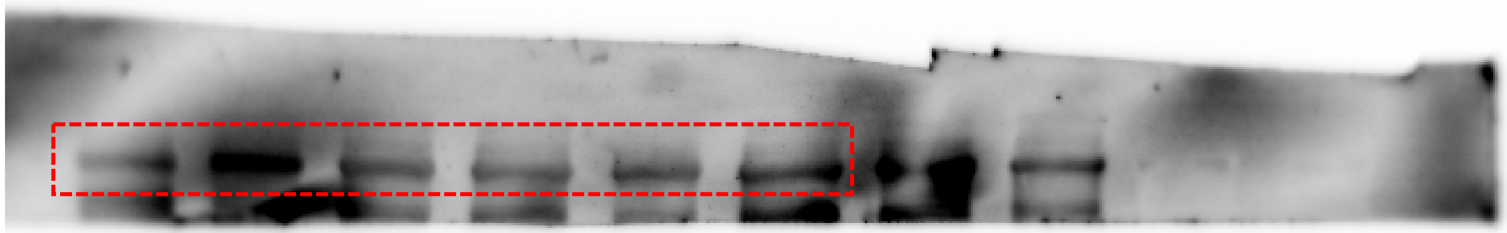

P-m-TOR

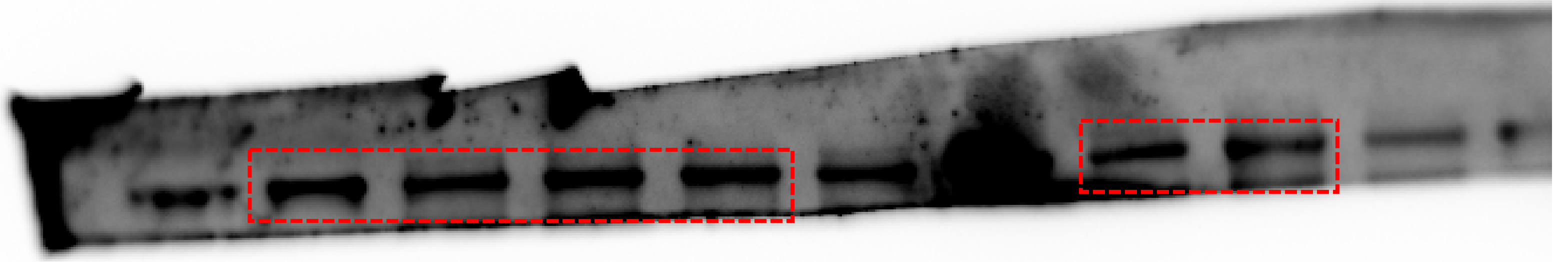

AMPK

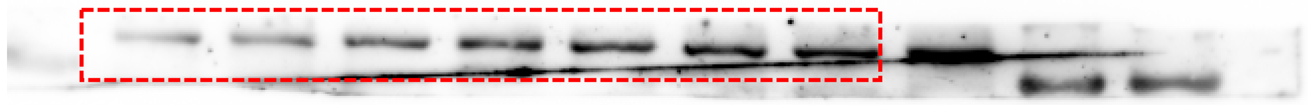

P-AMPK

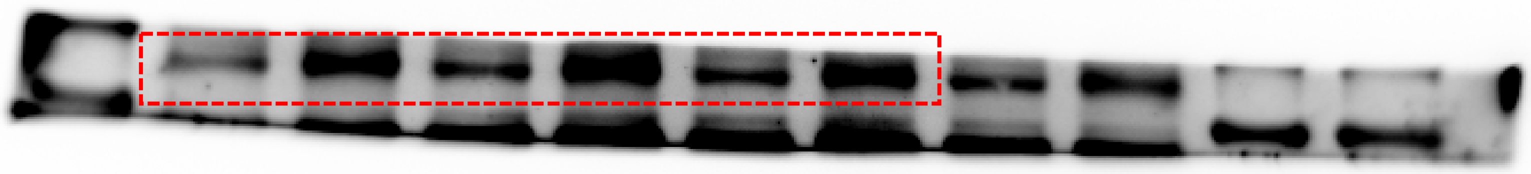

β-actin_1

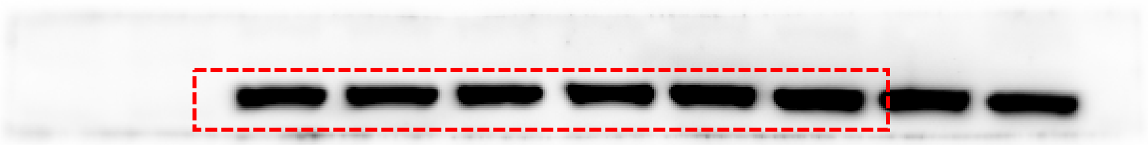

AKT

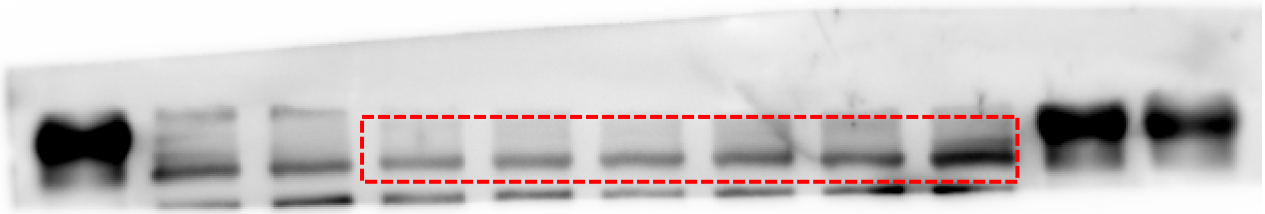

P-AKT

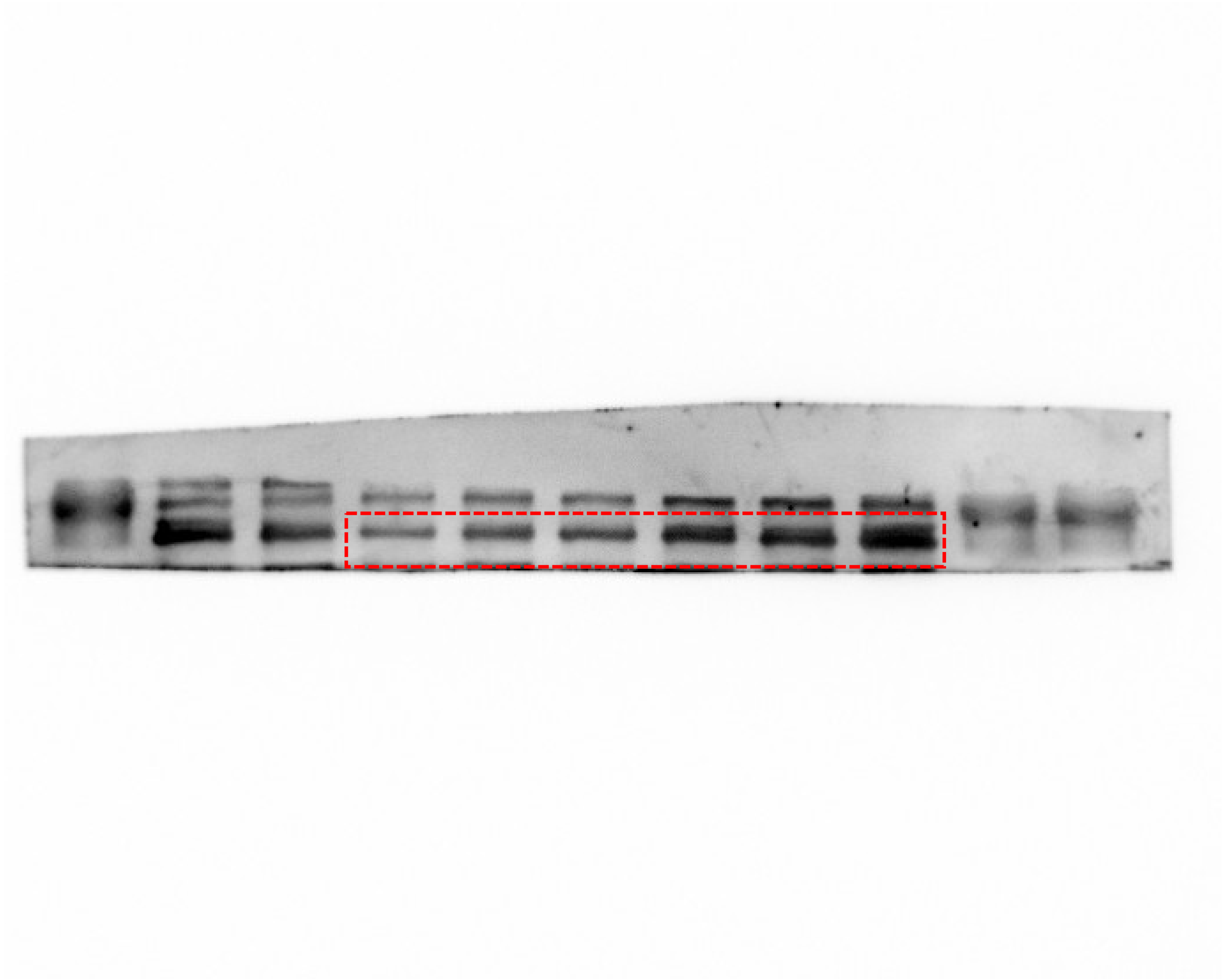

PI3K

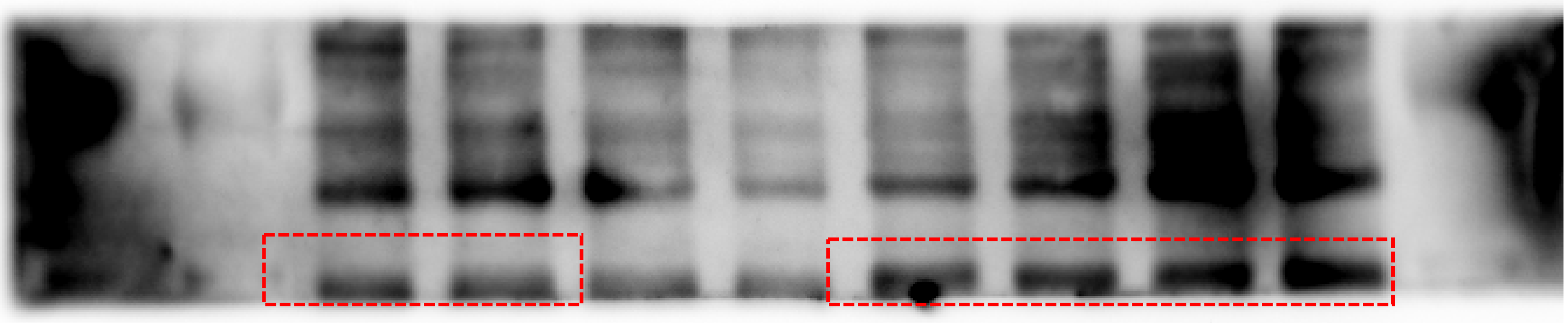

P-PI3K

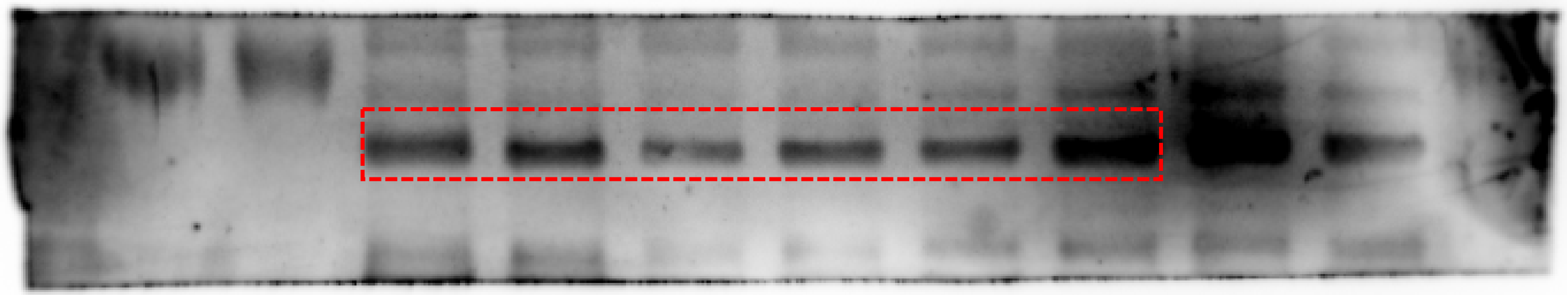

β-actin_2

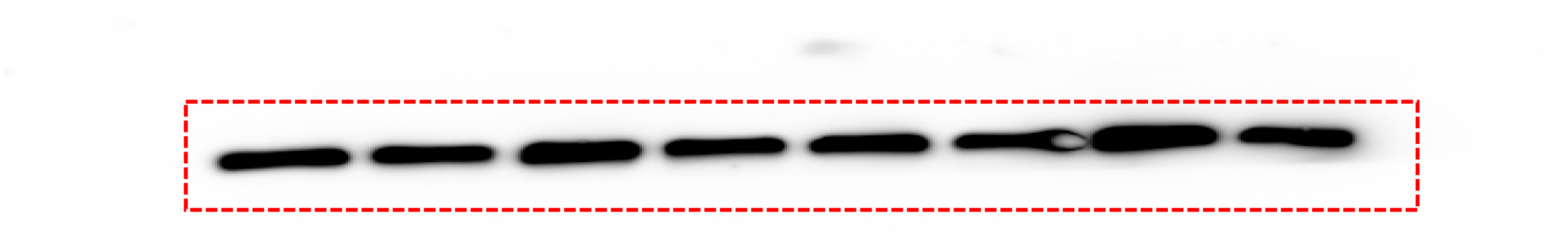

**Figure 6.(E)** Integrated transcriptomic profiling and proteomic verification reveal key adipogenic pathways underlying marbling grade differences (A1 vs A4) in longissimus dorsi muscle of Woking black cattle

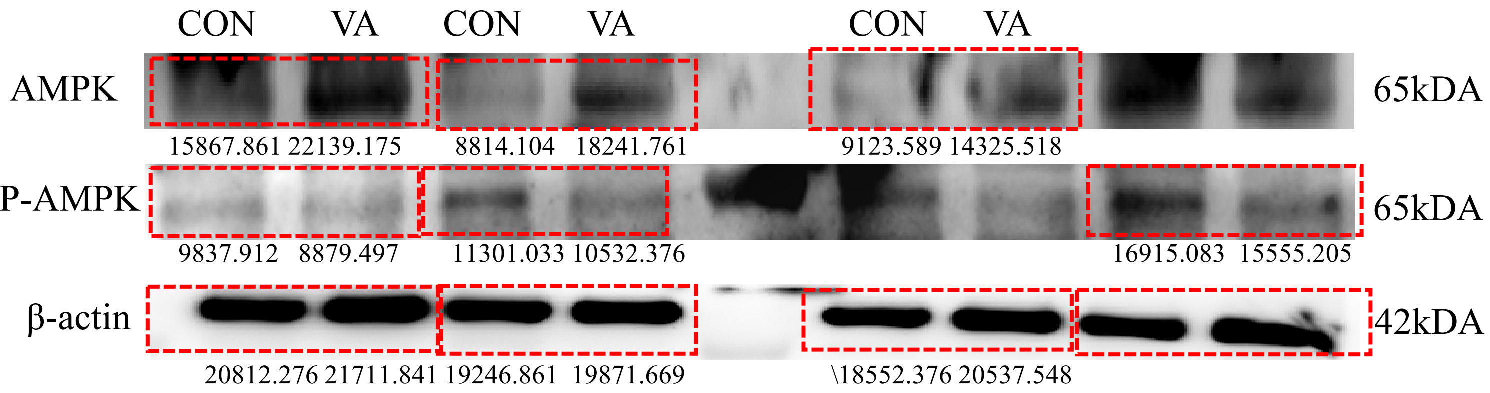

AMPK

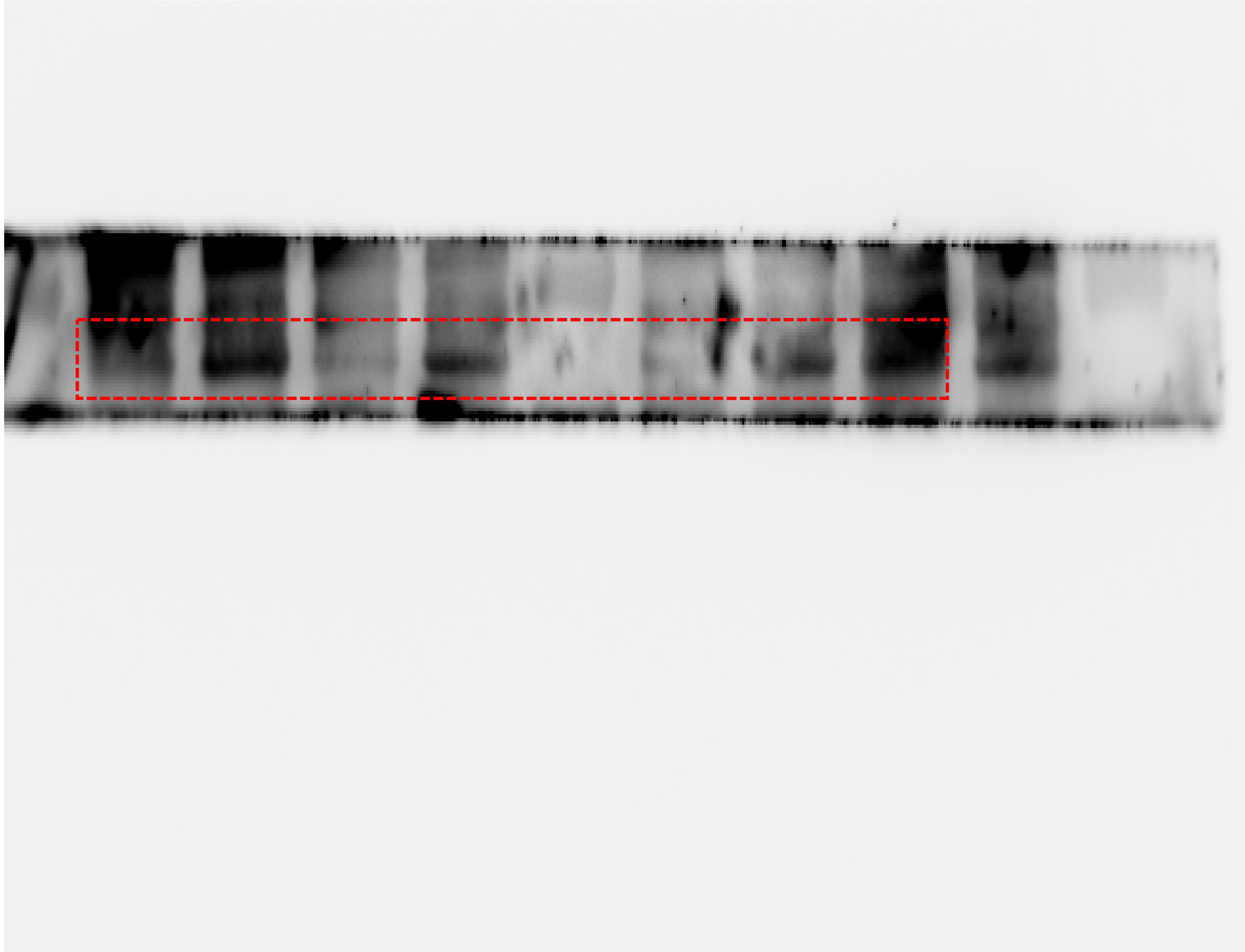

AMPK

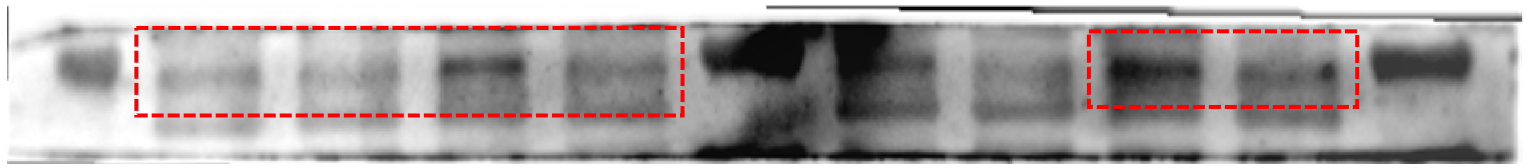

β-actin

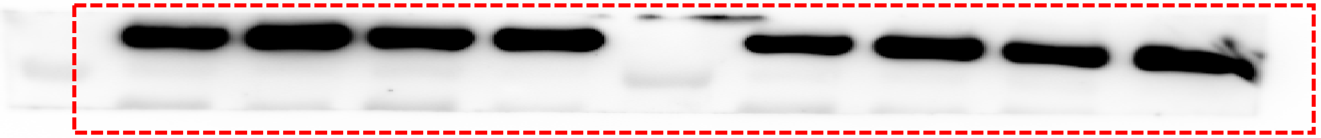

**Figure 7.** ATRA activates adipogenic pathways to promote triglyceride accumulation and marker expression in BSMCs of Woking black cattle.

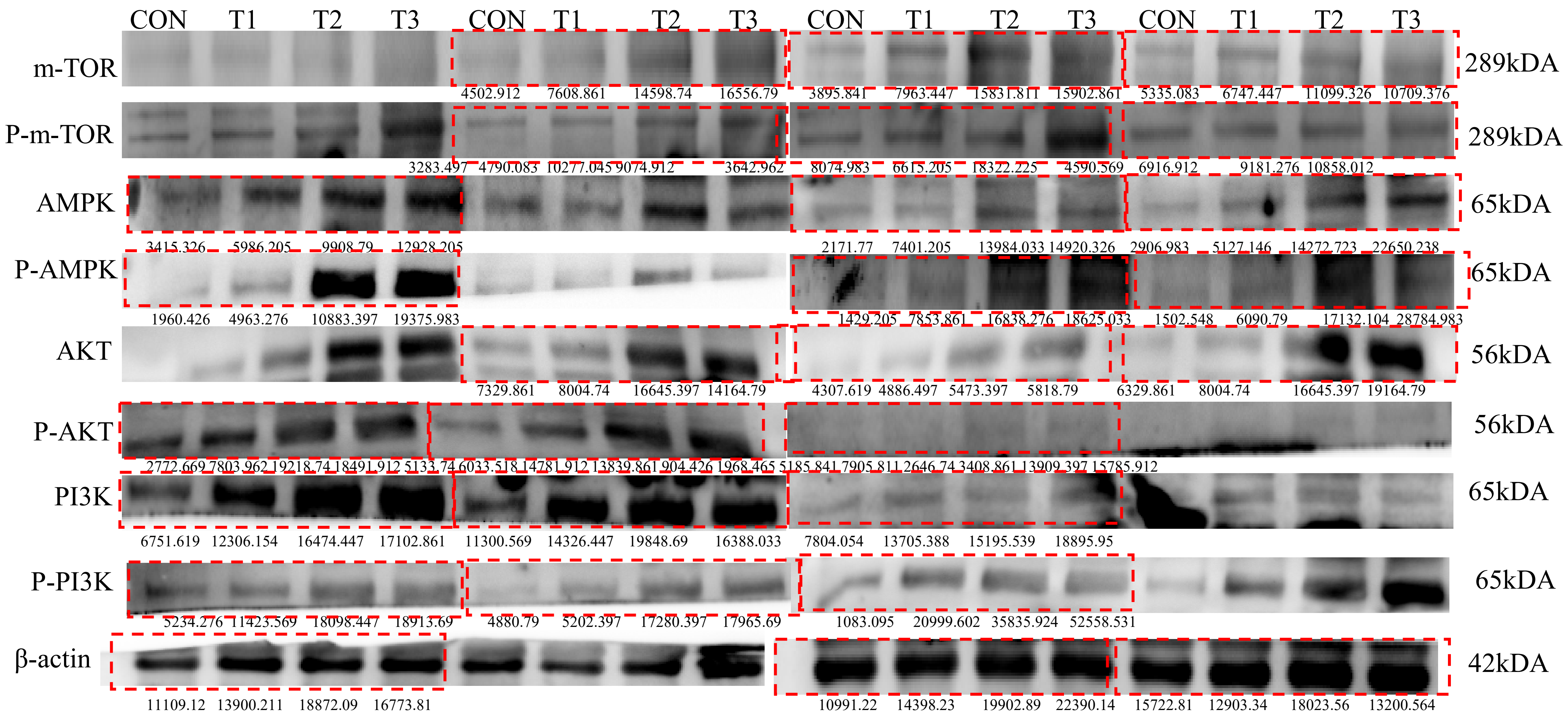

m-TOR

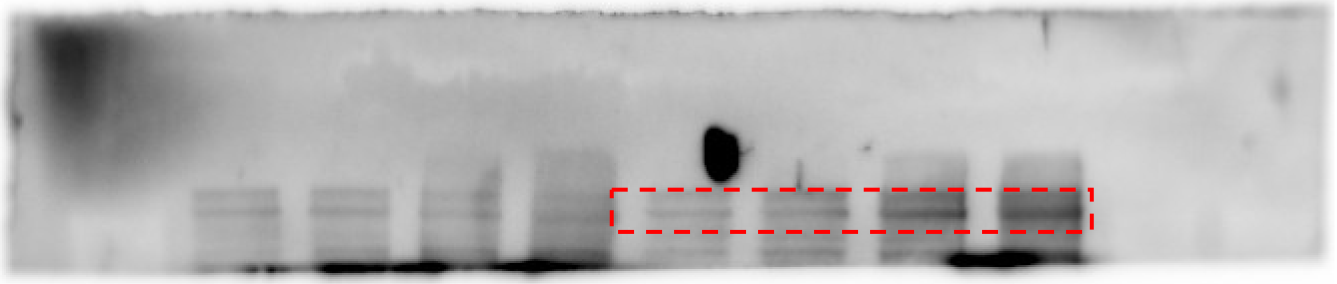

(1)

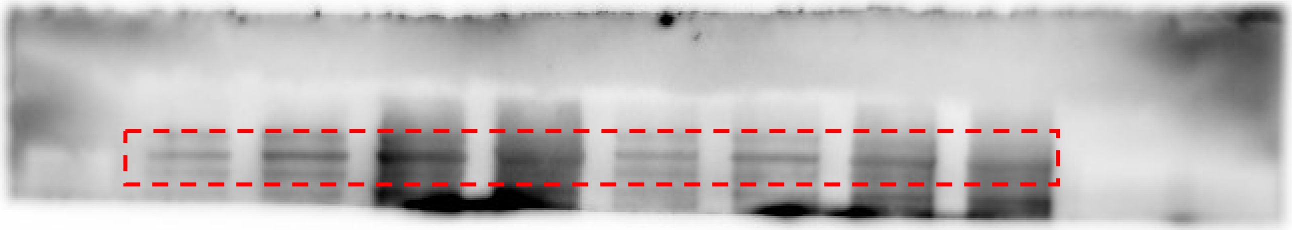

(2)

P-m-TOR

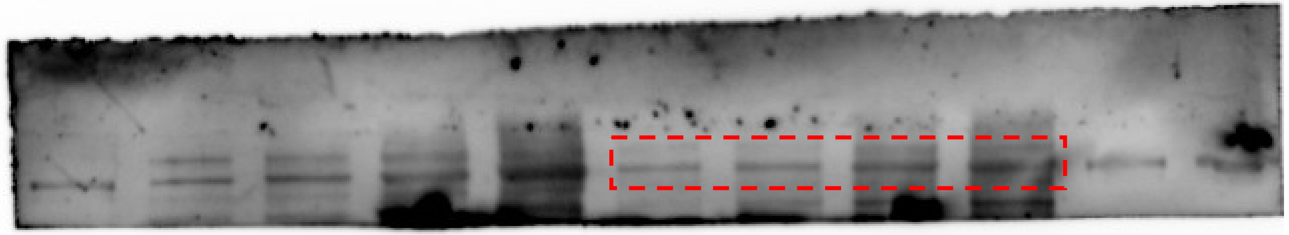

**(1)**

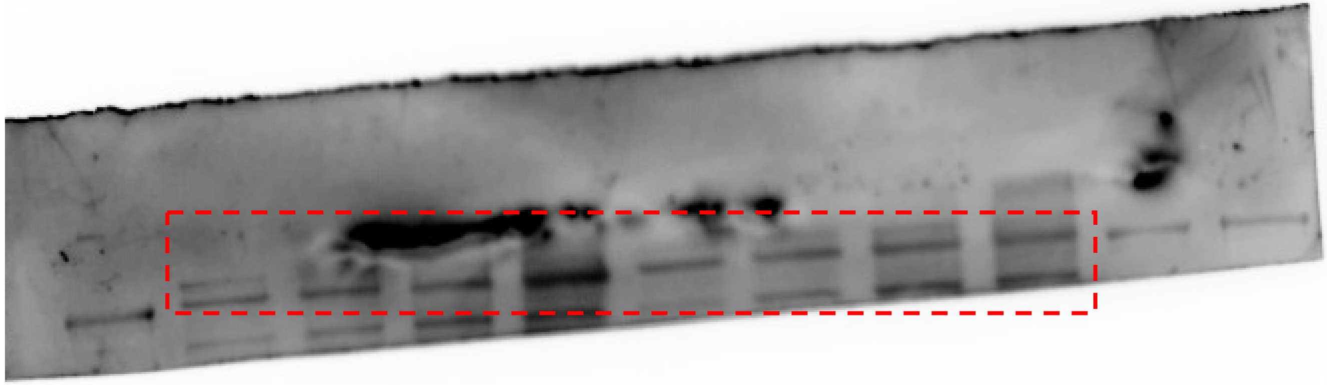

**(2)**

AMPK

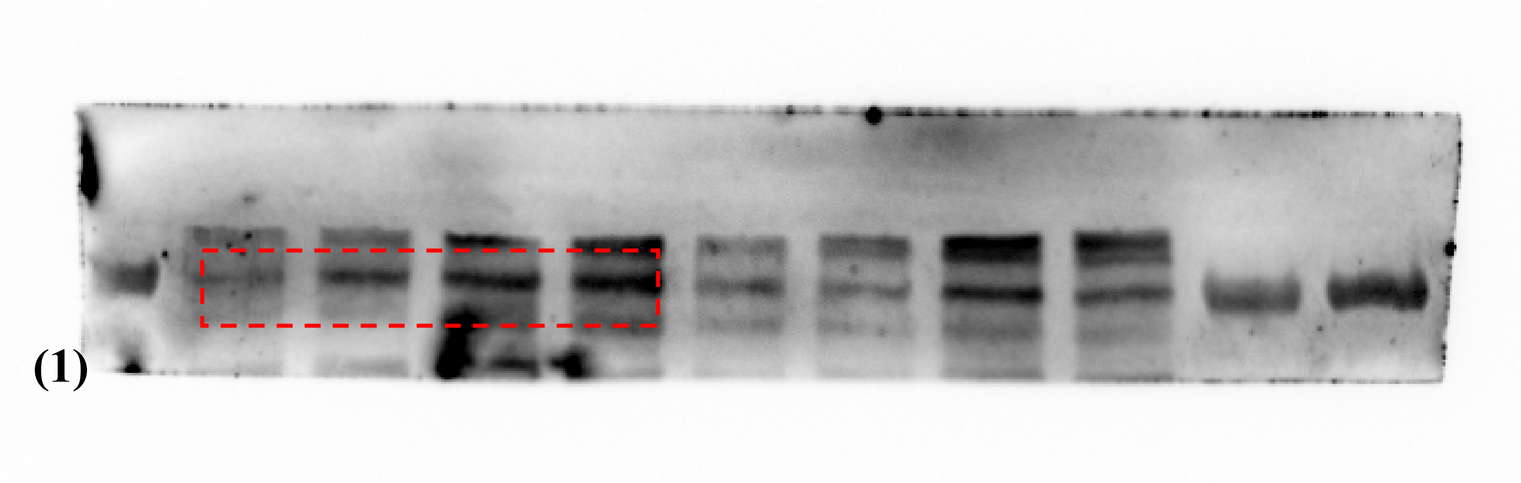

(1)

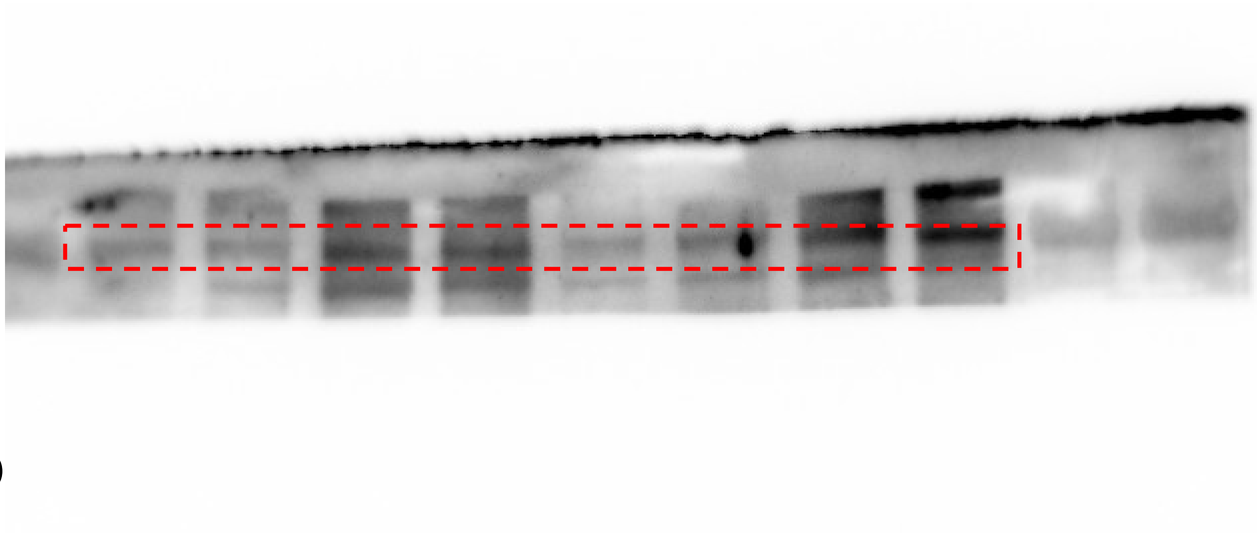

(2)

P-AMPK

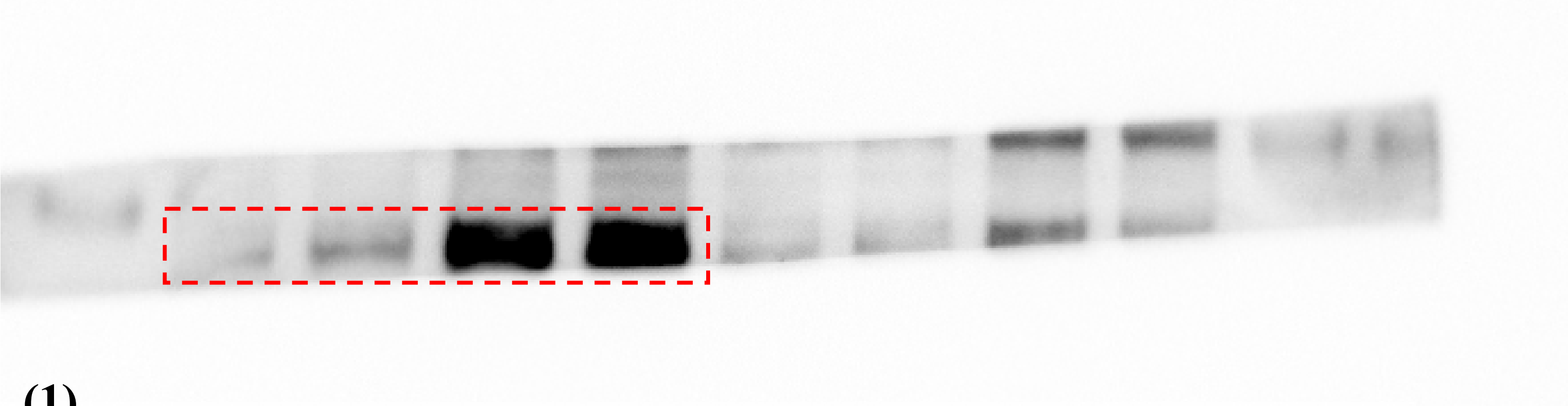

(1)

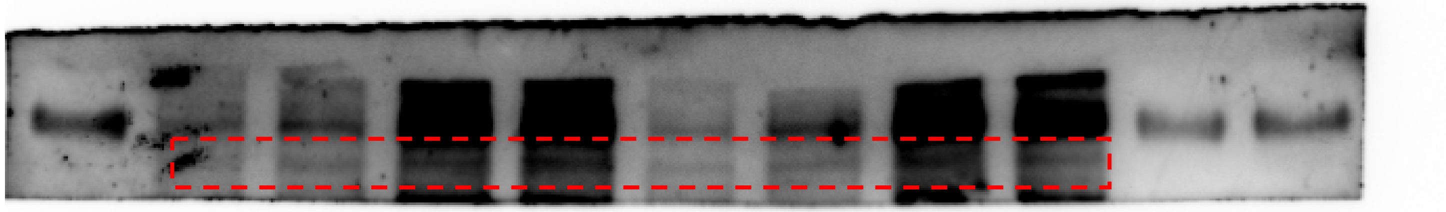

(2)

AKT

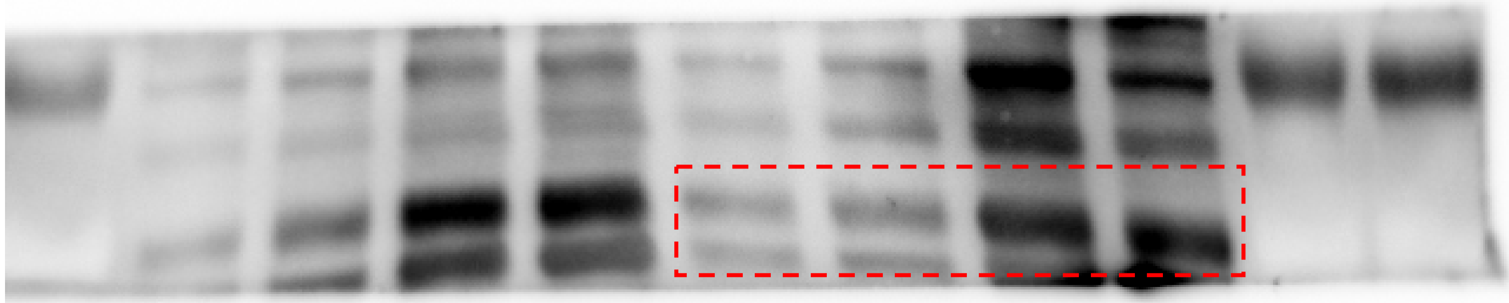

**(1)**

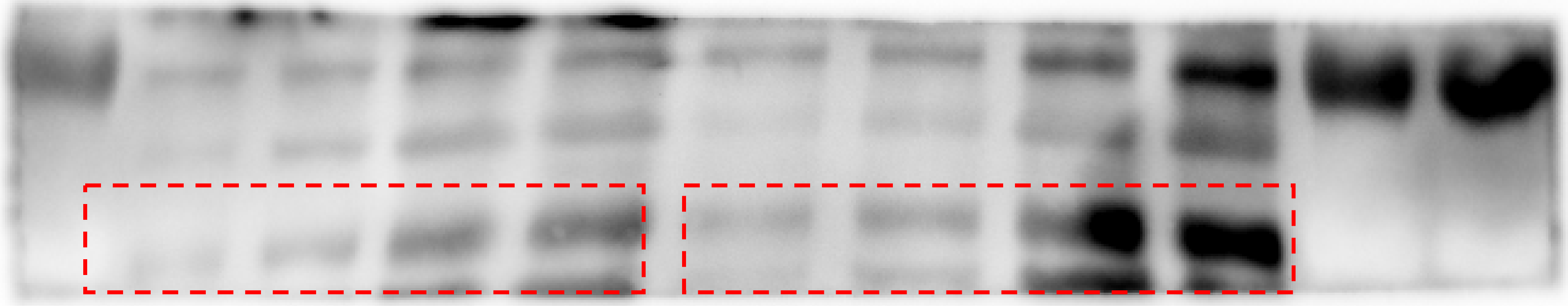

**(2)**

P-AKT

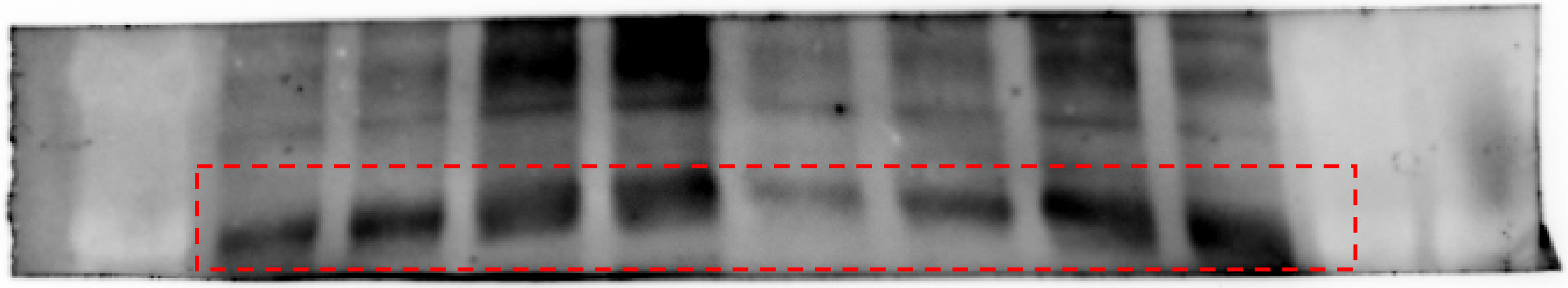

**(1)**

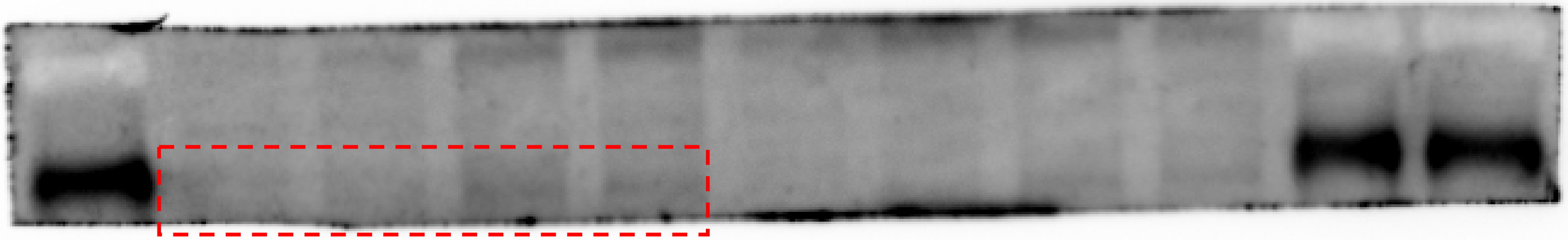

**(2)**

PI3K

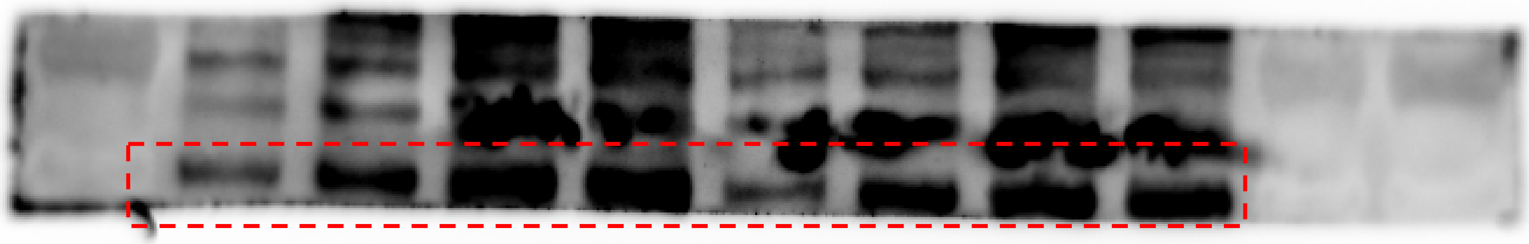

**(1)**

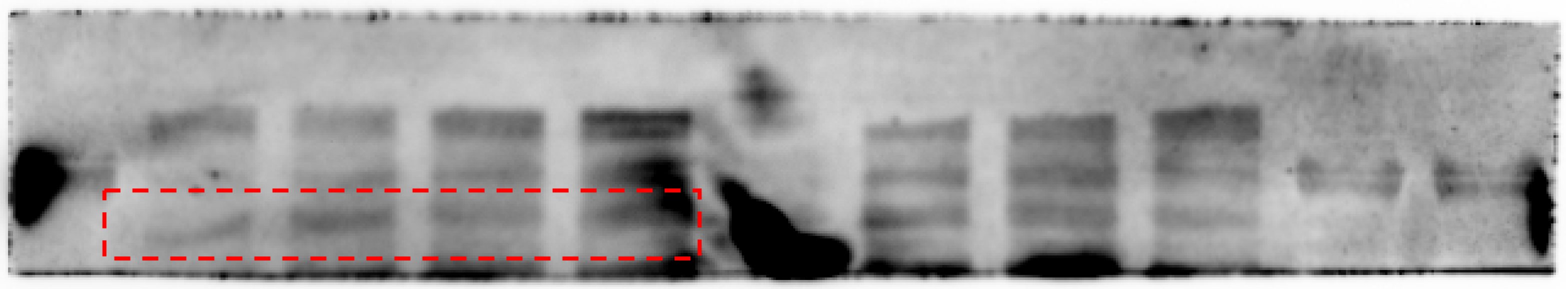

**(2)**

P-PI3K

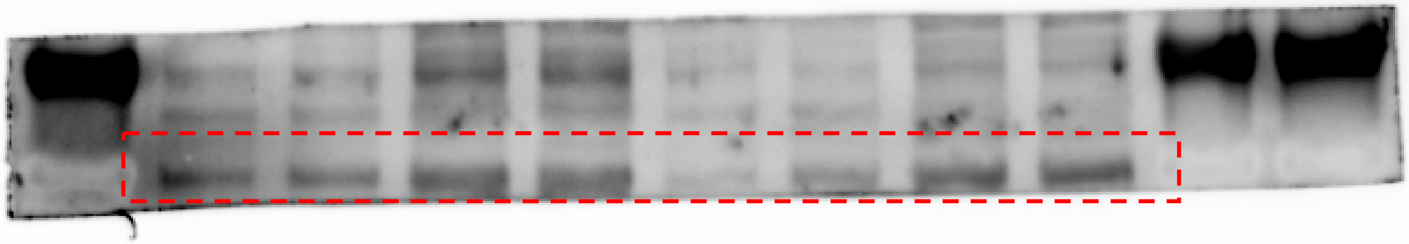

**(1)**

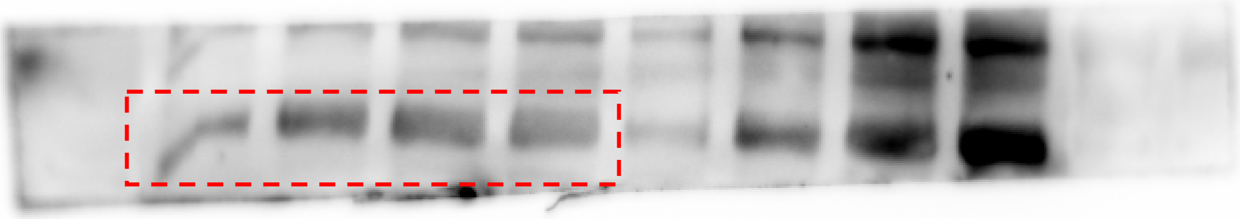

**(2)**

β-actin

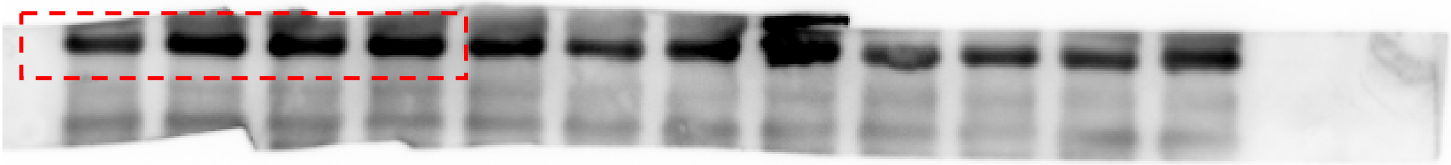

(1)

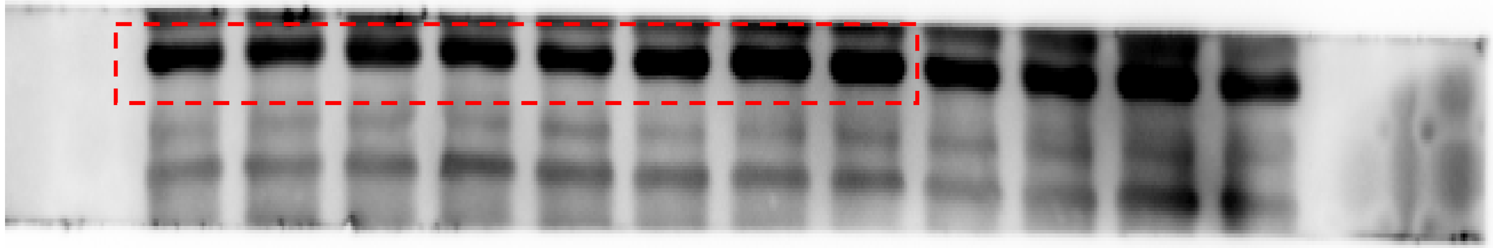

(2)

**Figure 8.**(B)Pharmacological Modulation of AMPK Activity Reveals Its Exclusive Mediating Role in Vitamin A-Induced Adipogenesis in BSMCs of Woking black cattle Evidence from Triglyceride Accumulation, Adipogenic Transcription, and Pathway Protein Expression

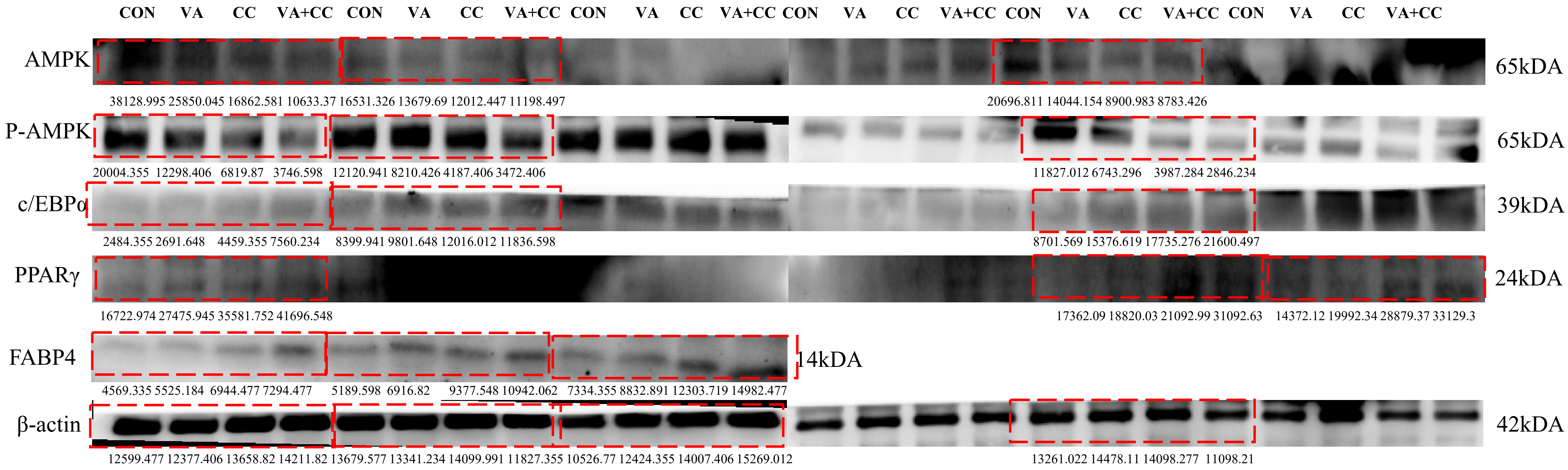

AMPK

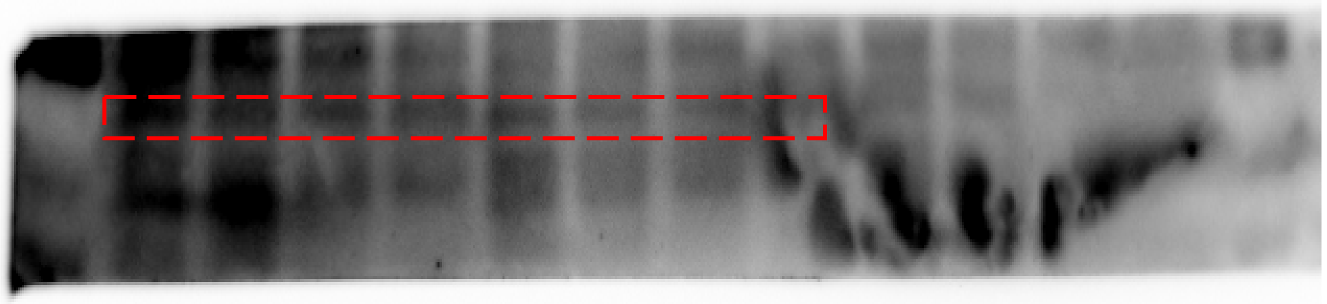

(1)

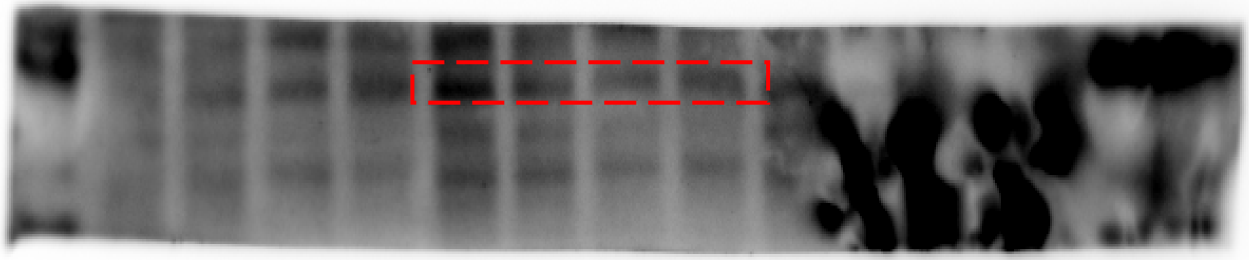

(2)

P-AMPK

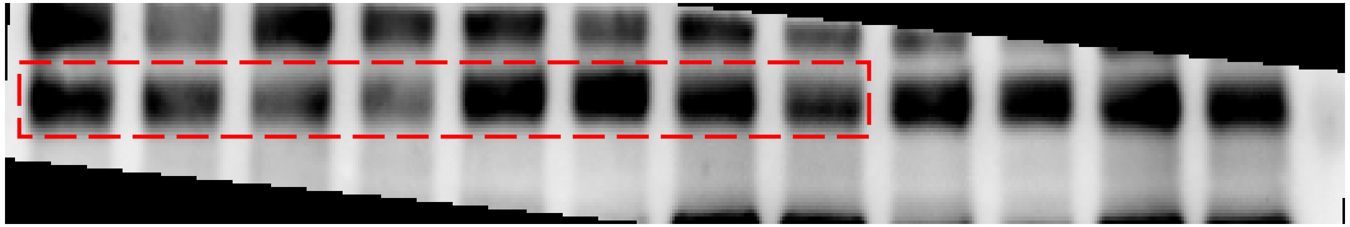

(1)

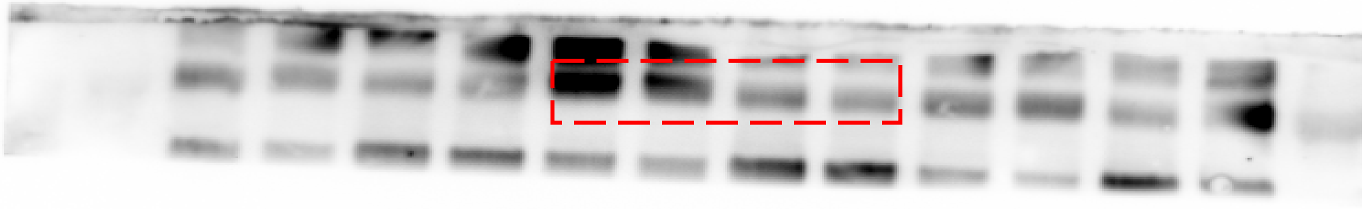

(2)

c/EBPα

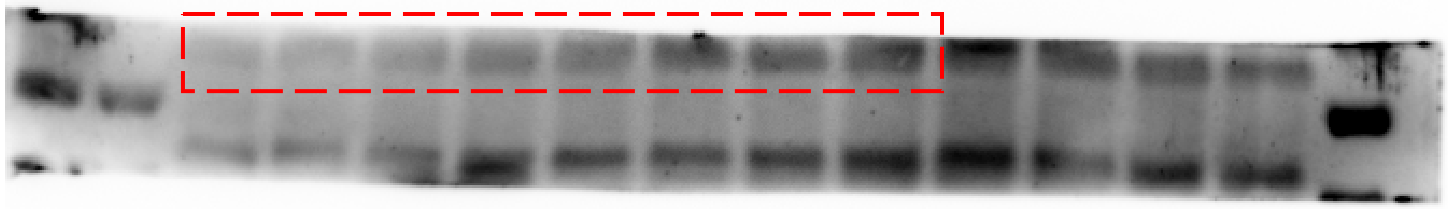

(1)

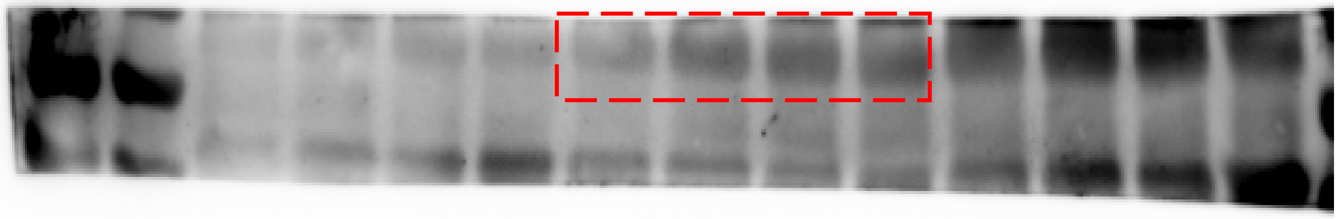

(2)

PPARγ

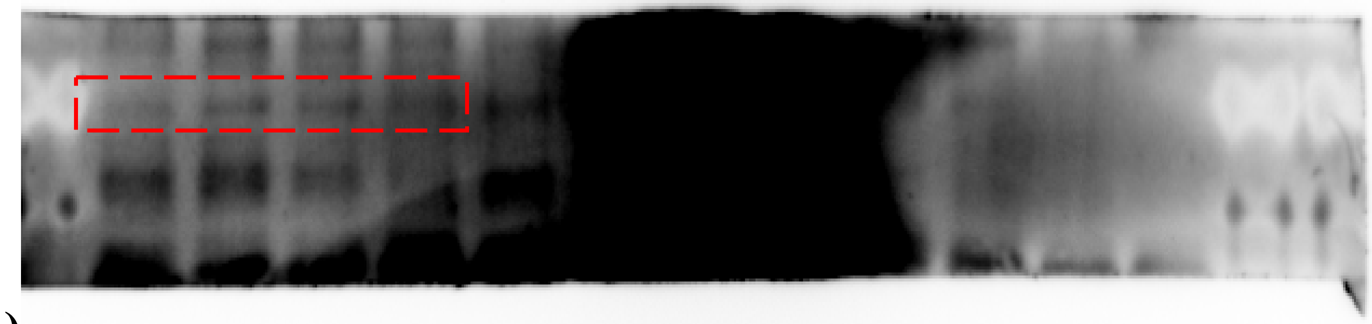

**(1)**

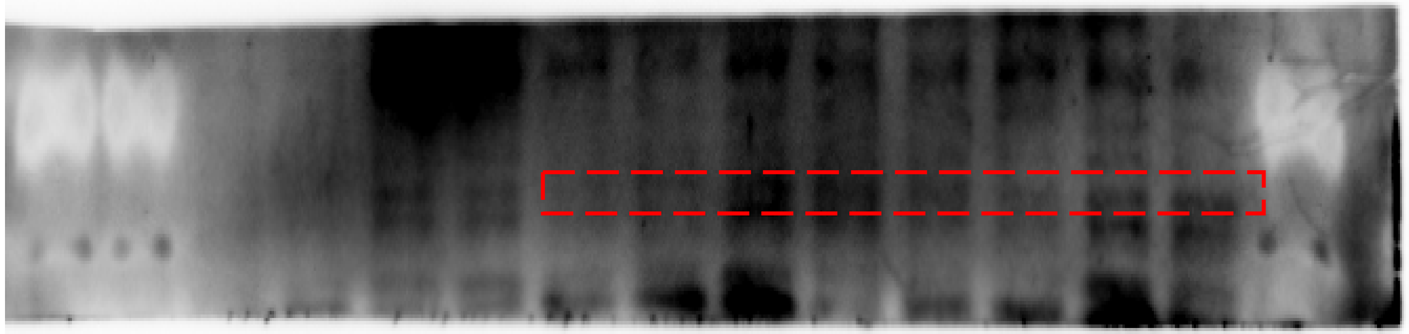

**(2)**

FABP4

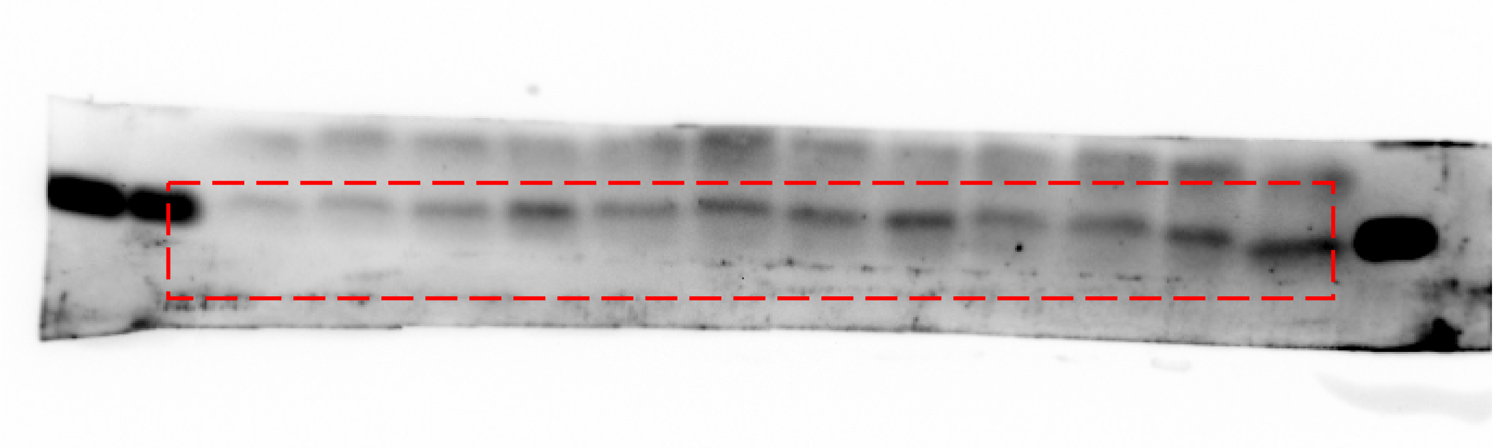

β-actin

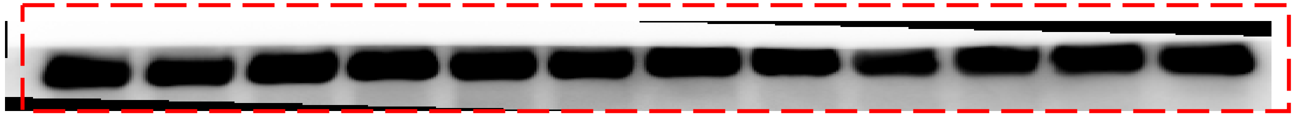

**(1)**

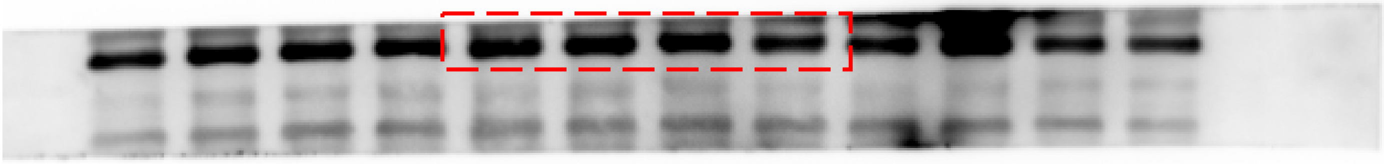

**(2)**

**Figure 8.**(D)Pharmacological Modulation of AMPK Activity Reveals Its Exclusive Mediating Role in Vitamin A-Induced Adipogenesis in BSMCs of Woking black cattle Evidence from Triglyceride Accumulation, Adipogenic Transcription, and Pathway Protein Expression

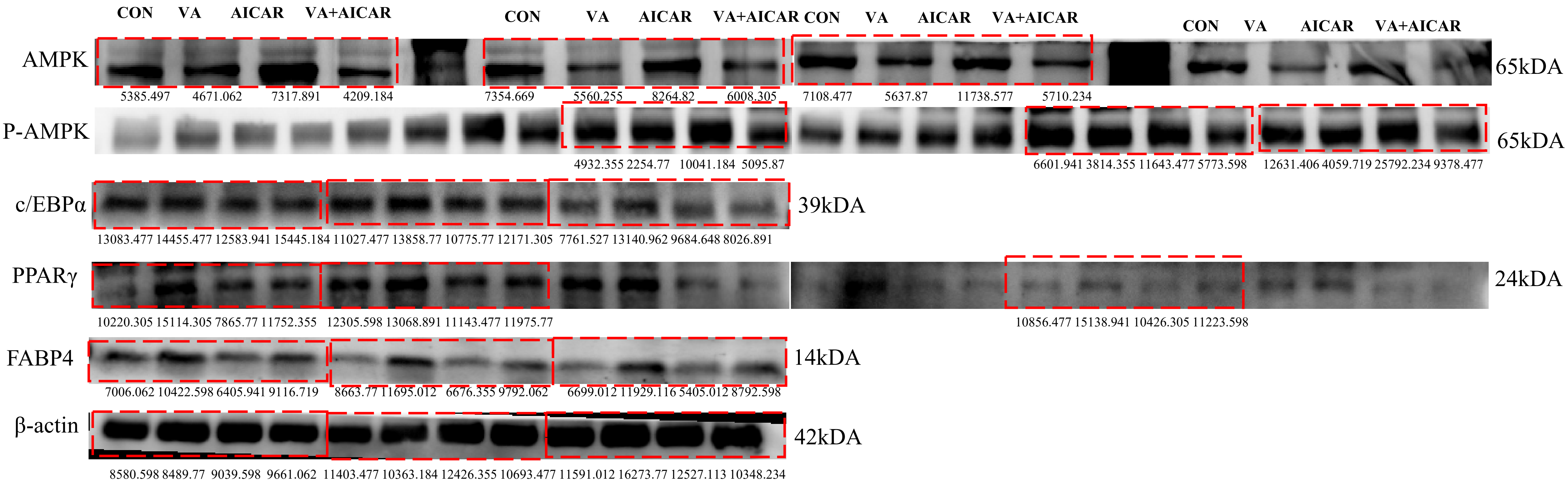

AMPK

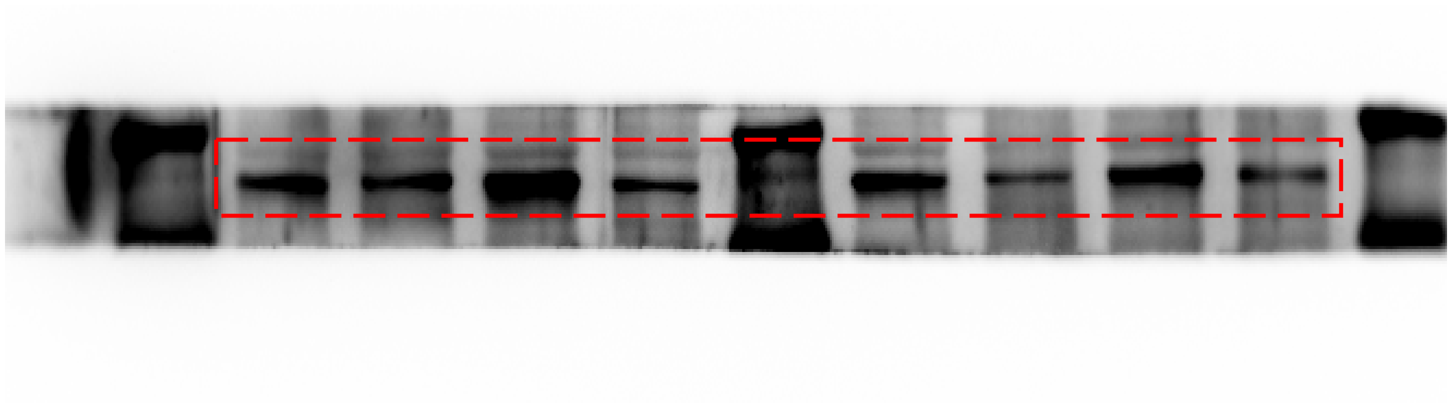

(1)

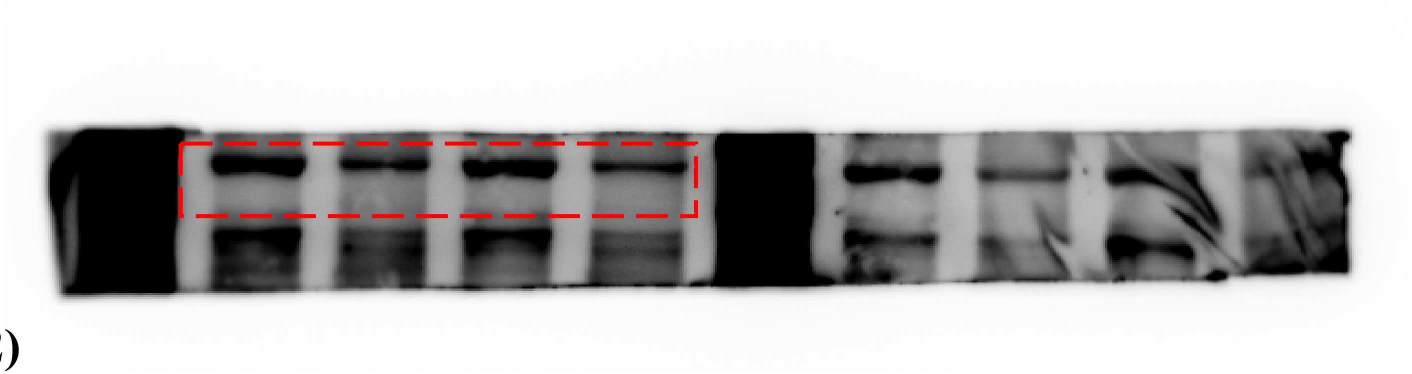

(2)

P-AMPK

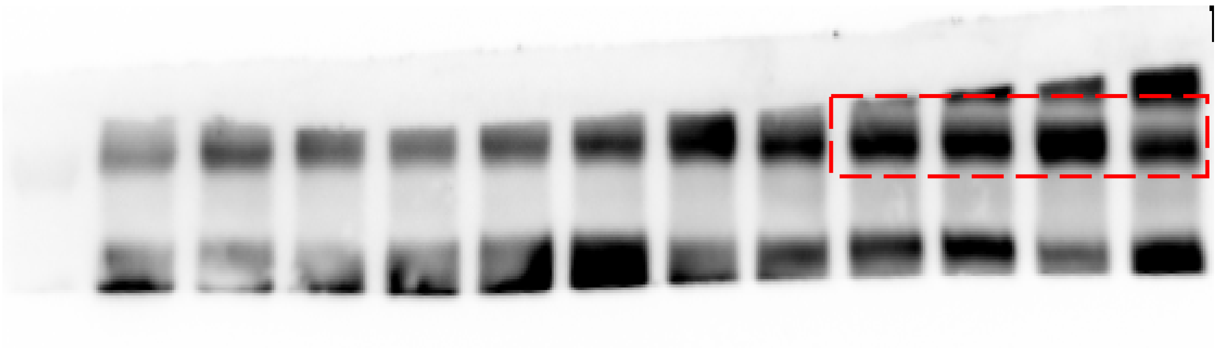

(1)

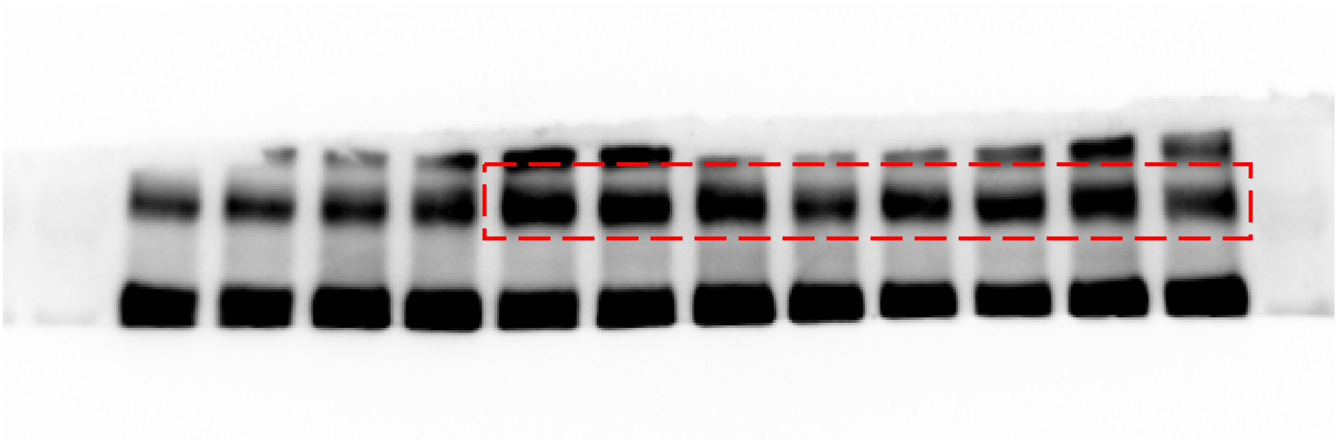

(2)

c/EBPα

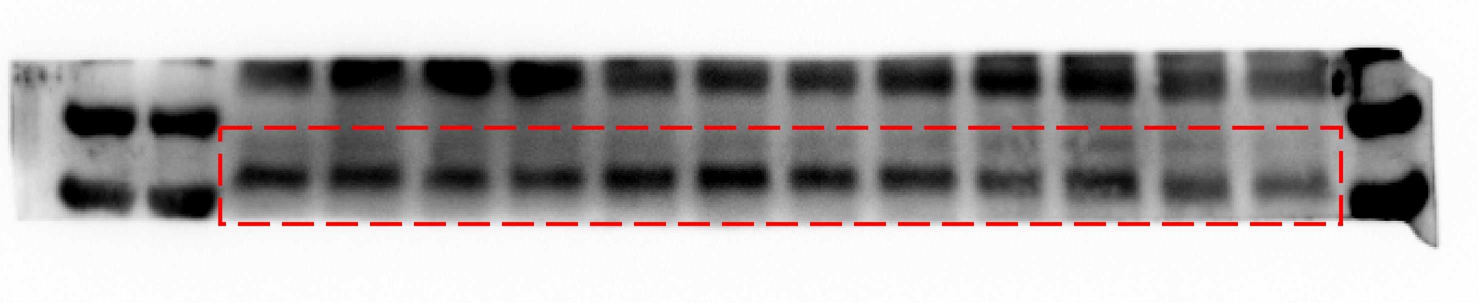

PPARγ

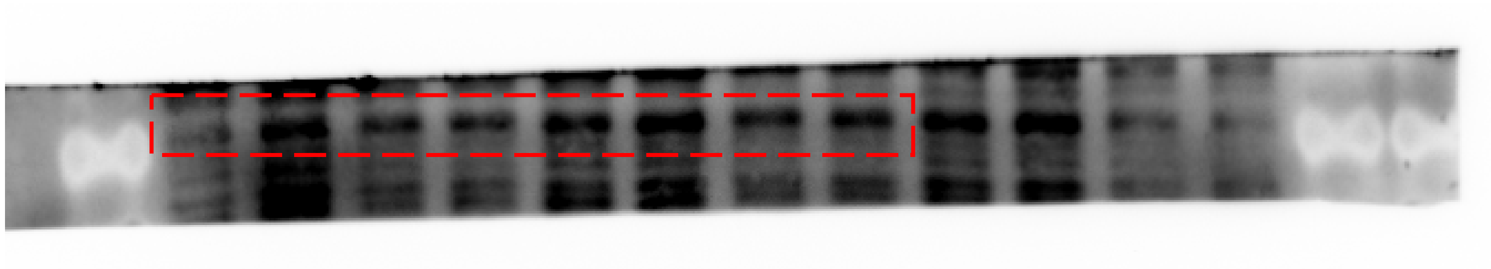

(1)

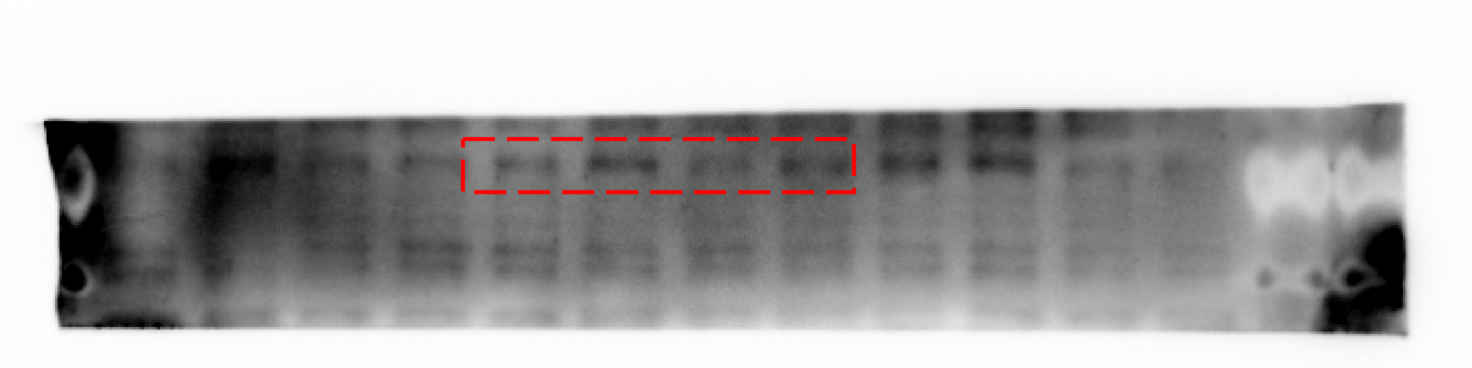

(2)

FABP4

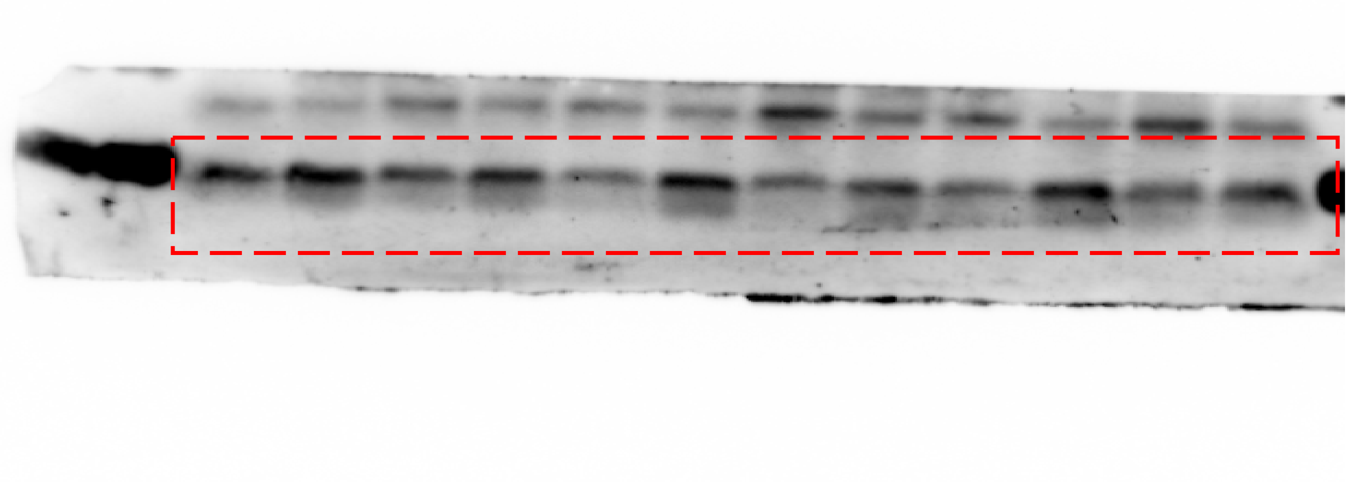

β-actin

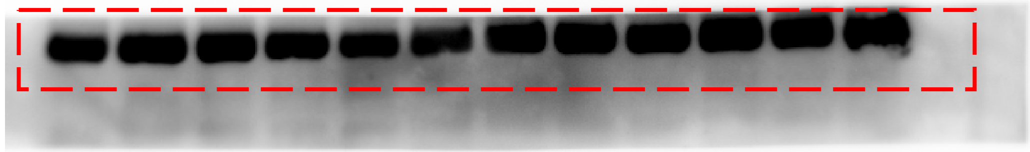

Supplement: Supplementary file 3 — Additional file 3. Original gels of the Western blots. [file 40104_2025_1343_MOESM3_ESM.pdf]
